# Exploring the disruption of SARS-CoV-2 RBD binding to hACE2

**DOI:** 10.3389/fchem.2023.1276760

**Published:** 2023-10-24

**Authors:** Camryn Carter, Justin Airas, Haley Gladden, Bill R. Miller, Carol A. Parish

**Affiliations:** ^1^ Department of Chemistry, Gottwald Center for the Sciences, University of Richmond, Richmond, VA, United States; ^2^ Department of Chemistry, Truman State University, Kirksville, MO, United States

**Keywords:** SARS-CoV-2, receptor binding domain, repurposed drugs, molecular dynamics, MM-GBSA, COVID-19, human ACE2 receptor, ACE inhibitors

## Abstract

The COVID-19 pandemic was declared due to the spread of the novel coronavirus, SARS-CoV-2. Viral infection is caused by the interaction between the SARS-CoV-2 receptor binding domain (RBD) and the human ACE2 receptor (hACE2). Previous computational studies have identified repurposed small molecules that target the RBD, but very few have screened drugs in the RBD–hACE2 interface. When studies focus solely on the binding affinity between the drug and the RBD, they ignore the effect of hACE2, resulting in an incomplete analysis. We screened ACE inhibitors and previously identified SARS-CoV-2 inhibitors for binding to the RBD—hACE2 interface, and then conducted 500 ns of unrestrained molecular dynamics (MD) simulations of fosinopril, fosinoprilat, lisinopril, emodin, diquafosol, and physcion bound to the interface to assess the binding characteristics of these ligands. Based on MM-GBSA analysis, all six ligands bind favorably in the interface and inhibit the RBD–hACE2 interaction. However, when we repeat our simulation by first binding the drug to the RBD before interacting with hACE2, we find that fosinopril, fosinoprilat, and lisinopril result in a strongly interacting trimeric complex (RBD-drug-hACE2). Hydrogen bonding and pairwise decomposition analyses further suggest that fosinopril is the best RBD inhibitor. However, when lisinopril is bound, it stabilizes the trimeric complex and, therefore, is not an ideal potential drug candidate. Overall, these results reveal important atomistic interactions critical to the binding of the RBD to hACE2 and highlight the significance of including all protein partners in the evaluation of a potential drug candidate.

## Introduction

In December 2019, a novel coronavirus, severe acute respiratory syndrome coronavirus 2 (SARS-CoV-2), was identified in Wuhan, China (Wang et al., 2020a). The spread of the virus caused a global outbreak of the infectious disease, coronavirus disease 2019 (COVID-19), which the World Health Organization declared the COVID-19 pandemic (Pascarella et al., 2020). Currently, more than 1 million people in the United States (US) have died from SARS-CoV-2 infection, and about 104 million cases have been confirmed in the US. Viral infection of SARS-CoV-2 is due to the interaction between the SARS-CoV-2 spike protein and the human angiotensin-converting enzyme 2 (hACE2) receptor (Wu et al., 2020).

The hACE2 receptor, located on the surface of human throat and lung epithelial cells, is the target for SARS-CoV-2 (Mittal et al., 2020). In a healthy human, hACE2 is responsible for regulating heart function and the renin-angiotensin system (RAS). hACE2 hydrolyzes angiotensin II to its active form, angiotensin 1–7, which counterbalances the adverse effects of the RAS (Burrell et al., 2004; Jiang et al., 2014; Cruz-Diaz et al., 2017; Wang et al., 2020b). SARS-CoV-2 contains spike-like projections of glycoproteins on its surface that are responsible for host cell infection. Specifically, the ectodomain of the SARS-CoV-2 virus consists of a receptor binding subunit (S1) which contains the receptor binding domain (RBD), residues 331–528, and a membrane-fusion subunit (S2) (Zhang et al., 2021). The SARS-CoV-2 RBD consists of a section of amino acids, residues 438–506, referred to as the receptor binding motif (RBM), that interact directly with hACE2 (Yi et al., 2020).

To prevent SARS-CoV-2 infection or lessen the symptoms of COVID-19, researchers have developed vaccines, antivirals, and other therapeutics. The US Food and Drug Administration (FDA) approved the bivalent mRNA vaccines developed by Pfizer-BioNTech and Moderna and issued emergency use authorization (EUA) for the adjuvanted subunit vaccine developed by Novavax (Creech et al., 2021; U. S. Food and Drug Administration, 2023). The antibodies produced as a result of these vaccines can target the RBD and are capable of prohibiting the virus from interfering with hACE2 (Krammer, 2020; Mascellino et al., 2021). Additionally, over the past few years, effective SARS-CoV-2 antiviral therapies have been produced and are continuing to be developed. These antivirals inhibit SARS-CoV-2 enzymes, such as the SARS-CoV-2 polymerase or protease, or inhibit SARS-CoV-2 viral entry into host cells. Inhibitors such as remdesivir, tocilizumab, and baricitinib are FDA approved (Kalil et al., 2021; Kulanthaivel et al., 2021). Previously developed vaccines and antivirals are extremely effective in reducing hospitalizations and deaths among vaccinated individuals (Shang et al., 2020; Tenforde et al., 2021). Despite these advances, there is a continued need to explore the molecular nature of this virus and identify a drug capable of disrupting the RBD - hACE2 interface.

Previous works have studied the behavior of novel and repurposed drugs as SARS-CoV-2 inhibitors targeting the spike protein (Br et al., 2020; Jaiswal and Kumar, 2020; Wang et al., 2022), RNA polymerase (El Hassab et al., 2020; Parvez et al., 2020; Elkarhat et al., 2022), or the main protease enzyme (Alnajjar et al., 2020; Teli et al., 2021; Airas et al., 2022). The RBD of the spike protein is an often-used target in order to inhibit its interaction with hACE2 receptors, thereby preventing viral infection. Many studies have identified potential inhibitors using molecular docking and/or molecular dynamics (MD) simulations against solely the RBD (Faheem et al., 2020; Prajapat et al., 2020; Trezza et al., 2020; Kumar et al., 2021; Hu et al., 2022; Wu et al., 2022) or solely the hACE2 receptor (Benítez-Cardoza and Vique-Sánchez, 2020; Kabir et al., 2021). Very few studies have analyzed the interaction of small drug-like molecules with the RBD–hACE2 interface. Al-Karmalawy et al. screened angiotensin-converting enzyme (ACE) inhibitors against the RBD - hACE2 interface and found lisinopril and alacepril to be a promising inhibitor candidates (Al-Karmalawy et al., 2021). The work of Issac-Lam screened ACE inhibitors in addition to ACE2 blockers, blood thinning agents, cholesterol-lowering medications, repurposed drugs, and remdesivir against the RBD, hACE2, and the interface. Only nine ligands were successfully docked at the interface, including three ACE inhibitors: benazepril, captopril, and fosinopril. However, each ACE inhibitor was found to have a relatively low docking score for the interface and preferred to bind to hACE2 (except benazepril) (Isaac-Lam, 2021).

Molecular simulations to evaluate repurposed small molecules as effective SARS-CoV-2 therapeutics are a significant area of research. Ideally, repurposed drug-like ligands should bind well to the interface between the RBD and hACE2. The protein interaction with the drug should be specific and favorable so that the drug can inhibit the dimeric complex and not dissociate from the binding site. Much of the previous work in this area has focused solely on the ability of the drug to bind to the RBD. However, studying only this binding affinity ignores the effects hACE2 has on that interaction. Additionally, small molecules that favorably interact with the dimer complex can also stabilize the RBD - hACE2 interaction instead of inhibiting it, forming a stable trimeric complex.

We were interested in evaluating whether repurposed ACE inhibitors could be used as SARS-CoV-2 RBD inhibitors and to better understand if, under those conditions, the RBD - hACE2 molecular interactions could be disrupted. ACE inhibitors are used to treat cardiovascular and renal disease and are known to reduce hypertension and congestive heart failure (Brown and Vaughan, 1998). They function by inhibiting the ability of the angiotensin-converting enzyme (ACE) to make angiotensin II, a blood vessel constricting agent. Inhibition of the ACE enzyme results in widened blood vessels, less pulmonary effort and lower blood pressure (Zisman, 2005). Experimental studies suggest they do not inhibit ACE2 function and were chosen in this study as a proof-of-concept for understanding how small ligands interact with the RBD - hACE2 interface (Coates, 2003; Ferrario et al., 2005). In addition to studying ACE inhibitors binding to the interface, we also compared the binding ability of small molecule SARS-CoV-2 drugs identified by LSBio (Yi et al., 2020) and additional reports (Hijikata et al., 2020; Sternberg et al., 2020). All ligands explored in this study are depicted in Tables 1, 2. These ligands were selected for this study as we were seeking to find previously approved drugs that could be quickly repurposed for use in combating COVID-19. We chose ACE inhibitors as the ACE2 binding site is homologous to one of the ACE active sites and we added ligands shown to be SARS-CoV-2 spike inhibitors (Prabakaran et al., 2004). We utilized the experimental structure (6LZG) for the wild type (WT) RBD - hACE2 interface as that was the only structure available when we began this project. As this project progressed, the Omicron RBD mutation has become the dominant infectious strain, but reports suggest that other than the mutation at position 493, RBD - hACE2 interaction is the same for WT and Omicron (Carter et al., 2023). Based on the molecular docking results, MD simulations were conducted of the following ligands interacting with the RBD–hACE2 interface: diquafosol, emodin, fosinopril, fosinoprilat, lisinopril, and physcion. After analyzing the estimated binding free energies using the molecular mechanics-generalized born surface area solvation (MM-GBSA) analysis of the apo and ligand-bound complexes, we found that all ligands bind favorably to the interface, and all but physcion exhibited inhibitory or disruptive effects whereby the RBD–hACE2 interaction was less favorable in the presence of the ligand, i.e., a more positive binding RBD–hACE2 free energy compared to the apo energy. Additionally, we found that fosinopril, fosinoprilat, and lisinopril displayed the best combination of low percent dissociation and favorable binding to RBD–hACE2. We selected these ACE inhibitors for subsequent hydrogen bonding and pairwise decomposition analyses to better understand their atomistic interactions in the interface. We found that each of these three ligands were able to significantly reduce the number of hydrogen bonds between the RBD and hACE2, with fosinopril acting as the best inhibitor. However, lisinopril and a specific starting pose of fosinoprilat were able to strengthen and stabilize the trimeric complex more so than the apo complex. Overall, we provide a detailed atomistic evaluation of how these specific ACE inhibitors perform as SARS-CoV-2 RBD inhibitors.

**TABLE 1:** Structures of ACE inhibitors selected for analysis.

Drug structure - ACE inhibitors		
Benazepril	Fosinoprilat	Quinapril
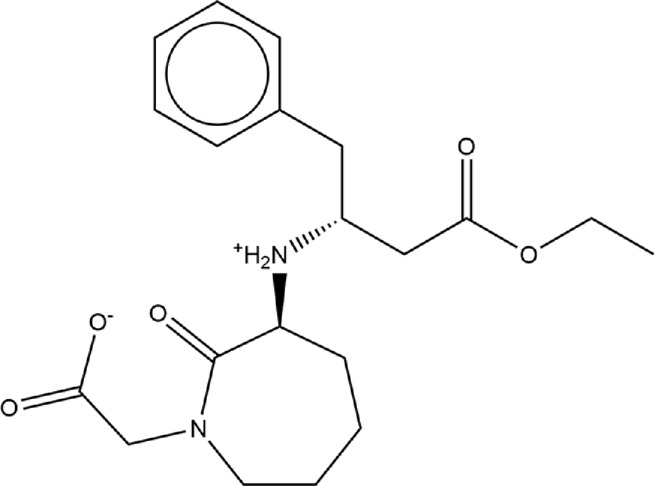	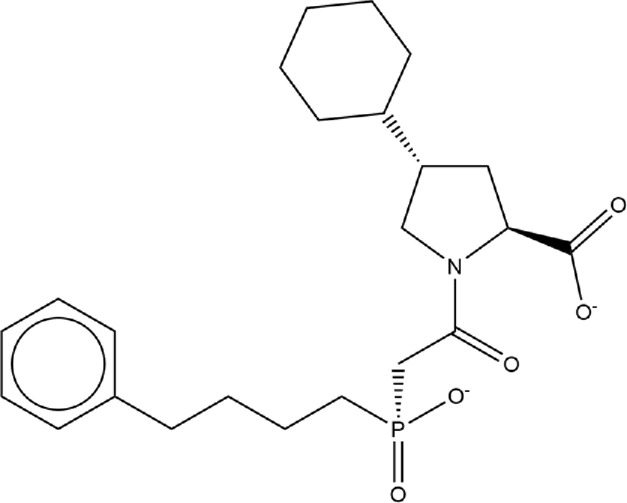	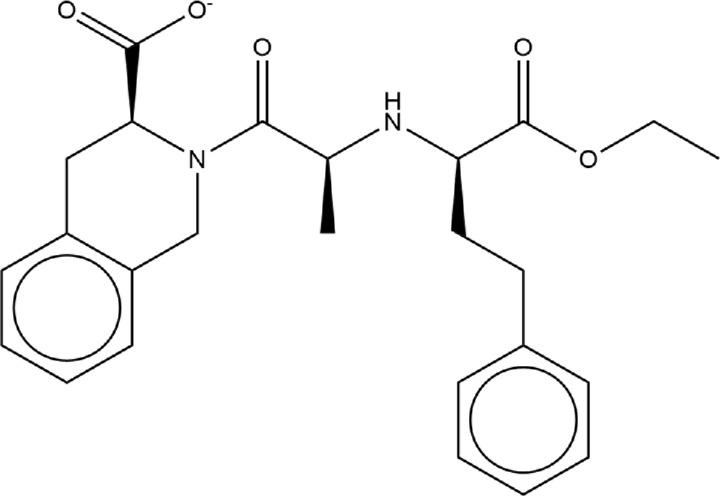
Captopril	Lisinopril	Ramipril
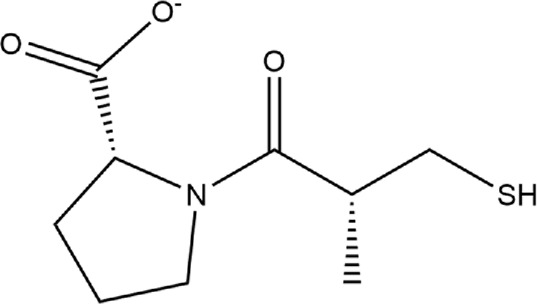	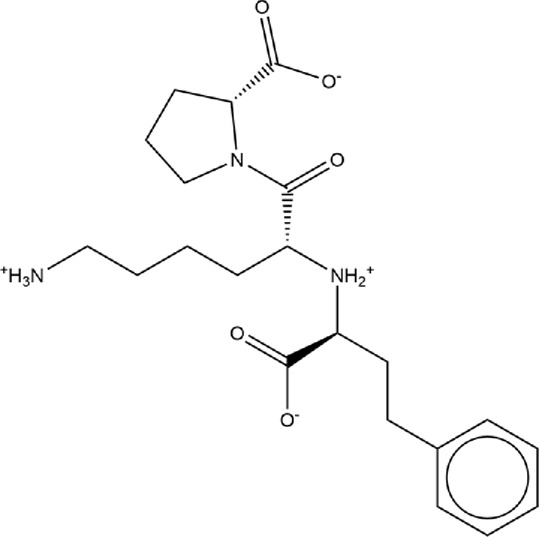	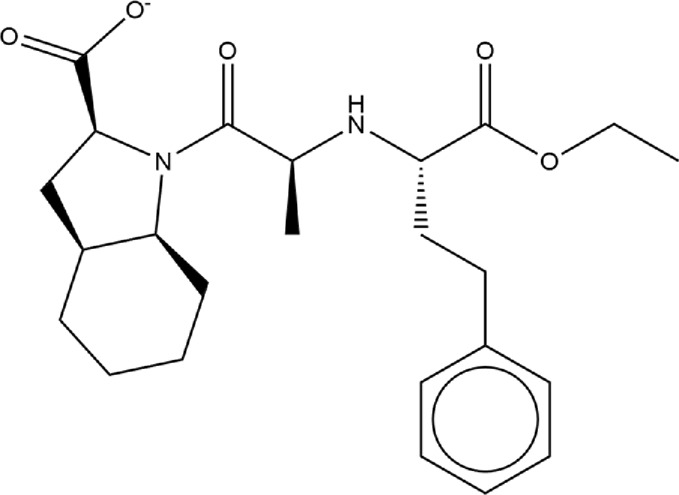
Enalapril	Lisinopril (−1)	Trandolapril
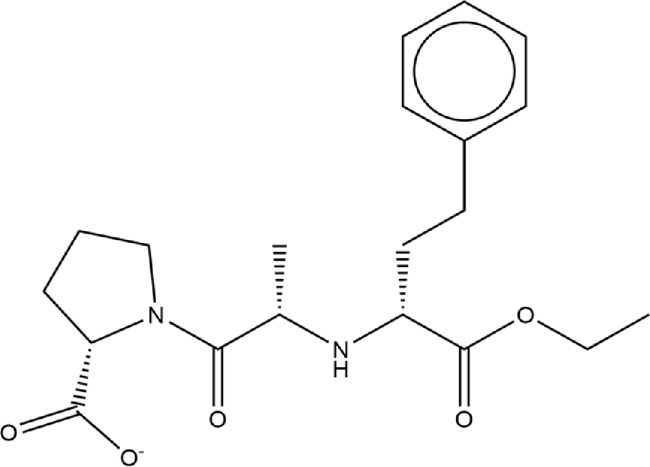	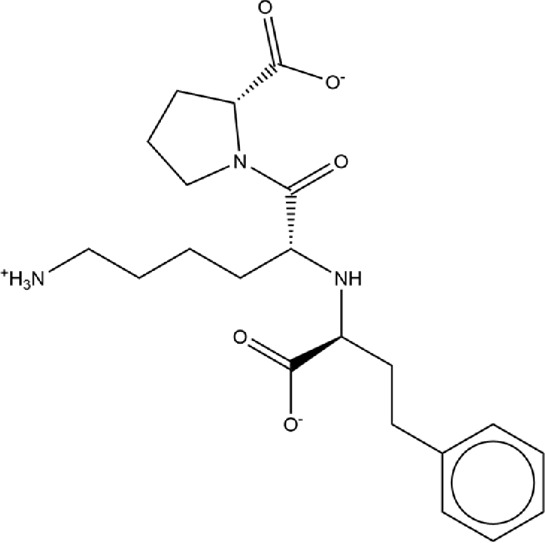	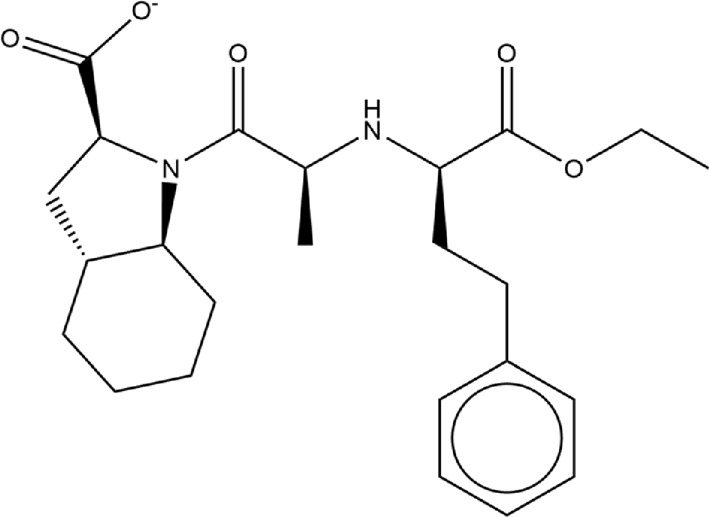
Fosinopril	Perindopril	
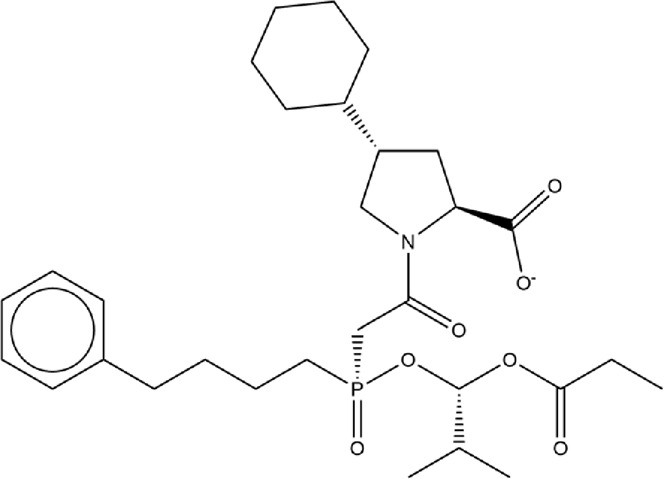	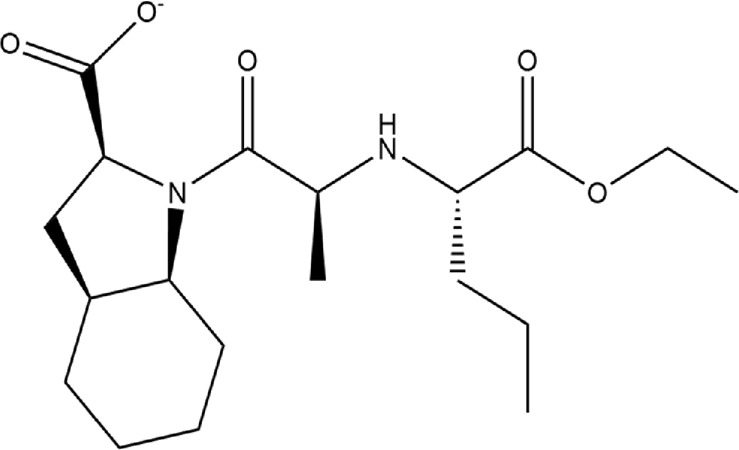	

**TABLE 2:** Structure of potential SARS-CoV-2 inhibitors.

Drug structure - potential SARS-CoV-2 inhibitors		
Aloe Emodin LS-H15204	Diquafosol	Emodin LS-H17409
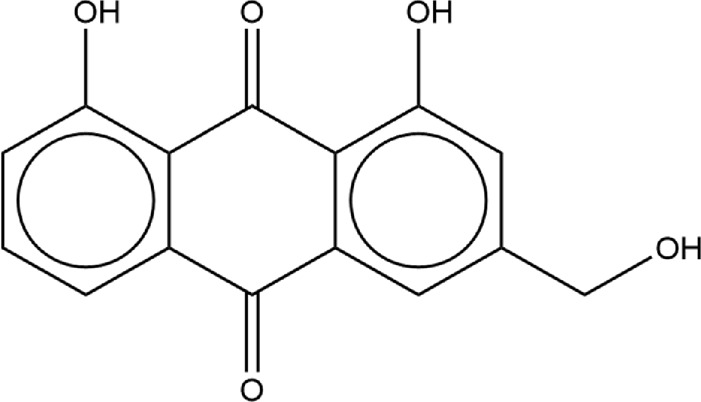	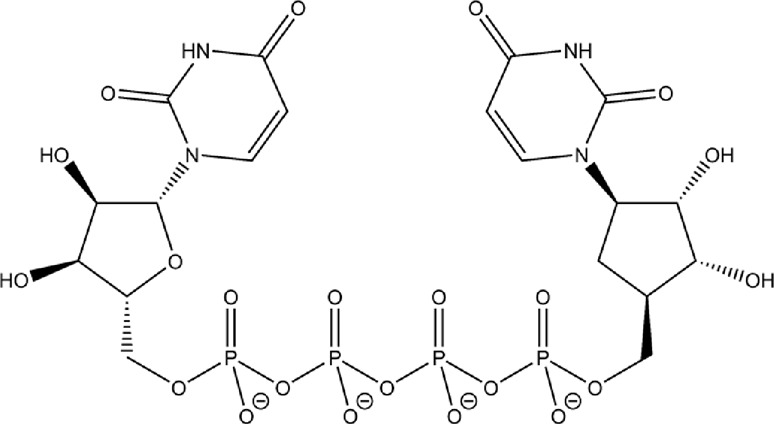	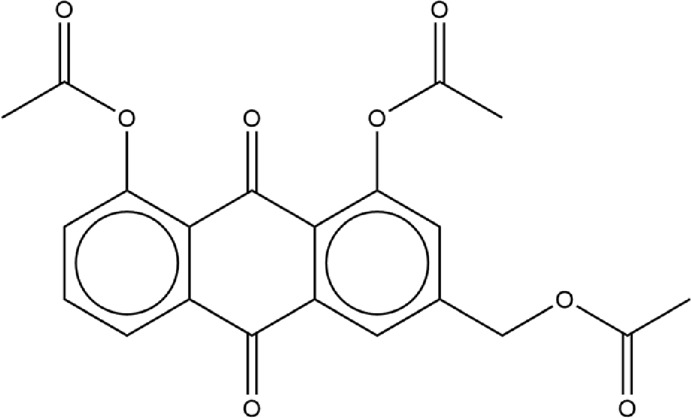
Camostat LS-H6976	Emodin LS-H11074	Physcion LS-H9395
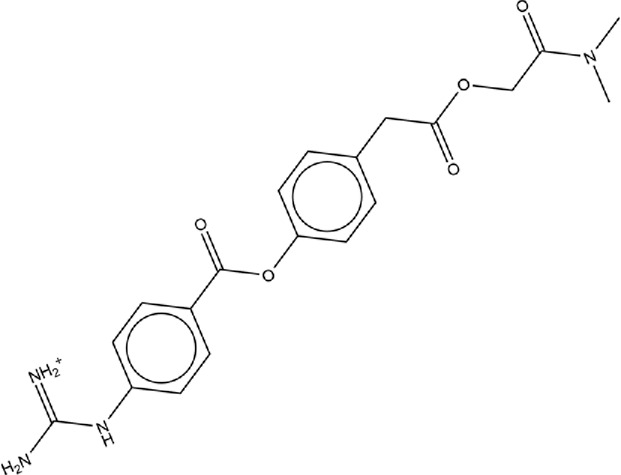	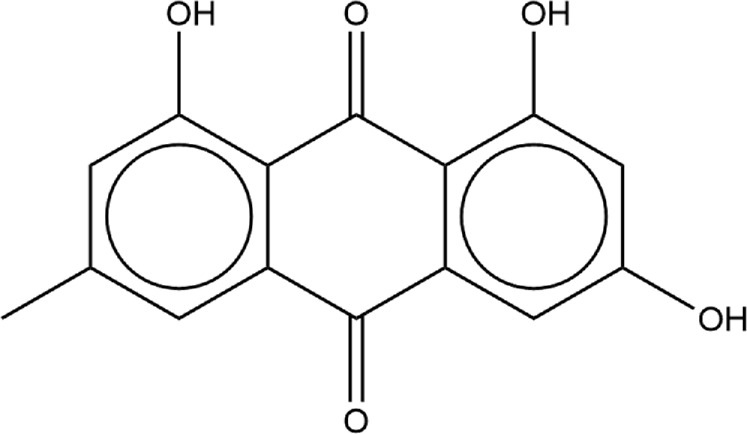	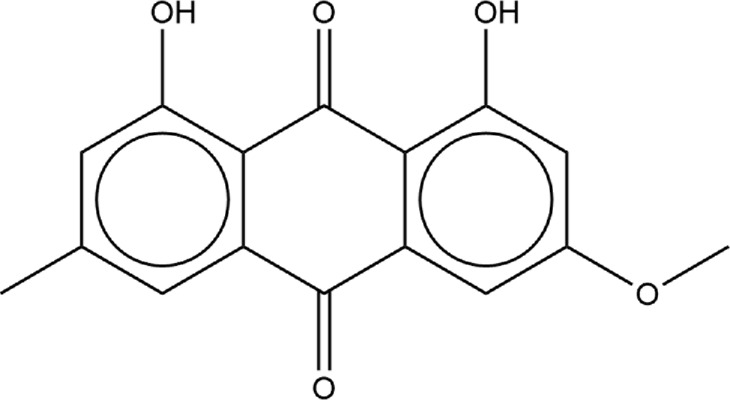

## Methods

### Protein retrieval and preparation

The hACE2 - SARS-CoV-2 RBD crystal structure was obtained from the Protein Data Bank (PDB Code 6LZG) (Berman et al., 2000; Wang et al., 2020c). Chains A (hACE2) and B (SARS-CoV-2 RBD) were selected from 6LZG. All waters were removed. Schrödinger’s Protein Preparation Wizard (Madhavi Sastry et al., 2013) was used to add missing hydrogen atoms, assign bond orders according to the Cambridge Crystallographic database (Groom et al., 2016), fill missing side chains using Prime (Jacobson et al., 2002; Jacobson et al., 2004), predict side chain protonation states using Schrödinger’s Empirical pKa Prediction (Epik) with a pH range of 7 ± 2, and optimize H-bonds using PROPKA at a pH of 7 (Olsson et al., 2011; Søndergaard et al., 2011; Schrödinger, 2019). Restrained minimization was performed using the OPLS3e force field and the default settings of the Protein Preparation Wizard (Harder et al., 2016).

### SiteMap analysis and receptor grid generation

Schrödinger’s SiteMap program was used to predict and score potential sites for ligand binding (Halgren, 2009). These potential sites are assigned both Site Scores (SScore) and Druggability Scores (DScore) based on volume, hydrophilicity/hydrophobicity, and hydrogen bonding ability. Binding sites with a SScore of at least 0.8 and a DScore of at least 0.83 are likely to favor ligand binding (Halgren, 2007; Halgren, 2009). Predicted binding sites are ranked based on size. SiteMap was run with default parameters on chains A and B of 6LZG. Five binding sites were identified (Supplementary Figure S4), three of which returned favorable scores for 6LZG. Site #2, which was identified as a potentially favorable binding site, occurs at the SARS-CoV-2 RBD - hACE2 complex junction. Schrödinger’s Receptor Grid Generation program (Friesner et al., 2006) was used to generate a 40 Å by 40 Å by 40 Å receptor grid in the x-, y- and z-directions, respectively, with a ligand size cutoff of 20 Å centered on Site #2 (Supplementary Figure S5). All parameters were kept at their default values. This receptor grid was used for all subsequent ligand docking.

### Ligand selection, preparation, and docking

Structures of common ACE inhibitors benazepril, captopril, enalapril, fosinopril, fosinoprilat, lisinopril, perindopril, quinapril, ramipril, and trandolapril were manually built and optimized according to the GAFF force field (Wang et al., 2004) using Avogadro 1.2 (Hanwell et al., 2012). Additionally, aloe emodin-LS-H15204, emodin-LS-H11074 and H17409, camostat-LS-H6976, and physcion-LS-H9395 were identified by LSBio as potential SARS-CoV-2 spike protein inhibitors and were selected for comparison to the ACE inhibitors (Yi et al., 2020). Diquafosol was selected due to its distinct structure and was identified as a potential SARS-CoV-2 inhibitor by previous studies (Hijikata et al., 2020; Sternberg et al., 2020). These drugs were also built using the same method detailed above. While this is not an expansive list, the goal of this study was to demonstrate that it is possible for a repurposed ligand to bind to the SARS-CoV-2 RBD - hACE2 interface. Additionally, we want to explore how ACE inhibitors act as SARS-CoV-2 RBD inhibitors and compare them to other identified small molecule drugs.

The Epik tool was used to predict the protonation states of each ligand at a pH of 7.4 ± 0.1 (Shelley et al., 2007; Greenwood et al., 2010; Schrödinger, 2023a). We used two different structures of lisinopril because, based on the predicted pKa, the protonation state at this pH was ambiguous (Tables 1, 2). The Schrödinger Glide Docking program (Friesner et al., 2004; Halgren et al., 2004; Friesner et al., 2006) was used to dock each ligand into the receptor grid centered in Site #2. Glide assigns a GlideScore to each ligand based on predicted polar and nonpolar interactions within the receptor grid (Friesner et al., 2004; Halgren et al., 2004). Default parameters were used with the XP docking algorithm to generate five poses per ligand. Ligands were selected for further analyses based on the GlideScore (Supplementary Tables S6, S7). The GlideScore cutoff was −4.5 kcal/mol; however, fosinoprilat was advanced for further study due to the structural similarity to fosinopril and the significant difference in GlideScores. Absorption, distribution, metabolism, and excretion (ADME) screenings were conducted on all ligands using Schrödinger’s QikProp ADME Program (Schrödinger, 2023b). Results were generated using the default settings and properties that exceeded the 95% range of known drugs are reported in the Supplementary Material. Additionally, pan-assay interference compounds (PAINS) screenings were conducted on the selected ligands using the following sources: https://www.cbligand.org/PAINS/ and https://zinc15.docking.org/patterns/home/. The default settings were used for both screenings.

### Molecular dynamics simulations

Unrestrained molecular dynamics (MD) simulations were conducted on the apo SARS-CoV-2 RBD - hACE2 complex and protein-ligand complexes using the AMBER 18 suite (Case et al., 2018). Top scoring ligands as identified from Glide were selected for further MD analysis along with fosinoprilat to diversify the range of binding affinities tested. The ff14SB (Maier et al., 2015) and Glycam06j (Kirschner et al., 2008) force fields were applied to the hACE2 and SARS-CoV-2 RBD glycoproteins. Previous studies support the significance of glycans to the RBD and hACE2 interface, so we ran all MD simulations with the N-glycosylated linkages maintained (Casalino et al., 2020; Acharya et al., 2021; Shajahan et al., 2021). The program *antechamber* was used to apply the GAFF force field and AM1-BCC charges to all ligands (Jakalian et al., 2000; Jakalian et al., 2002; Wang et al., 2004; Wang et al., 2006). All models were neutralized with Na^+^ ions and explicitly solvated in a TIP3P truncated octahedron using the program *tleap* (Jorgensen and Madura, 1983). The Na^+^ and hACE2 active site Zn^2+^ atoms were modeled using TIP3P ion parameters. In the experimental structure, the zinc ion interacts with residues His374, Glu375, His378, and Glu402 in the hACE2 active site. Trajectory analysis confirmed that the ion remained complexed to these residues throughout the simulations. All simulations were performed using the GPU-accelerated *pmemd* code of AMBER18 (Götz et al., 2012; Salomon-Ferrer et al., 2013). A process of minimization, heating, and equilibration was performed prior to running unrestrained MD. Minimization occurred in seven stages of a maximum of 5000 steps each. The first 1,000 steps consisted of the steepest descent minimization, and the remaining 4,000 steps consisted of conjugate gradient minimization. An initial restraining force of 10.0 kcal/mol Å^-2^ applied to the heavy atoms of the solute was methodically decreased over the seven stages to 5.0, 2.0, 1.0, 0.5, 0.1, and lastly, 0.0 kcal/mol Å^-2^. Each structure was then heated linearly from 10 to 300 K while a restraining force of 10.0 kcal/mol Å^-2^ was reapplied to all heavy atoms. Equilibration was then conducted in seven 500 ps stages, with the initial restraining force methodically decreased to 0.0 kcal/mol Å^-2^ following the same procedure as that of minimization. Completion of equilibration was followed by ten randomly selected seeds (initial atomic velocities) of unrestrained MD for 300 ns of the apo complex or five seeds of unrestrained MD for 100 ns of the ligand-bound complexes. It is important to note that we are using MD simulation to sample the local environment of various low energy structures but not necessarily expecting to see large conformational interconversions. Subsequently, simulations were initiated from various docked poses in order to better sample the full potential energy surface. Trajectory visualization and imaging were conducted using UCSF Chimera and UCSF ChimeraX (Pettersen et al., 2004; Goddard et al., 2018). All analyses were conducted using the AMBER 20 suite (Case et al., 2020). Root-mean-squared deviation (RMSD), root-mean square fluctuation (RMSF), center of mass, hydrogen bonding analysis, and backbone atom RMSD-based clustering were conducted using the AmberTools *cpptraj* module (Roe and Cheatham, 2013).

MM-GBSA binding free energy and per-residue decomposition analyses were conducted on all frames for each simulation using the AmberTools *MMPBSA. py* package, with pairwise decomposition analysis conducted on every 10th frame (Onufriev et al., 2004; Miller et al., 2012). The GB^OBC2^ model (igb = 5) was used for the previously mentioned analyses. We elected to perform MM-GBSA rather than MM-PBSA analysis because these two methods are known to produce comparable results, especially when estimating free energies for similar systems, i.e., a protein-ligand complex where only the ligand changes (Sun et al., 2014; Genheden and Ryde, 2015; Wang et al., 2019). MM-PBSA is an order of magnitude more computationally demanding and given the large size of the hACE2-RBD-ligand complex, we felt that MM-GBSA was a sufficient level of theory to establish a baseline level of affinity for these ligands with the RBD-hACE2 binary complex.

Native contact analysis, as defined by Best et al. (2013), was conducted on all trajectories between RBD and hACE2 heavy atom pairs. MDTraj was used to calculate the native contacts in the interface (McGibbon et al., 2015). The percentage of time a ligand spent dissociated from the binding site was also determined using MM-GBSA and center of mass analyses. The average MM-GBSA value representing the binding free energy between the ligand and the RBD - hACE2 complex of each frame was used to determine the frames where the drug was not interacting with any residues of the complex. And the center of mass distance between the initial position of the ligand and every frame of the simulation was used to determine the frames where the drug was no longer present in the interface. Specifically, if the distance was greater than 10 Å. The frames identified from both analyses were used to determine the percent dissociation of each ligand selected for MD simulations.

## Results and discussion

### Apo MD

Our goal was to analyze the residue interactions significant for binding of the SARS-CoV-2 RBD with the hACE2 receptor, and to determine whether repurposed drugs might disrupt this binding. We performed 300 ns of unrestrained MD simulations using ten different seeds to understand the baseline energetics of the SARS-CoV-2 RBD - hACE2 interaction. The average MM-GBSA binding free energy over the 3 µs trajectory was −31.23 kcal/mol with a std. deviation and std. error of 10.55 and 0.06 kcal/mol, respectively. MM-GBSA interaction energies for each seed are shown in Supplementary Figure S1; Supplementary Table S1.

**FIGURE 1:**
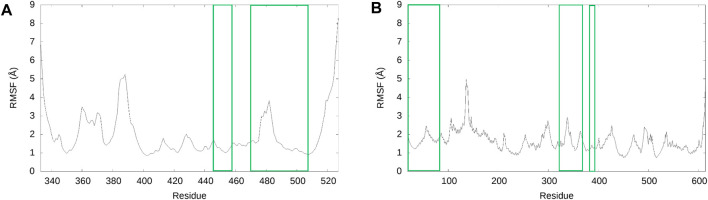
RMSF of SARS-CoV-2 RBD and hACE2 structures for the 3 µs ensemble. **(A)** The RMSF of the RBD. **(B)** The RMSF of hACE2. For both graphs, the residues of the RMSF data enclosed in green are residues involved in the interface of the SARS-CoV-2 RBD–hACE2 interaction, according to Wang et al. (2020c).

To confirm the structural stability of the SARS-CoV-2 RBD - hACE2 apo complex, we analyzed the RMSD over the 3 µs trajectory relative to the initial structure (Supplementary Figure S2). To understand the conformational dynamics of the individual amino acid residues in the apo structure, we calculated the RMSF of the RBD and hACE2 residues. Figure 1 displays the RMSF for the RBD (1A, residues 333–527) and hACE2 (1B, residues 19–614). Residues 455–490 in the receptor binding motif (RBM) of the receptor binding domain (RBD) have been experimentally determined to be intrinsically disordered (Quaglia et al., 2022). The RMSF values in Figure 1A suggests that in our simulations the conformational dynamics for residues between 455 and 475 are minor, i.e., this is a region that is conformationally stable. We do see an increase in residue fluctuations between 475 and 490 but even here the excursions are less than 4Å. A comparison of this data with the apo secondary structure plots discussed below suggest that the region is somewhat disordered, containing mostly bend and turn motifs and is devoid of helical structure. Residues 485 through 490 form a parallel beta sheet in both the apo and hACE2 bound form. In Figure 1, residues involved in the SARS-CoV-2 RBD and hACE2 interface are highlighted with green boxes. Notably, the residues involved in the interface are stable with RMSF values ranging from 1 to 3 Å. In Figure 1A, there is a peak in RMSF for the residues 384–390 of the RBD. In a previous study of the WT and Omicron SARS-CoV-2 RBD–hACE2, we found the same increase in fluctuation of the residues 384–390 in the WT RBD was caused by the absence of a 3-10 helix involving these residues. (Carter et al., 2023). Visualization of the apo MD trajectories in this study confirms the lack of secondary structure for this range of residues during the 3 µs simulation.

Pairwise decomposition and hydrogen bonding analyses were used to identify residue interactions that are significant for the interaction between the SARS-CoV-2 RBD and hACE2. Supplementary Table S2 demonstrates residue interactions that are significant based on pairwise decomposition energies (below −2 kcal/mol). Notably, there are no residue interactions with significant repulsion (above 0.5 kcal/mol). Additionally, Supplementary Table S3 details the hydrogen bond occurrences (above 5%) of molecular interactions. The average occurrences shown in Supplementary Table S3 were calculated over the 3 µs ensemble. RBD Tyr505 – hACE2 Lys353, RBD Asn501 – hACE2 Lys353, RBD Phe486 – hACE2 Met82, RBD Asn501 – hACE2 Tyr41, and RBD Gln493 – hACE2 His34 are interactions that have significant pairwise decomposition energies not driven by hydrogen-bonding, i.e., they have insignificant or no hydrogen bond occurrences. These residue interactions are driven by favorable electrostatic and/or van der Waals interactions (Supplementary Table S2). Residue interactions that are driven by hydrogen bonding are shown in Supplementary Table S4, along with their pairwise decomposition energies. RBD Tyr505 – hACE2 Ala386, RBD Ala475 – hACE2 Ser19, RBD Gly446 – hACE2 Gln42, and RBD Tyr495 – hACE2 Lys353 interactions from Supplementary Table S4 have low pairwise decomposition energies; however, this can be explained by the relatively low percent occurrence of hydrogen bonding. While these particular residues are not dominating the RBD–hACE2 interaction, they will become disrupted upon ligand binding, as described below and in the Supplementary Material.

Previous studies have identified SARS-CoV-2 RBD residues that are significant for RBD–hACE2 binding. Yi et al. (2020) determined that single amino acid substitution on residues Leu455, Phe456, Ser459, Gln474, Ala475, Phe486, Phe490, Gln493, and Pro499 reduced binding affinity for hACE2. Our results are in agreement with this as we found that residues Ala475, Phe486, and Gln493 participate in notable interactions with hACE2 residues (Supplementary Tables S2–S4). Lan et al. identified important RBD–hACE2 interactions involving the RBD residues Lys417, Leu455, Phe486, Gln493, and Asn501. (Lan et al., 2020). This is consistent with our analysis as we find the following interactions to be significant: RBD Phe486 – hACE2 Met82, RBD Gln493 - hACE2 Lys31, His34, and Glu35, RBD Asn501 – hACE2 Tyr41, and RBD Lys417 – hACE2 Asp30.

**FIGURE 2:**
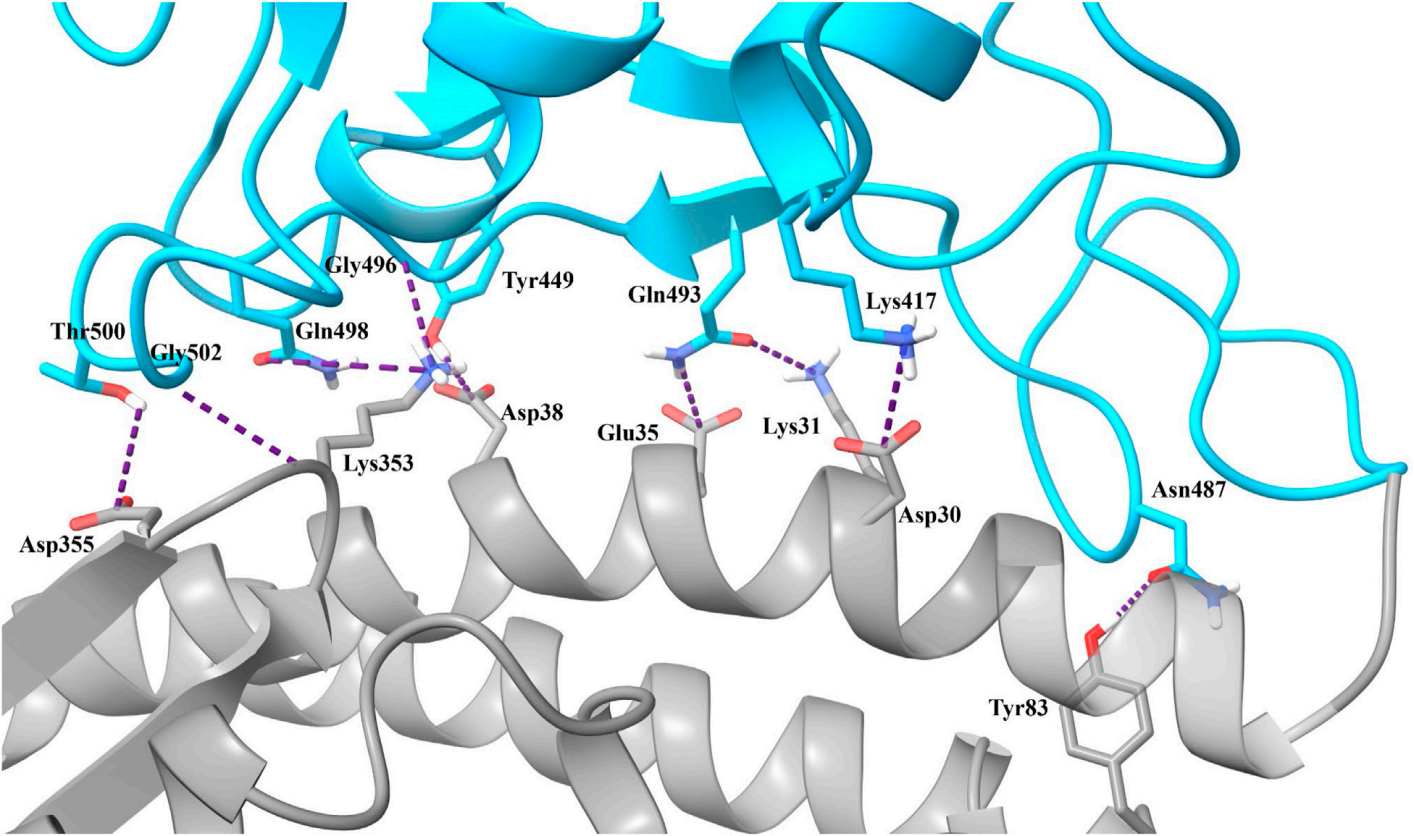
Significant interactions for the SARS-CoV-2 RBD–hACE2 complex. Interactions detailed in Table 3 are displayed with hydrogen bonds depicted in purple.

Based on our analysis, the most important residue interactions in the apo structure are detailed in Table 3 and depicted in Figure 2. Specifically, in Figure 2, the amino acids involved in the interactions listed in Table 3 are labeled, and the corresponding side chains are illustrated. Notably, Lys417 is the only RBD residue involved in a significant interface interaction that is not present in the RBM.

**TABLE 3:** Significant SARS-CoV-2 RBD–hACE2 interactions. Hydrogen bonding occurrences for residue interactions between the SARS-CoV-2 RBD and the hACE2 receptor and the corresponding pairwise decomposition energy are listed. The table includes interactions with a hydrogen bond occurrence greater than 50% or a pairwise decomposition energy less than −3.00 kcal/mol.

RBD residue	hACE2 residue	Hydrogen bonding percent occurrence (avg.)	Pairwise decomposition (avg. (std. error)) (kcal/mol)
Asn487	Tyr83	69.56	−2.89 (0.02)
Gly502	Lys353	66.80	−1.63 (0.006)
Tyr449	Asp38	59.72	−3.59 (0.04)
Lys417	Asp30	57.81	−6.52 (0.05)
Gln493	Glu35	52.44	−4.50 (0.03)
	Lys31	36.74	−4.13 (0.04)
Thr500	Asp355	42.19	−4.70 (0.04)
Gln498	Lys353	34.90	−3.86 (0.06)
Gly496	Lys353	26.47	−3.25 (0.03)

Additionally, we ran native contact analysis using the concatenated 3 μs ensemble compared to the initial structure obtained after minimization of the 6LZG experimental structure. We identified 299 native contacts in the interface between SARS-CoV-2 and hACE2. Supplementary Figure S3 displays the fraction of native contacts for each frame throughout the simulation with an average of 0.94 ± 0.03 (std. deviation).

### SiteMap

**FIGURE 3:**
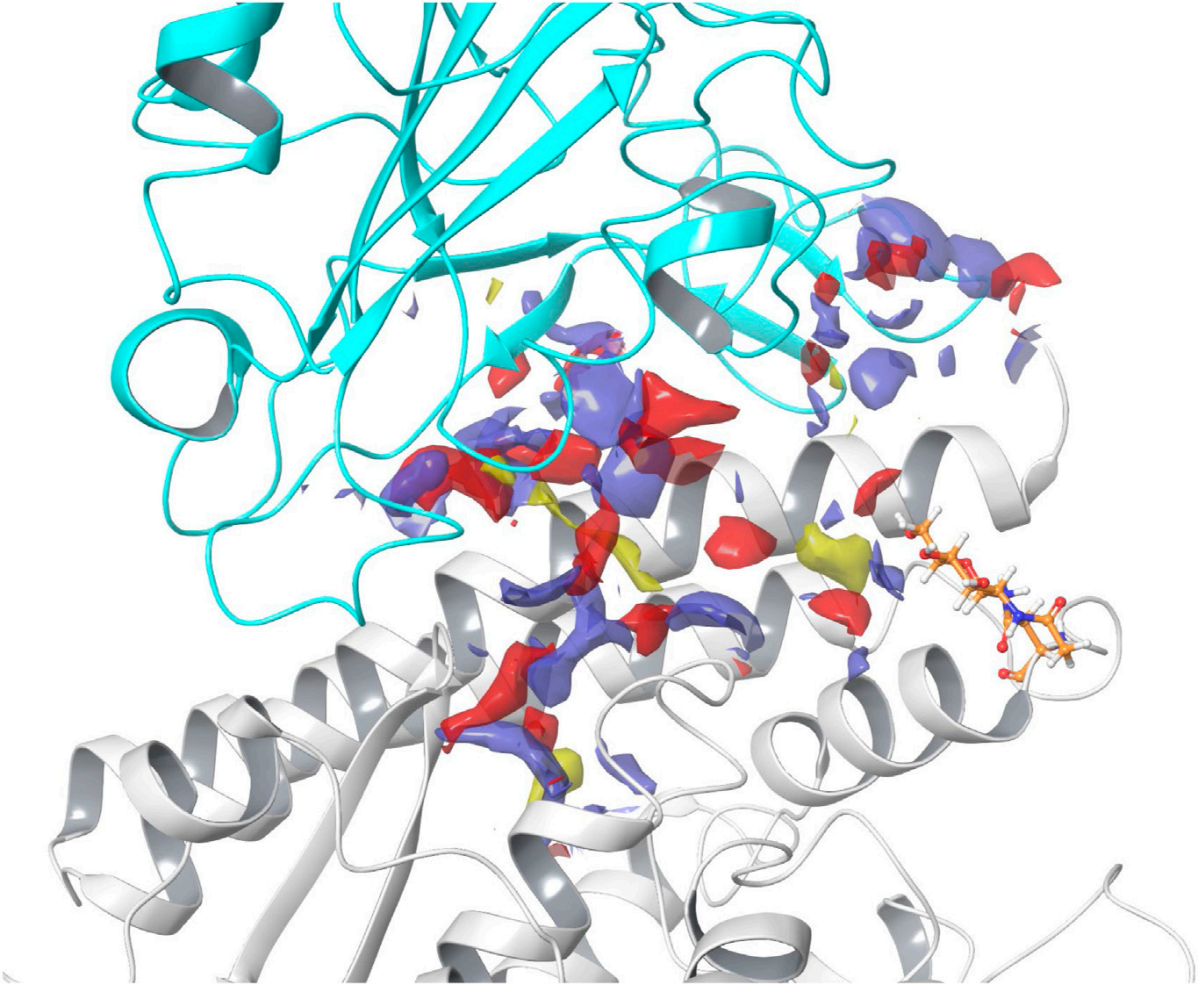
SARS-CoV-2 RBD - hACE2 complex junction binding site. In the image, hACE2 is shown in gray and SARS-CoV-2 RBD is shown in cyan. Hydrogen bond acceptor sites are colored red, hydrogen bond donating sites are colored purple, and hydrophobic sites are colored yellow. The N-linked glycan bound to Asn90 is shown in orange. Site #2 on the 6LZG complex was predicted to have a SiteMap SScore of 1.002 and DScore of 1.017.

SiteMap was used to predict and score potential ligand binding sites based on volume, hydrophilicity/hydrophobicity, and hydrogen bonding ability (Halgren, 2009). Five binding sites were identified, with three displaying favorable SScores and DScores. Sites #1 and #3 appear to flank the ACE2 active site (Guy et al., 2003; Guy et al., 2005). Site #2, displayed in Figure 3 (SScore 1.002, DScore 1.017), occurs at the SARS-CoV-2 RBD - hACE2 complex junction. This site notably contains many residues necessary for the formation of this complex (Wang et al., 2020c). This suggests that it is possible for a ligand to disrupt the binding of the SARS-CoV-2 RBD with hACE2 and therefore Site #2 was the site selected for ligand docking. Two additional smaller binding sites (Sites #4 and #5) are predicted on the surface of hACE2. All sites and their properties are detailed in Supplementary Figure S4; Supplementary Table S5.

### Epik and glide docking

To determine whether a ligand might favorably bind to Site #2, a small sample of common ACE inhibitors, molecules identified by LSBio as potential SARS-CoV-2 spike protein inhibitors, and diquafosol, were screened using Glide (Friesner et al., 2004; Halgren et al., 2004). In preparation for docking, we determined the proper protonation states of all ligands at pH = 7.4 using Epik (Shelley et al., 2007; Greenwood et al., 2010; Schrödinger, 2023a). Epik predicted the backbone amine of lisinopril to be protonated (-NH_2_^+^-) but given its location in the backbone we also performed calculations with the (-NH-) form of lisinopril (Table 1.) The receptor grid used for ligand docking is illustrated in Supplementary Figure S5. All structures, XP GlideScores, and their relative ranking are detailed in Supplementary Tables S6–S8.

Of the 17 ligands screened, seven ligands and their three top scoring poses were selected for further analysis. Notably, the top five ranked ligands consisted mostly of potential SARS-CoV-2 spike protein inhibitors as suggested on the LSBio database and previous studies (Hijikata et al., 2020; Sternberg et al., 2020; Yi et al., 2020). Diquafosol produced the highest scoring poses of all ligands investigated. Fosinopril, lisinopril, physcion, and emodin_H11074 (emodin) all produced high ranking poses. Lisinopril, with an overall charge of −1, was ranked 15, but was selected for further analysis because we were interested in how the protonation state affected its binding to the interface. Also, while fosinoprilat was not a top ranked drug based on docking results, it is a prodrug of fosinopril and has a very similar structure. It was selected for further study for comparison to fosinopril. For all ligands selected for further study, we initiated MD simulations using poses that were structurally distinct.

### ADME and PAINS screening

Before beginning MD simulations, we assessed the pharmacological properties of each ligand using the Schrödinger QikProp ADME program (Supplementary Table S9) (Schrödinger, 2023b). All ligands selected for MD analysis are known drugs in clinical use, so we expected favorable ADME properties. The properties of all ligands were within the 95% range of known drugs except for diquafosol, details of which are described in the Supplementary Material. Additionally, PAINS screenings were conducted on the seven ligands advanced for MD study based on their Glide scores. Emodin and physcion did not pass PAINS screening because of the quinone core in both structures. Also, both compounds are known to have numerous pharmacological properties, such as anti-cancer, anti-inflammatory, and antimicrobial activity, which further supports the non-specific activity of these compounds. (Dong et al., 2016; Pang et al., 2016). All other ligands passed PAINS screening, suggesting the propensity for binding specificity. Overall, from ADME analysis and PAINS screenings we concluded that these seven ligands are viable compounds for further analysis.

### Ligand-bound MD simulations

**FIGURE 4:**
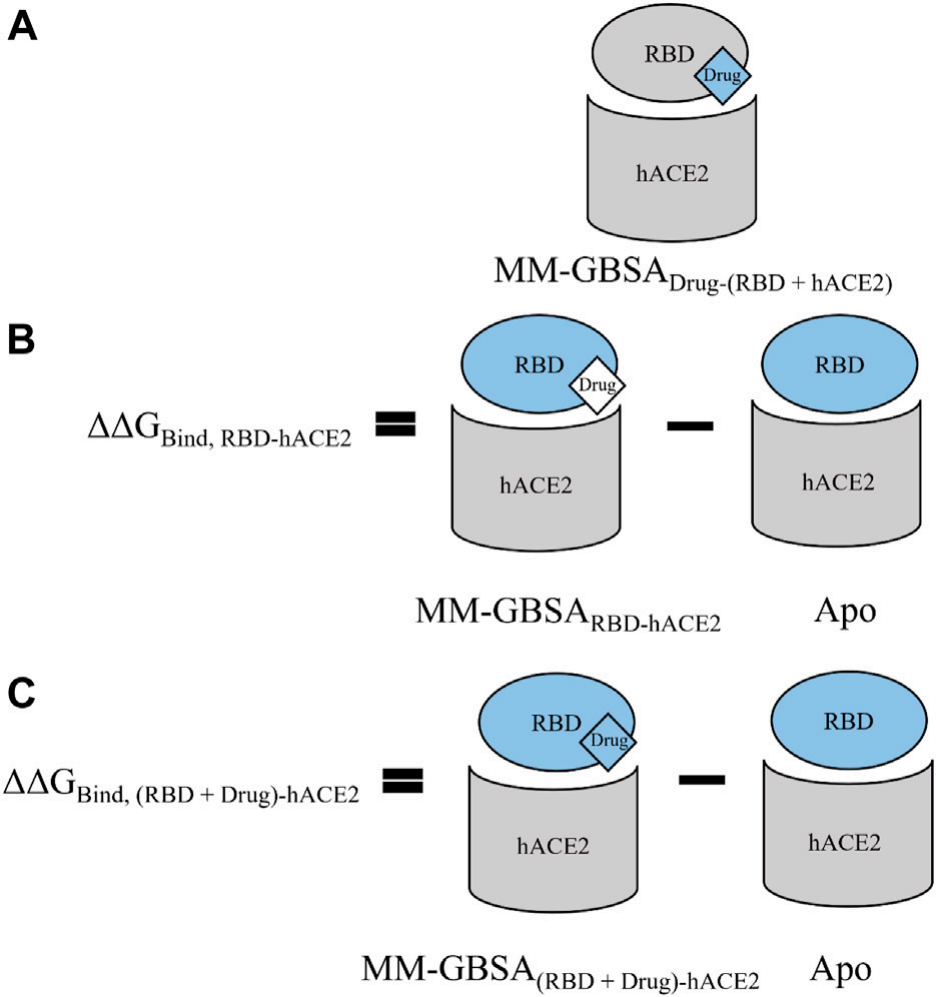
A visual representation of the various MM-GBSA energies that we used to evaluate binding. For all panels, the receptor defined for MM-GBSA calculations is in gray, and the defined ligand is in blue. **(A)** For the MM-GBSA_Drug-(RBD+hACE2)_ calculation, the RBD and hACE2 are taken together and treated as the receptor while each drug is treated as the ligand; therefore, the binding free energy is calculated between the drug and the RBD–hACE2 complex. **(B)** MM-GBSA_RBD-hACE2_ is the binding free energy between the RBD and hACE2 with the ligand bound in the interface–though the drug is excluded from the calculation (shown in white)–it affects the conformations and interactions of RBD and hACE2. We subtract this value from the apo binding free energy between the RBD and hACE2, i.e., when no drug is bound. The average value of this apo energy is −31.23 (0.06) kcal/mol for the 3 μs ensemble. This difference is ΔΔG_Bind, RBD-hACE2_, which tells us how the drug impacts the binding of RBD to hACE2. A positive ΔΔG_Bind, RBD-hACE2_ signifies that the drug inhibited the interaction between the RBD and hACE2. **(C)** MM-GBSA_(RBD+Drug)-hACE2_ represents the average binding free energy between hACE2 and the RBD–drug complex. The difference between MM-GBSA_(RBD+Drug)-hACE2_ and apo is ΔΔG_Bind, (RBD+Drug)-hACE2_, which signifies the drug’s effects on the RBD–hACE2 interaction after the drug binds first to the RBD. Overall, ΔΔG_Bind, (RBD+Drug)-hACE2_ represents whether hACE2 prefers to bind to the drug-bound RBD or the apo RBD. A negative value signifies that the interaction between the drug-bound RBD and hACE2 is stronger than the interaction between apo RBD and hACE2. This suggests that the trimeric complex is more stable than the RBD–hACE2 apo complex. All values can be found in Table 4.

Unrestrained MD simulations were conducted on SARS-CoV-2 RBD–hACE2 complexes (6LZG) bound to either diquafosol, emodin, fosinopril, fosinoprilat, lisinopril with both protonation states, or physcion. For each ligand, various docked poses were used to initiate 5 seeds of 100 ns simulations. Initiating simulations from different starting structures allows for better potential energy surface sampling, as we are utilizing MD to sample the local environment of low energy structures and not necessarily expecting to see large conformational interconversions. RMSD (Supplementary Table S10) was used to understand the structural stability of the SARS-CoV-2 RBD and hACE2 complex with a drug present in the interface. For all simulations, other than physcion seeds 3 and 4 where it was visually confirmed that the ligand is moving away from the interface, the ternary trajectories are structurally stable. For all ligand-bound 500 ns ensembles, a variety of MM-GBSA binding free energies were calculated in order to probe the effect of ligand binding to the RBD–hACE2 interface (Table 4). Figure 4 depicts how each MM-GBSA energy was calculated.

**TABLE 4:** MM-GBSA and ΔΔG_Bind_ values.

Ligand	Pose	A. MM-GBSA_Drug-(RBD+hACE2)_ Avg. (Std. Error)	Percent dissociation	B.1. MM-GBSA_RBD-hACE2_ Avg. (Std. Error)	B.2. ΔΔG_Bind, RBD-hACE2_	C.1. MM-GBSA_(RBD+Drug)-hACE2_ Avg. (Std. Error)	C.2. ΔΔG_Bind, (RBD+Drug)-hACE2_
Diquafosol	1	−14.86 (0.23)	2.70	−26.65 (0.13)	4.58	−51.52 (0.19)	−20.29
	3	−5.60 (0.33)	9.66	−25.01 (0.17)	6.22	−27.31 (0.21)	3.92
Emodin	1	−10.98 (0.07)	33.32	−22.93 (0.14)	8.30	−33.33 (0.14)	−2.10
	2	−14.94 (0.08)	4.22	−27.13 (0.14)	4.10	−41.90 (0.16)	−10.67
Fosinopril	1	−24.51 (0.15)	11.60	−25.48 (0.13)	5.75	−39.40 (0.16)	−8.17
Fosinoprilat	1	−18.30 (0.12)	43.46	−20.92 (0.13)	10.31	−33.47 (0.18)	−2.24
	2	−29.01 (0.16)	0.06	−30.04 (0.14)	1.19	−44.68 (0.16)	−13.45
	3	−21.13 (0.18)	2.04	−30.19 (0.14)	1.04	−43.35 (0.19)	−12.12
Lisinopril (NH_2_^+^)	1	−35.05 (0.14)	0.26	−30.26 (0.13)	0.97	−53.72 (0.23)	−22.49
Lisinopril (NH)	1	−31.80 (0.16)	6.74	−29.78 (0.14)	1.45	−56.41 (0.20)	−25.18
Physcion	1	−10.37 (0.07)	82.46	−24.94 (0.15)	6.29	−31.72 (0.17)	−0.49
	2	−12.02 (0.11)	72.26	−23.86 (0.14)	7.37	−32.82 (0.17)	−1.59
	3	−10.98 (0.09)	77.70	−34.05 (0.14)	−2.82	−40.04 (0.18)	−8.81

**A.** MM-GBSA_Drug-(RBD+hACE2)_ is the average binding free energy (kcal/mol) between the drug and the RBD - hACE2 complex. For a visual representation of MM-GBSA_Drug-(RBD+hACE2)_ refer to Figure 4A. Additionally, the percent dissociation is reported for each ligand and the corresponding pose. Percent Dissociation was calculated using MM-GBSA values, center of mass analysis, and visualization. More details are provided in the methods section **B.1.** MM-GBSA_RBD-hACE2_ is the average binding free energy (kcal/mol) between the RBD and hACE2 while a drug is present in the interface. For a visual representation of MM-GBSA_RBD-hACE2_ refer to Figure 4B. **B.2.** Apo is the average binding free energy (kcal/mol) between the RBD and hACE2 [−31.23 (0.06) kcal/mol for the 3 μs ensemble], which was used to calculate the ΔΔG_Bind, RBD-hACE2_ values. ΔΔG_Bind, RBD-hACE2_ signifies the effect the drug has on the RBD–hACE2 interaction, with positive values signifying drug inhibition of the interaction. **C.1.** MM-GBSA_(RBD+Drug)-hACE2_ is the average binding free energy (kcal/mol) of the drug-bound RBD and hACE2. For a visual representation of MM-GBSA_(RBD+Drug)-hACE2_ refer to Figure 4C. **C.2.** The apo MM-GBSA value was used to calculate the ΔΔG_Bind, (RBD+Drug)-hACE2_ values (kcal/mol), which represent the effect of the drug on the interaction between hACE2 and the RBD, after the RBD binds first with the drug.

Table 4, Column A indicates the average binding free energies of the ligands interacting in the interface of the RBD - hACE2 complex, MM-GBSA_Drug-(RBD+hACE2)_. Figure 4A is a visual representation of MM-GBSA_Drug-(RBD +hACE2)_. This energy represents how tightly the ligand binds to the dimer complex. We were interested in determining this binding to understand if it is feasible for the ligands to inhibit the interaction between the RBD and hACE2. The MM-GBSA_Drug-(RBD+hACE2)_ values for all ligand-bound complexes are negative, signifying that the drug participates in favorable interactions in the interface. Lisinopril (protonation states -NH- and -NH_2_^+^-), fosinoprilat, and fosinopril have the strongest interactions. We also determined the percent dissociation (Table 4) of the drug from the RBD - hACE2 interface over the concatenated 500 ns simulation. Notably, a number of simulations initiated from different poses of diquafosol, emodin, fosinopril, fosinoprilat, and lisinopril (both protonation states -NH- and -NH_2_^+^-) have relatively low percent dissociations. The percent dissociation for all physcion trajectories indicates that the ligand is not in the binding site for more than half of the simulation.

In order to determine the degree of inhibition caused by each drug binding to the interface, we calculated the MM-GBSA average binding free energies between the RBD and hACE2 while a drug is present in the interface, MM-GBSA_RBD-hACE2_ (Table 4, Column B.1.). The difference between the MM-GBSA_RBD-hACE2_ values and the binding free energy of the apo 3 μs ensemble, −31.23 (0.06) kcal/mol, (MM-GBSA_RBD-hACE2_–apo) represents the drug’s effect on the RBD - hACE2 interaction (Figure 4B). This difference is referred to as ΔΔG_Bind, RBD-hACE2_ and a positive value suggests that the presence of the drug destabilizes the RBD–hACE2 interaction (Table 4, Column B.2.). Each ligand-bound complex shows a positive, inhibitory ΔΔG_Bind, RBD-hACE2_ value, except for physcion pose 3. While these positive values speak to the promise of these ligands as RBD–hACE2 binding disruptors, we note that the some of the MM-GBSA_RBD-hACE2_ values are not significantly different from the apo binding free energy (−31.23 (0.06) kcal/mol), suggesting that the ligand effect on the interaction between the RBD and hACE2 is small.

We also explored the energetics of a ligand-bound RBD interacting with hACE2, i.e., binding the ligand to RBD first, and then having that binary complex interact with hACE2. These values are MM-GBSA_(RBD+Drug)-hACE2_ (Table 4, Column C.1.). The difference between MM-GBSA_(RBD+Drug)-hACE2_ and the binding free energy of the apo 3 μs ensemble [−31.23 (0.06) kcal/mol], ΔΔG_Bind, (RBD+Drug)-hACE2_, represents the impact the drug has on the interaction between the RBD and hACE2, *after the drug binds first to the RBD* (Figure 4C). Another way to interpret this value is whether hACE2 prefers to bind to the drug-bound RBD or the apo RBD. A positive or near zero value of ΔΔG_Bind, (RBD+Drug)-hACE2_ suggests that the binding of the drug to RBD may disrupt the RBD–hACE2 interaction (i.e., hACE2 favors binding to apo RBD over the drug-bound RBD). The ΔΔG_Bind, (RBD+Drug)-hACE2_ energies suggest that the ligand-bound RBD interacts more favorably with hACE2, although in seven cases the change in binding is minimal, i.e., the absolute value of the ΔΔG_Bind, (RBD+Drug)-hACE2_ is less than the std. dev. of the apo MM-GBSA (10.55 kcal/mol) (Table 4, Column C.2.). For the following ligands there is a significant difference between the MM-GBSA_(RBD+Drug)-hACE2_ energies and the apo binding free energy: diquafosol pose 1, emodin pose 2, fosinoprilat poses 2 and 3, and lisinopril (both protonation states -NH- and -NH_2_^+^-) pose 1. This result suggests that the RBD bound to these ligands more readily binds to hACE2 than the RBD on its own. This suggests that these ligands are not viable candidates for disrupting the RBD–hACE2 interface; indeed, the presence of the ligand stabilizes the trimeric complex more so than the apo complex. These results underscore the importance of studying the disruption of the RBD - hACE2 interface and not just drug binding to the RBD, i.e., drugs that show inhibitory properties to one of the proteins (RBD) can increase the affinity for that viral protein to bind with the human target.

The MM-GBSA and percent dissociation analyses suggest that all of the ligands (except physcion) can bind to the RBD - hACE2 interface, but that the RBD and hACE2 still interact favorably even in the presence of some of the ligands. ΔΔG_Bind, (RBD+Drug)-hACE2_ analysis suggest that the ligand binding to the RBD enhances the tertiary interaction with hACE2, especially for diquafosol, fosinoprilat and both forms of lisinopril. To better understand these binding interactions, we explored the individual amino acid interactions between the RBD, hACE2, and each drug to further understand how the ligands affect the RBD - hACE2 interaction.

**FIGURE 5:**
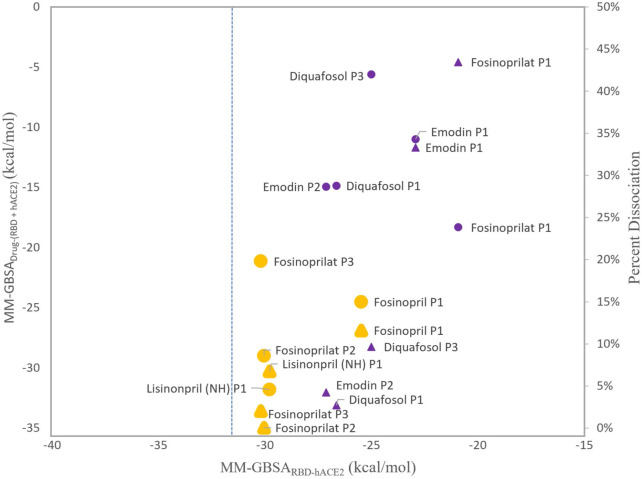
Comparison of MM-GBSA energies and percent dissociation. The x-axis represents the average MM-GBSA binding free energies between the RBD and hACE2 with a ligand present in the interface, MM-GBSA_RBD-hACE2_. The y-axis (left side) represents the MM-GBSA binding free energies of the ligand interacting with the RBD–hACE2 complex, MM-GBSA_Drug-(RBD+hACE2)_. The MM-GBSA_Drug-(RBD+hACE2)_ energies with respect to the x-axis are represented as circles. The y-axis (right side) represents the percent dissociations. These values with respect to the x-axis are triangles. The larger symbols depicted in orange represent ligands that were selected for further analyses. All other symbols are purple. All ligands, except physcion, are included and labeled next to each data point. Physcion was not included because it appeared to be an outlier once the data was visualized. The blue dashed line represents the apo MM-GBSA binding free energy, −31.23 (0.06) kcal/mol, in comparison to the MM-GBSA_RBD-hACE2_ values.

To decide which ligands to explore further, we compared the MM-GBSA_RBD-hACE2_ and MM-GBSA_Drug-(RBD+hACE2)_ energies as these values establish if the ligands inhibit or strengthen the interaction between the RBD and hACE2. We decided not to include the MM-GBSA_(RBD+Drug)-hACE2_ energies because these values, for most ligands, suggest a stable trimeric complex, and we want to focus on how these ligands act as inhibitors. Figure 5 compares these two MM-GBSA binding free energies and the percent dissociation of each ligand (except all poses of physcion because of its high percent dissociation). Also, the MM-GBSA binding free energies for both lisinoprils (-NH- and -NH_2_^+^-) are very similar, so for further analysis we focused on the -NH- lisinopril structure with an overall negative charge. All further reference to lisinopril will be to this structure. For Figure 5, the circular symbols represent the MM-GBSA_Drug-(RBD+hACE2)_ energies and the triangular symbols represent the percent dissociation, both with respect to the MM-GBSA_RBD-hACE2_ energies on the x-axis.

As shown in Figure 5, more favorable MM-GBSA_Drug-(RBD+hACE2)_ binding energies roughly correlate with low percent dissociation. However, diquafosol poses 1 and 3, and emodin pose 2 are outliers to this trend, i.e., in Figure 5 these complexes display percent dissociations less than 15% (purple triangles) and MM-GBSA_Drug-(RBD+hACE2)_ values more positive than −15 kcal/mol (purple circles). This would suggest that diquafosol and emodin may possess inhibitory characteristics, in that they potentially disrupt some amino acid interactions between the RBD and hACE2, but they are not the best RBD–hACE2 disruptor drug candidates as they do not bind well to the interface. Fosinoprilat in poses 2 and 3, fosinopril, and lisinopril have low percent dissociations (shown as orange triangles in Figure 5) and both MM-GBSA_Drug-(RBD+hACE2)_ (Figure 4A) and MM-GBSA_(RBD+Drug)-hACE2_ (Figure 4C) energies suggest relatively tight binding. These molecules are better disrupter candidates as they remain longer in the interface and the ligand binds relatively well to the dimer interface (negative MM-GBSA_Drug-(RBD+hACE2)_ values). Notably, all ligands showed that the ligand-bound RBD interacts favorably with hACE2 (negative MM-GBSA_(RBD+Drug)-hACE2_ values).

From our deconstruction of the different binding partners in the RBD-drug-hACE2 trimeric complex using MM-GBSA, our results suggest that it would be beneficial to further investigate the atomistic interactions of ligands that appear in Figure 5 as having low percent dissociation, with MM-GBSA_RBD-hACE2_ energetics similar to apo and MM-GBSA_Drug-(RBD+hACE2)_ more favorable than −20 kcal/mol. This includes fosinopril, fosinoprilat poses 2 and 3, and lisinopril. Specifically, to understand which individual interactions are inhibited or disrupted between the RBD and hACE2 and which interactions involving the ligand potentially cause the increase in strength in the interaction between the RBD and hACE2. The trajectories selected for further analysis are indicated in Figure 5 in yellow and are depicted in slightly larger symbols.

### Analysis of drugs–amino acid interactions

**FIGURE 6:**
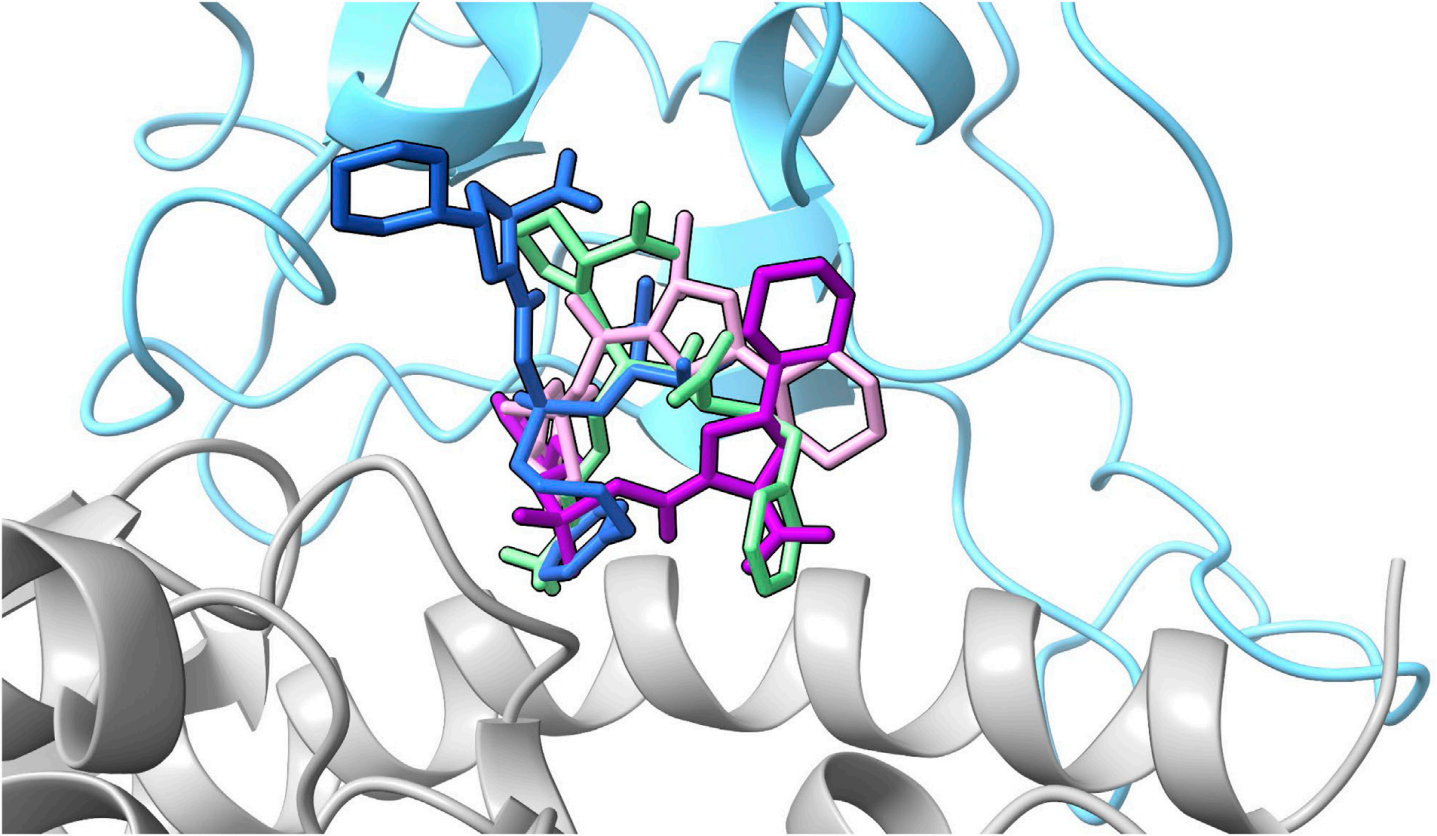
All ligand poses. The starting pose of each ligand selected for further analysis based on the evaluation of the MM-GBSA values are depicted in the interface. The SARS-CoV-2 RBD is depicted in blue and hACE2 in gray. Fosinopril in pose 1 is shown in dark blue, fosinoprilat in pose 2 is shown in purple, fosinoprilat in pose 3 is shown in pink, and lisinopril in pose 1 in light green.

Based on the energetic analyses conducted above, and summarized in Figure 5, we performed hydrogen bonding and pairwise decomposition analyses on fosinopril, fosinoprilat (poses 2 and 3), and lisinopril to better understand the amino acid interactions that occur upon ligand binding. These ACE inhibitors were selected because they consistently stayed bound within the interface and decreased the energy of interaction between the RBD and hACE2, based on the MM-GBSA energies detailed in the previous section. Figure 6 shows the starting pose of each ligand depicted in the RBD–hACE2 interface.

For all ligand-bound complexes selected for further analysis there is a significant decrease in the number of hydrogen bonds between the RBD and hACE2 upon ligand binding, signifying that the drugs disrupt interfacial interactions that were present in the apo ensemble (Table 5). For instance, in the apo complex there are 208 hydrogen bonds whereas in all ligand-bound complexes that number drops by almost half. Lisinopril has the largest number of hydrogen bonds between the RBD and the drug (52), and between hACE2 and the drug (112). It is also the only drug to have hydrogen bonds with itself. The formation of these hydrogen bonds stabilizes the ligand-bound complex. This is further supported by the MM-GBSA_Drug-(RBD+hACE2)_ [−31.80 (0.16) kcal/mol] and MM-GBSA_(RBD+Drug)-hACE2_ [−56.41 (0.20) kcal/mol] energies, which indicate a highly favorable interaction between the drug and the interface, and between the ligand-bound RBD and hACE2. Additional information pertaining to the hydrogen bonds involving lisinopril and the pairwise decomposition energies of the RBD–hACE2 interactions in the lisinopril-bound complex are in the Supplementary Material (Supplementary Tables S11, S12; Supplementary Figure S6). Fosinopril and fosinoprilat form hydrogen bonding interactions with the RBD and hACE2, but the total possible hydrogen bonds in these ligand-bound complexes are not greater than that for the apo, making them more ideal drug candidates. Additional information concerning the hydrogen bonds involving fosinopril or fosinoprilat and the pairwise decomposition energies of the RBD–hACE2 interactions in the ligand-bound complexes are in the Supplementary Material (Supplementary Tables S13–18; Supplementary Figures S7, S8). To further investigate the drugs’ effects, we studied these individual residue interactions.

**TABLE 5:** Total number of hydrogen bonds. The maximum number of hydrogen bonds between RBD and hACE2, RBD and the drug, hACE2 and the drug, and the drug with itself is listed below. All refers to the total number of possible hydrogen bonds between the entire complex, and the dash signifies that there are no hydrogen bonds present between the two structures.

Hydrogen bonds	Apo	Fosinopril	Fosinoprilat pose 2	Fosinoprilat pose 3	Lisinopril
All	208	184	179	191	274
RBD-hACE2	208	123	113	118	119
RBD-drug	-	31	40	48	52
hACE2-drug	-	30	26	25	112
drug-drug	-	-	-	-	13

Supplementary Table S19 contains hydrogen bonds between the RBD and hACE2 with a percent occurrence above 5% for the apo and the ligand-bound complexes with their corresponding pairwise decomposition energies. Notably, when the ligands are present in the interface, some hydrogen bonding interactions are no longer significant. For instance, the interactions RBD Tyr505 – hACE2 Ala386 and RBD Ser494 – hACE2 His34 are no longer significant upon ligand complexation. There is a notable increase in the pairwise decomposition energies for the interaction RBD Ser494–hACE2 His34 in each ligand bound complex.

**FIGURE 7:**
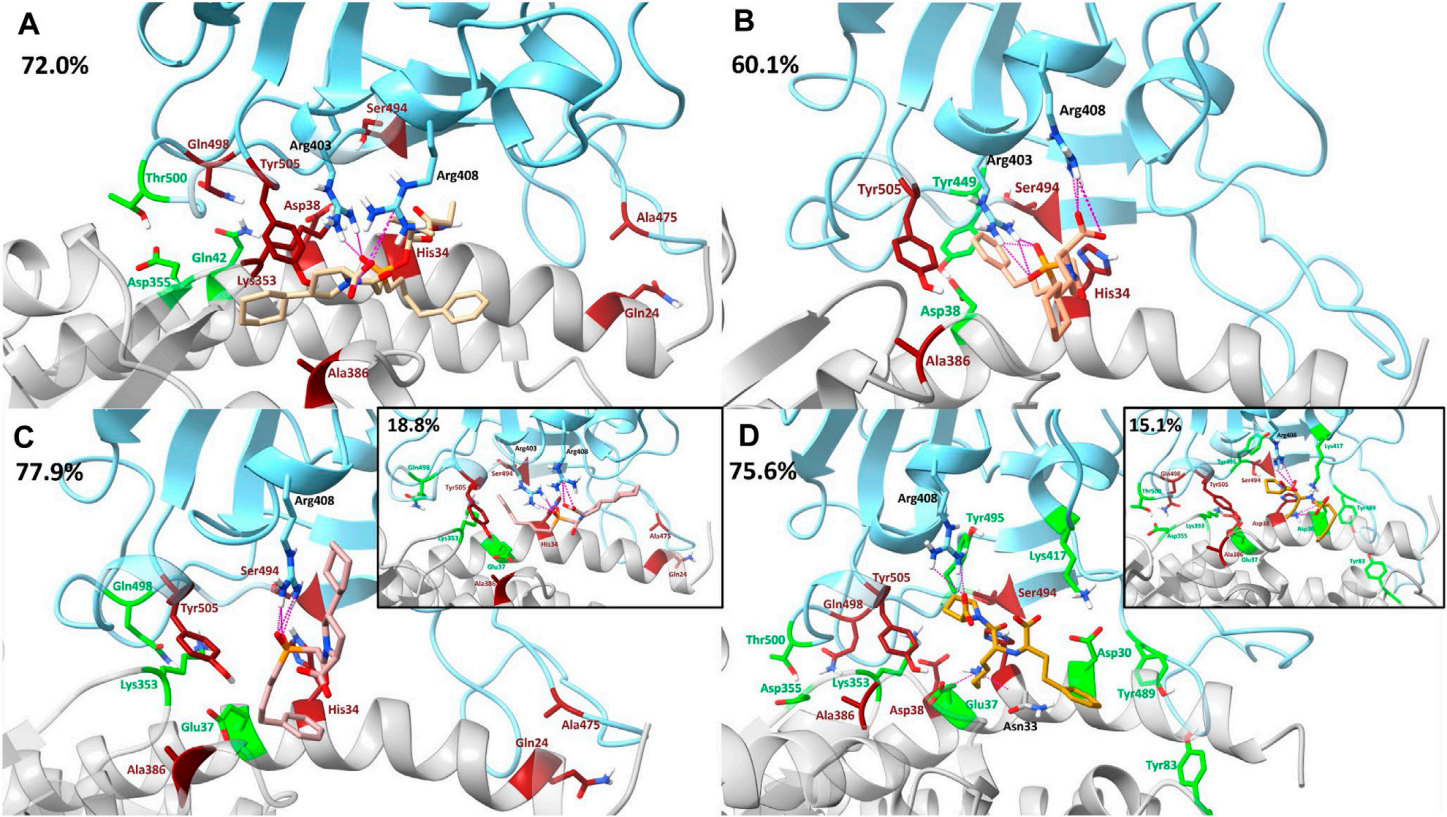
Visualization of drugs’ effects on residue interactions using dominant structures determined from clustering. The SARS-CoV-2 RBD is shown in blue and the hACE2 receptor is shown in gray. Clustering was used to determine the dominant structure of the ligand-bound complex throughout the 500 ns simulation. The percentage of the simulation that is represented by this dominant structure is in the top left corner of each panel. Hydrogen bonds between the drug and the RBD–hACE2 complex are depicted in pink. In all clusters, such hydrogen bonding persisted for at least 12% or more of each trajectory. Residue interactions between the RBD and hACE2 that were enhanced are shown in green and interactions that were disrupted are shown in red. **(A)** Fosinopril, depicted in beige, is bound in the interface. **(B)** Fosinoprilat pose 2, depicted in orange, is bound in the interface. **(C)** Fosinoprilat pose 3, depicted in light pink, is bound in the interface. Hydrogen bonds that were not present in the first dominant structure were visualized in the second dominant structure. **(D)** Lisinopril, depicted in brown, is bound in the interface. Hydrogen bonds that were not present in the first dominant structure were visualized in the second dominant structure.

Supplementary Tables S20–S23 contain the pairwise decomposition energies for interactions between the ligand and the RBD - hACE2 complex for energies more favorable than −1 kcal/mol. Notably, in all ligand bound complexes, except lisinopril, the ligand participates in a significant van der Waals interaction with the residue RBD Tyr505. Additionally, in all ligand bound complexes, a significant van der Waals interaction occurs between the ligand and hACE2 Ala387. From a visual inspection of the MD trajectories of all ligand-bound complexes we observe hACE2 Ala387 interacting with each ligand and moving the backbone oxygen of hACE2 Ala386 further away from RBD Tyr505. This limits the opportunity for a hydrogen bond to occur between that oxygen and the hydroxyl group of the Tyr505 side chain. However, it was primarily observed that the ligand (except lisinopril) interactions with RBD Tyr505 are responsible for interfering with the residue interaction. Also, all ligands participate in significant van der Waals or electrostatic interactions with hACE2 His34, which we observe to interfere with the hydrogen bond between RBD Ser494 - hACE2 His34 and RBD Tyr453–hACE2 His34. However, the disruption of the interaction RBD Ser494–hACE2 His34 was more prominent due to the orientation of hACE2 His34 throughout the simulation. This additional data further supports that the presence of each ligand in the interface disrupts RBD–hACE2 interactions. Other residue interactions were disrupted (red) or enhanced (green) depending on the drug-bound complex and these interactions are visualized in Figure 7 and discussed in detail in the Supplementary Material. Based on the dominant interactions shown in Figure 7, lisinopril enhances the most RBD - hACE2 interactions, while fosinopril disrupts the most.

Table 6 lists the sum of all pairwise decomposition energies between the RBD and hACE2 for the apo and ligand-bound complexes, which is another way to evaluate the binding interaction. The sum of pairwise interaction energies of the RBD - hACE2 interactions for the fosinopril and fosinoprilat pose 2 bound complexes are more positive compared to the apo sum, which suggest that these drugs inhibit RBD - hACE2 interactions. On the other hand, the sum for the fosinoprilat pose 3 and lisinopril bound complexes are more negative compared to the apo sum, which support that they enhance RBD - hACE2 interactions more than inhibit them. This trend is further supported by the previously discussed interactions mentioned in the manuscript (Figure 7) and the Supplementary Material. Overall, hydrogen bonding and pairwise decomposition analyses support the idea that these ligands form favorable interactions in the interface with the RBD and hACE2. Fosinopril and fosinoprilat pose 2 display inhibitory properties while fosinoprilat pose 3 and lisinopril strengthen the RBD–hACE2 complex.

**TABLE 6:** Sum of pairwise decomposition energies. This table lists the sum of the average pairwise interaction energies between the RBD and hACE2 in the apo complex or the specified ligand-bound complexes. The standard deviation and standard error for all totals is also listed.

	Apo	Fosinopril	Fosinoprilat pose 2	Fosinoprilat pose 3	Lisinopril
Pairwise Total ± Std. Dev. (Std. Error)	−118.92 ± 0.050 (0.00015)	−113.42 ± 0.048 (0.00014)	−118.50 ± 0.050 (0.00015)	−120.85 ± 0.053 (0.00015)	−122.7 ± 0.055 (0.00016)

As noted in the apo section above, approximately 28% of the S1 subunit of the SARS-CoV-2 spike protein contains experimentally determined intrinsically disordered regions (IDRs). (Quaglia et al., 2022). Of the three IDRs previously identified, RBD residues 455—490 occur at the interface with hACE2. To assess the conformational behavior of these residues, as well as the impact that ligand binding has on protein dynamics, we conducted native coÅntact analysis on each ligand-bound complex. Supplementary Figures S9–12 represent the fraction of native contacts (Q(X) for each frame of the ligand-bound complex simulations. Q values remain above 90% for the bulk of each trajectory, suggesting that the ligands are not causing significant changes in RBD—hACE2 conformation and that the disordered region near residues 455–490 is conformational stable. There are a few short regions where Q decreases to ∼83%, however visualization of the trajectories did not reveal any significant conformational changes during these periods., We were also interested in studying how the ligands affected the SARS-CoV-2 RBD dynamics, so we conducted secondary structure analysis on the residues in the RBM and a range of residues that includes RBD amino acids found to have significant hydrogen bonding interactions with each ligand. Supplementary Tables S24–S28; Supplementary Figures S13–17 contain the secondary structure plots for the apo and ligand-bound complexes. There were few notable differences in the secondary structure of the apo and ligand-bound complexes, which are further explained in detail in the Supplementary Material.

We also explored water-mediated interactions between fosinopril, fosinoprilat (pose 2 and 3) and lisinopril with residues in the RBD-hACE2 interface (Supplementary Tables S29–S32). All drugs participate in such interactions with residues RBD Glu406, hACE2 Ala387, RBD Arg403, RBD Arg408, and a hACE2 glycan located near the interface. Notably, the water-mediated interaction between Arg408 and each drug led to a consistent interaction throughout the simulation, with hydrogen bond occurrences greater than 20%. Additionally, the water-mediated interaction between Arg403 and fosinopril or fosinoprilat pose 2 formed a persisent hydrogen bond with occurrences of ∼55% and ∼20%, respectively (Supplementary Tables S14, 15). Lastly, each drug also participates in a limited number of bridged interactions between multiple RBD or hACE2 residues. However, these binary interactions have relatively low percent occurrences in most cases.

## Conclusion

The interaction between the SARS-CoV-2 RBD and the hACE2 receptor leads to viral infection. Few studies have screened or simulated potential RBD inhibitors in the complex junction formed by the RBD and hACE2. While most studies docked solely to the RBD, we found that it was important to dock all ligands to a dimeric complex to better understand interfacial binding of potential inhibitors. Evaluating inhibitors based only on the estimated binding affinity between the RBD and the inhibitor ignores the effect of hACE2. In the present study, ACE inhibitors and previously identified potential SARS-CoV-2 spike protein inhibitors were docked in the RBD - hACE2 interface. As a result of the docking analysis, molecular dynamics simulations of fosinopril, fosinoprilat, lisinopril, emodin, diquafosol, and physcion bound to the interface were performed. Based on the MM-GBSA analysis, it was found that all selected ligands bind favorably to the interface and most reduce the stability of the RBD–hACE2 interaction. However, in addition to these inhibitory characteristics, some ligands such as fosinopril, fosinoprilat (in poses 2 and 3) and lisinopril strengthen the interaction of the trimeric complex. Hydrogen bonding and pairwise decomposition analyses were performed on these three ligands. We found that while the maximum number of hydrogen bonds between the RBD and hACE2 were reduced, suggesting that all ligands are capable of inhibiting interactions, the RBD or hACE2 interactions that formed with lisinopril or fosinoprilat in pose 3 were able to stabilize the complex. When either lisinopril or fosinoprilat in pose 3 were bound in the interface, the ligands were able to strengthen the trimeric complex, making it more stable than the apo complex. Additionally, our results suggest that fosinopril most effectively prevented the formation of RBD–hACE2 residue interactions and was the best RBD inhibitor.

## Data Availability

The datasets presented in this study can be found at https://github.com/Parish-Lab/disrupting_SARSCoV2_RBD_hACE2.
