# Supplementary material for: Exploring the disruption of SARS-CoV-2 RBD binding to hACE2
Source: Front Chem. 2023 Oct 24;11:1276760. doi: 10.3389/fchem.2023.1276760 (PMC10635427; doi:10.3389/fchem.2023.1276760)
Supplement: Supplementary file 1 [file DataSheet1.PDF]

# Supporting Information: Exploring the disruption of SARS-CoV-2 RBD binding to hACE2

**Camryn Carter,[A] Justin Airas,[A] Haley Gladden,[A] Bill R. Miller III,[B] and Carol A. Parish[A,*]**

**A. Department of Chemistry, Gottwald Center for the Sciences, University of Richmond, Richmond, VA, 23173, USA**
**B. Department of Chemistry, Truman State University, Kirksville, MO 63501, USA**

*** Corresponding author**

## Apo

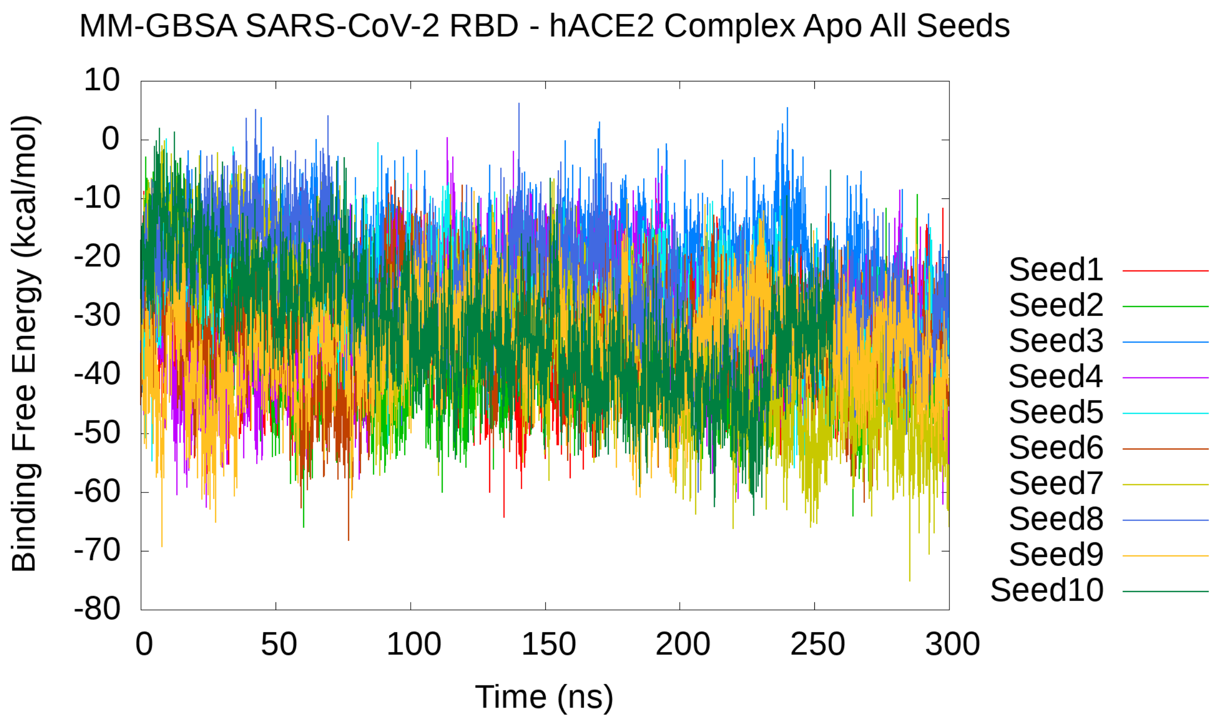


**Figure S1.** Apo MM-GBSA binding energies for RBD - hACE2 complex. The MM-GBSA binding energies are reported for the entire 300ns simulation and for each seed. The corresponding color for each seed is indicated in the key.

**Table S1.** Apo MM-GBSA binding energies for each seed. The average MM-GBSA binding energies for all ten seeds across 300ns simulations.

| Seeds | Apo MM-GBSA (kcal/mol) | Std Dev. (kcal/mol) | Std. Error (kcal/mol) |
|---|---|---|---|

| | | | |
|---|---|---|---|
| 1 | -33.69 | 8.38 | 0.15 |
| 2 | -36.83 | 9.30 | 0.17 |
| 3 | -21.11 | 7.81 | 0.14 |
| 4 | -31.84 | 9.54 | 0.17 |
| 5 | -27.93 | 8.07 | 0.15 |
| 6 | -34.36 | 8.73 | 0.16 |
| 7 | -33.56 | 12.83 | 0.23 |
| 8 | -24.74 | 8.97 | 0.16 |
| 9 | -35.60 | 8.66 | 0.16 |
| 10 | -32.60 | 11.03 | 0.22 |

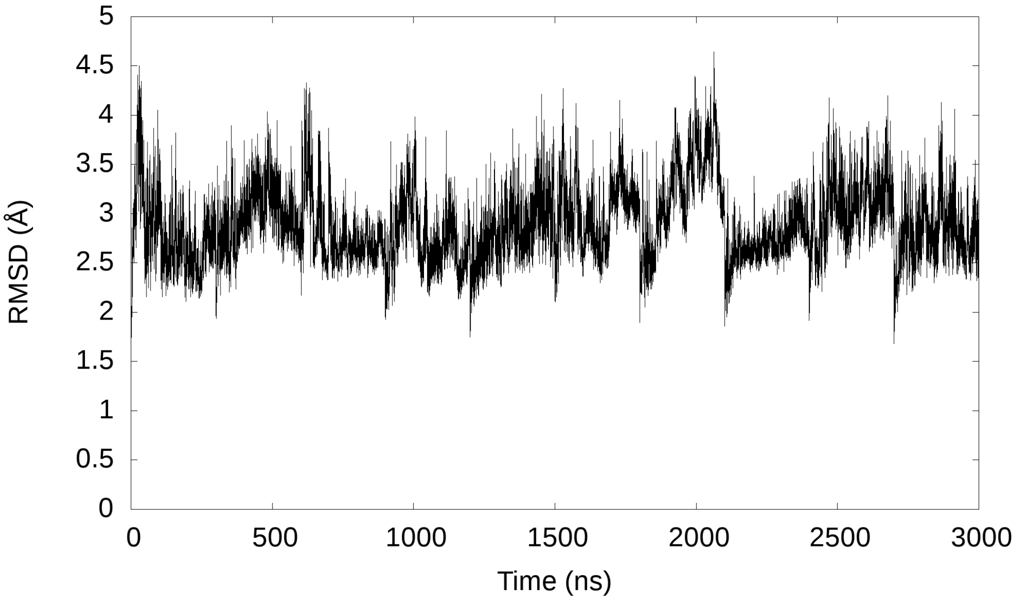


**Figure S2.** RMSD of SARS-CoV-2 RBD - hACE2 complex. The RMSD is calculated for all residue backbones and side chains for the 3 μs ensemble.

**Table S2.** SARS-CoV-2 RBD - hACE2 Apo pairwise decomposition energies. Listed in the table are RBD residues and hACE2 residues, and the corresponding average pairwise decomposition energy. Interactions were selected if pairwise decomposition was less than -2 kcal/mol.

| RBD Residue | hACE2 Residue | van der Waals | Electrostatic | Pairwise Decomposition |
|---|---|---|---|---|

| | | | | Avg. ± Std. Dev. (kcal/mol) |
|---|---|---|---|---|
| Lys417 | Asp30 | 0.17 ± 0.68 | -43.56 ± 7.74 | -6.52 ± 2.94 |
| Tyr505 | Lys353 | -2.21 ± 0.36 | -1.63 ± 0.79 | -5.00 ± 0.54 |
| Asn501 | Lys353 | -1.61 ± 0.29 | -3.63 ± 2.02 | -4.73 ± 1.46 |
| Thr500 | Asp355 | -0.66 ± 0.59 | -3.11 ± 3.25 | -4.70 ± 2.44 |
| Gln493 | Glu35 | -0.47 ± 0.55 | -4.74 ± 2.82 | -4.49 ± 1.80 |
| Gln493 | Lys31 | -0.14 ± 0.54 | -6.90 ± 3.91 | -4.13 ± 1.95 |
| Gln498 | Lys353 | -0.15 ± 0.46 | -4.90 ± 4.84 | -3.86 ± 3.30 |
| Tyr449 | Asp38 | 0.09 ± 0.72 | -6.08 ± 4.07 | -3.59 ± 2.01 |
| Gly496 | Lys353 | 0.10 ± 0.56 | -5.17 ± 2.41 | -3.25 ± 1.77 |
| Asn487 | Tyr83 | -0.05 ± 0.55 | -3.37 ± 1.39 | -2.89 ± 0.96 |
| Asn487 | Gln24 | -0.96 ± 0.40 | -1.25 ± 1.27 | -2.87 ± 0.93 |
| Tyr505 | Glu37 | -0.19 ± 0.61 | -4.36 ± 4.32 | -2.86 ± 2.38 |
| Phe486 | Met82 | -1.22 ± 0.42 | -0.01 ± 0.27 | -2.47 ± 0.78 |
| Asn501 | Tyr41 | -0.73 ± 0.36 | -0.60 ± 0.64 | -2.33 ± 1.25 |
| Gln493 | His34 | -1.02 ± 0.37 | -0.83 ± 1.42 | -2.12 ± 1.23 |
| Tyr453 | His34 | -0.54 ± 0.40 | -1.60 ± 1.32 | -2.07 ± 1.05 |

**Table S3.** The average hydrogen bonding occurrences for the 3000 ns ensemble. Below are hydrogen bonding percentages that were greater than 5%.

| Acceptor | Donor | Hydrogen Bonding Percent Occurrence (avg.) |
|---|---|---|
| RBD - Asn487 @ OD1 | hACE2 - Tyr83 @ HH-OH | 69.56 |
| hACE2 - Lys353 @ O | RBD - Gly502 @ H-N | 66.80 |
| hACE2 - Asp355 @ OD1, OD2 | RBD - Thr500 @ HG1-OG1 | 42.19 |

| | | |
|---|---|---|
| hACE2 - Asp38 @ OD1, OD2 | RBD - Tyr449 @ HH-OH | 59.72 |
| hACE2 - Tyr41 @ OH | RBD - Thr500 @ HG1-OG1 | 29.37 |
| hACE2 - Glu35 @ OE1, OE2 | RBD - Gln493 @ HE21-NE2, HE22-NE2 | 52.44 |
| hACE2 - His34 @ ND1 | RBD - Tyr453 @ HH-OH | 24.98 |
| hACE2 - Glu37 @ OE1, OE2 | RBD - Tyr505 @ HH-OH | 44.40 |
| hACE2 - Gln24 @ OE1 | RBD - Asn487 @ HD21-ND2, HD22-ND2 | 19.62 |
| RBD - Ala475 @ O | hACE2 - Ser19 @HG-OG | 17.37 |
| RBD - Gln493 @ OE1 | hACE2 - Lys31 @ HZ1, HZ2, HZ3-NZ | 36.74 |
| RBD - Gln498 @ OE1 | hACE2 - Lys353 @ HZ1, HZ2, HZ3-NZ | 34.90 |
| RBD - Ala475 @ O | hACE2 - Gln24 @ HE21-NE2, HE22-NE2 | 11.76 |
| hACE2 - Asp38 @ OD1, OD2 | RBD - Gln498 @ HE21-NE2, HE22-NE2 | 19.52 |
| hACE2 - Asp30 @ OD1, OD2 | RBD - Lys417 @ HZ1, HZ2, HZ3-NZ | 57.81 |
| hACE2 - Ala386 @ O | RBD - Tyr505 @ HH-OH | 10.08 |
| RBD - Ser494 @ O | hACE2 - His34 @ HE-NE2 | 9.93 |
| RBD - Gly496 @ O | hACE2 - Lys353 @ HZ1, HZ2, HZ3-NZ | 26.47 |
| RBD - Gly446 @ O | hACE2 - Gln42 @ HE21-NE2, HE22-NE2 | 9.02 |
| RBD - Tyr495 @ O | hACE2 - Lys353 @ HZ1, HZ2, HZ3-NZ | 7.75 |

**Table S4.** SARS-CoV-2 RBD - hACE2 hydrogen bonds and pairwise decomposition energies.

Hydrogen bonding occurrences for residue interactions between the SARS-CoV-2 RBD and the hACE2 receptor. For each hydrogen bonding interaction represented in the table, the corresponding pairwise decomposition energy of said interaction is listed. Pairwise decomposition is calculated from 30,000 frames from the full 3000 ns ensemble. The table includes hydrogen bonding occurrences greater than 5%.

| RBD Residue | hACE2 Residue | Hydrogen Bonding Percent Occurrence (avg.) | Pairwise Decomposition (avg. ± std. dev.) (kcal/mol) |
|---|---|---|---|
| Asn487 | Tyr83 | 69.56 | -2.89 ± 0.96 |
| | Gln24 | 19.62 | -2.87 ± 0.93 |
| Gly502 | Lys353 | 66.80 | -1.63 ± 0.32 |
| Tyr449 | Asp38 | 59.72 | -3.59 ± 2.01 |
| Lys417 | Asp30 | 57.81 | -6.52 ± 2.94 |
| Gln493 | Glu35 | 52.44 | -4.50 ± 1.80 |
| | Lys31 | 36.74 | -4.13 ± 1.95 |
| Tyr505 | Glu37 | 44.40 | -2.86 ± 2.38 |
| | Ala386 | 10.08 | -0.50 ± 0.82 |
| Thr500 | Asp355 | 42.19 | -4.70 ± 2.44 |
| | Tyr41 | 29.68 | -1.26 ± 1.46 |
| Gln498 | Lys353 | 34.90 | -3.86 ± 3.30 |
| | Asp38 | 19.52 | -1.68 ± 2.27 |
| Gly496 | Lys353 | 26.47 | -3.25 ± 1.77 |
| Tyr453 | His34 | 24.98 | -2.07 ± 1.05 |
| Ala475 | Ser19 | 17.37 | -0.73 ± 1.29 |

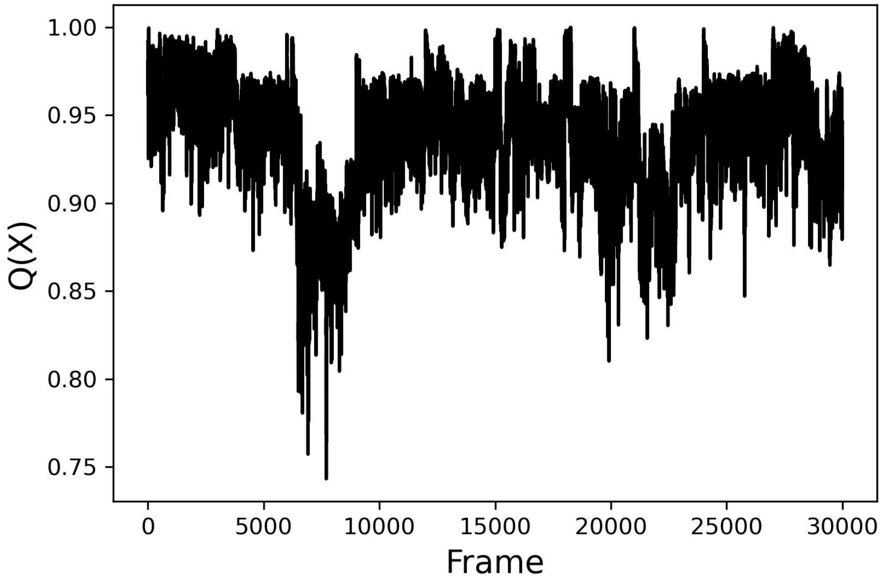


**Figure S3.** Native contact analysis in the interface of apo 3 μs ensemble. The fraction of native contacts is plotted for each frame.

Notably, there is a decrease in the fraction of native contacts between frames 6,493 and 8,550, which occurs in seed 3. After visualizing the 3 μs ensemble, we did not identify a noticeable difference in the SARS-CoV-2 RBD and hACE2 interface during these frames. However, we found a notable decrease in pairwise decomposition energies of residue interactions during seed 3 as compared to the average 3 μs ensemble, which supports the reduction in the fraction of native contacts. Interactions such as RBD Gln498 - hACE2 Lys353 (-0.18 (0.04) kcal/mol), RBD Gly496 - hACE2 Lys353 (-0.81 (0.07) kcal/mol), RBD Tyr505 - hACE2 Glu37 (-0.37 (0.08) kcal/mol), and RBD Asn501 - hACE2 Tyr41 (-0.37 (0.04) kcal/mol) have low pairwise decomposition energies compared to the average over 10 seeds (Table S2). Notably, seed 3 has the lowest average MM-GBSA binding energy (-21.11 (0.14) kcal/mol), suggesting that this seed sampled a region of the potential energy surface where these interactions were unfavorable (Table S1).

## Site Map

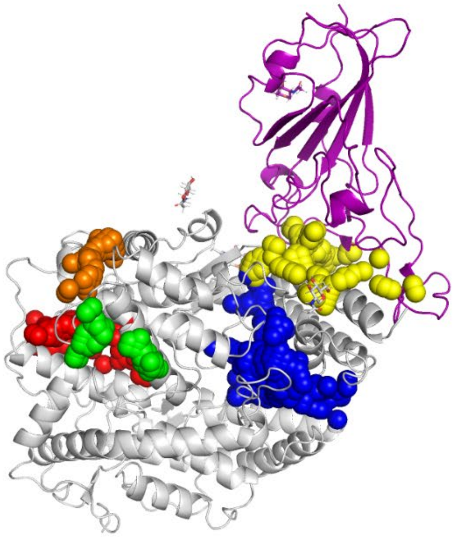

**Figure S4.** SiteMap results for 6LZG. Blue indicates Site #1 (SScore 1.005, DScore 1.029), yellow indicates Site #2 (SScore 1.002, DScore 1.017), red indicates Site #3 (SScore 1.053, DScore 1.061), green indicates Site #4 (SScore 0.743, DScore 0.742), and orange indicates Site #5 (SScore 0.696, DScore 0.672). Site #2 occurs in the hACE2 – SARS-CoV-2 RBD complex junction and is the binding site of interest.

**Table S5.** SiteMap properties for 6LZG binding sites.

| Site # | Size | Volume | Exposure | Enclosure | Contact | Phobic | Philic | Balance | Don/Acc |
|---|---|---|---|---|---|---|---|---|---|
| 1 | 251 | 885.28 | 0.645 | 0.696 | 0.352 | 0.352 | 1.010 | 0.348 | 1.088 |
| 2 | 164 | 568.35 | 0.697 | 0.702 | 0.854 | 0.236 | 1.057 | 0.223 | 0.711 |
| 3 | 133 | 463.74 | 0.567 | 0.777 | 1.008 | 0.712 | 1.060 | 0.672 | 0.831 |
| 4 | 56 | 120.05 | 0.735 | 0.546 | 0.685 | 0.225 | 0.887 | 0.254 | 0.948 |
| 5 | 50 | 117.65 | 0.725 | 0.549 | 0.737 | 0.156 | 0.989 | 0.158 | 1.392 |

## Epik and Glide Docking

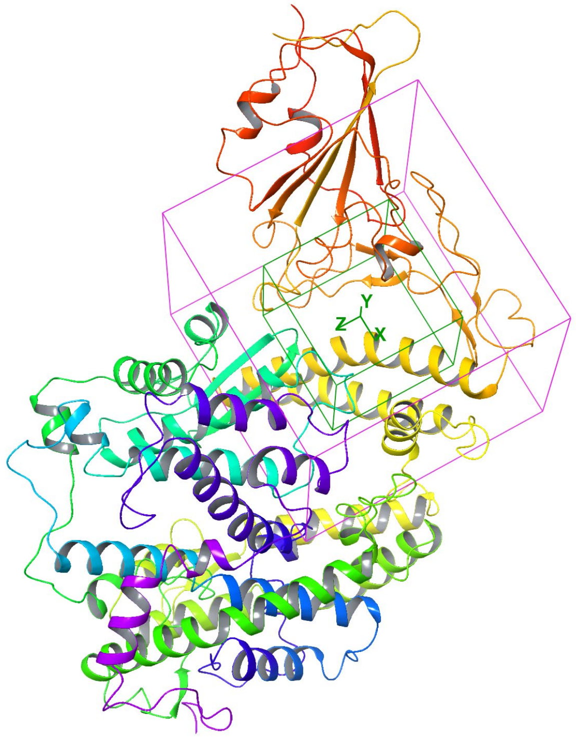


**Figure S5.** SARS-CoV-2 RBD and hACE2 complex receptor grid for ligand docking.

**Table S6.** GlideScores of all ligands. A ranking for each ligand is based on the XP GlideScore of the first pose.

| Drug Name | 6LZG | |
|---|---|---|
| | GlideScore (kcal/mol) | Rank |
| Benazepril | -4.154 | 8 |
| Captopril | -3.588 | 13 |
| Enalapril | -4.097 | 9 |
| Fosinopril | -6.050 | 2 |
| Fosinoprilat | -3.287 | 16 |
| Lisinopril | -4.959 | 3 |
| Lisinopril (-1) | -3.353 | 15 |
| Perindopril | -3.569 | 14 |
| Quinapril | -3.984 | 11 |
| Ramipril | -4.070 | 10 |
| Trandolapril | -3.643 | 12 |
| Aloe Emodin LS-H15204 | -4.384 | 7 |
| Camostat LS-H6976 | -2.731 | 17 |
| Emodin LS-H11074 | -4.706 | 5 |
| Emodin LS-H17409 | -4.402 | 6 |
| Physcion LS-H9395 | -4.737 | 4 |
| Diquafosol | -9.138 | 1 |

**Table S7**. Top three poses of each ligand. The top three poses of each ligand and their corresponding GlideScores are listed. The rank was decided based on the GlideScore of the first pose of each ligand.

| Rank | Ligands | GlideScore (kcal/mol) |
|---|---|---|
| 1 | Diquafosol P1 | -9.138 |
| | Diquafosol P2 | -8.914 |
| | Diquafosol P3 | -8.406 |

| | | |
|---|---|---|
| 2 | Fosinopril P1 | -6.050 |
| | Fosinopril P2 | -5.683 |
| | Fosinopril P3 | -5.675 |
| 3 | Lisinopril P1 | -4.959 |
| 4 | Physcion P1 | -4.737 |
| | Physcion P2 | -4.657 |
| | Physcion P3 | -4.619 |
| | Lisinopril P2 | -4.874 |
| | Lisinopril P3 | -4.861 |
| 5 | Emodin_H11074 P1 | -4.706 |
| | Emodin_H11074 P2 | -4.542 |
| 6 | Emodin_H17409 P1 | -4.402 |
| 7 | Aloe Emodin P1 | -4.384 |
| | Aloe Emodin P2 | -4.316 |
| | Aloe Emodin P3 | -4.298 |
| | Emodin_H11074 P3 | -4.294 |
| | Emodin_H17409 P2 | -4.182 |
| 8 | Benazepril P1 | -4.154 |
| | Benazepril P2 | -4.130 |
| 9 | Enalapril P1 | -4.097 |
| 10 | Ramipril P1 | -4.070 |
| 11 | Quinapril P1 | -3.984 |
| | Ramipril P2 | -3.928 |
| | Quinapril P2 | -3.819 |
| | Enalapril P2 | -3.739 |
| | Quinapril P3 | -3.730 |

| | | |
|---|---|---|
| 12 | Trandolapril P1 | -3.643 |
| | Trandolapril P2 | -3.638 |
| | Enalapril P3 | -3.631 |
| | Emodin_H17409 P3 | -3.618 |
| 13 | Captopril P1 | -3.588 |
| 14 | Perindopril P1 | -3.569 |
| | Benazepril P3 | -3.547 |
| | Captopril P2 | -3.499 |
| 15 | Lisinopril NH P1 | -3.353 |
| 16 | Fosinoprilat P1 | -3.287 |
| | Trandolapril P3 | -3.186 |
| | Perindopril P2 | -3.135 |
| | Lisinopril NH P2 | -3.087 |
| | Fosinoprilat P2 | -3.083 |
| | Captopril P3 | -2.925 |
| | Fosinoprilat P3 | -2.918 |
| | Lisinopril NH P3 | -2.889 |
| | Ramipril P3 | -2.882 |
| 17 | Camostat P1 | -2.731 |
| | Benazepril NH P1 | -2.666 |
| | Fosinopril P3 | -2.608 |
| | Benazepril NH P2 | -2.549 |
| | Benazepril NH P3 | -2.478 |
| | Camostat P2 | -2.316 |
| | Camostat P3 | -2.307 |

**Table S8.** Ligand Interaction diagrams. Below includes the ligand interaction diagrams for the top 3 poses of each ligand selected for MD analysis.

| Ligands | Pose 1 | Pose 2 | Pose 3 |
|---|---|---|---|
| Diquafosol | 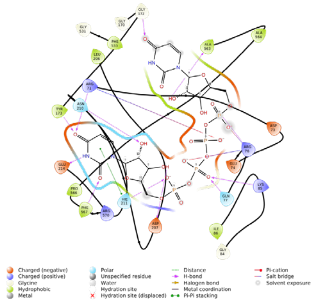 | 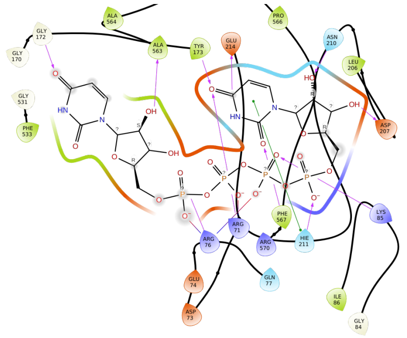 | 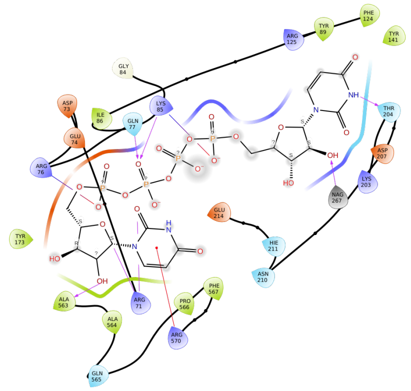 |
| Emodin_H11074 | 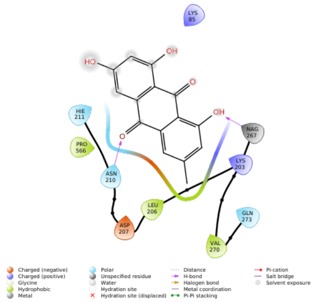 | 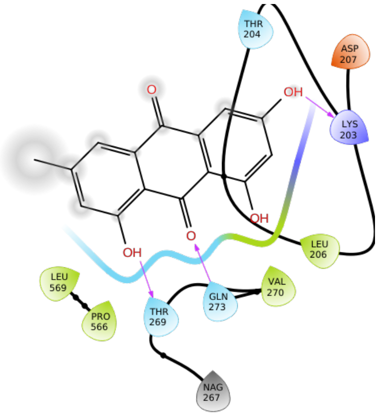 | 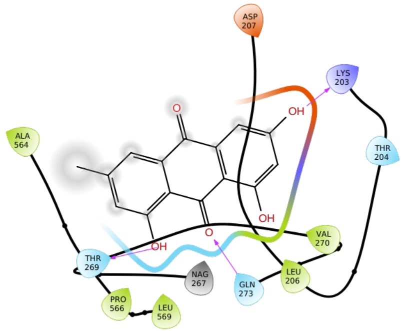 |
| Fosinopril | 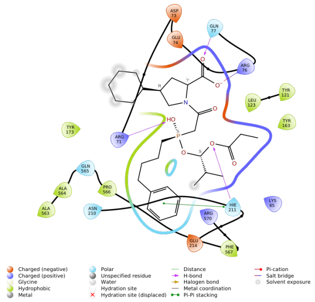 | 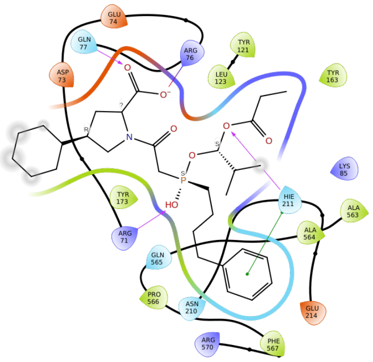 | 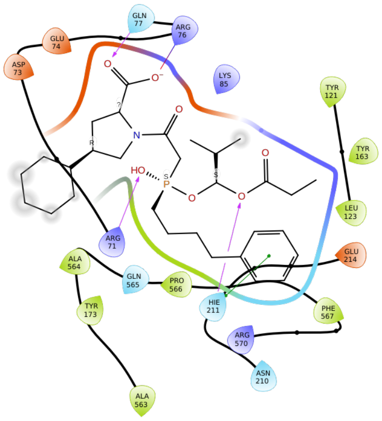 |

| | | | |
|---|---|---|---|
| Fosinoprilat | 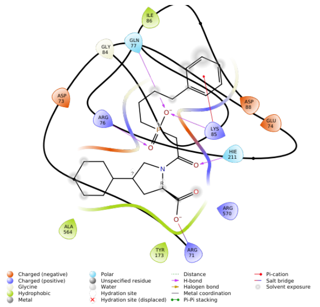 | 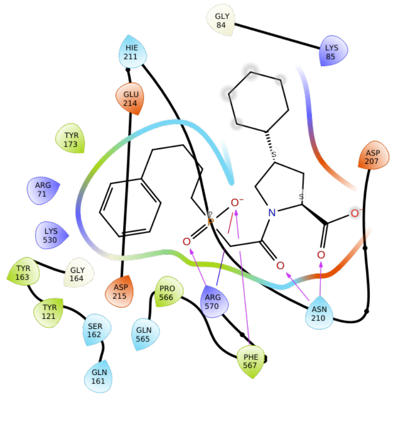 | 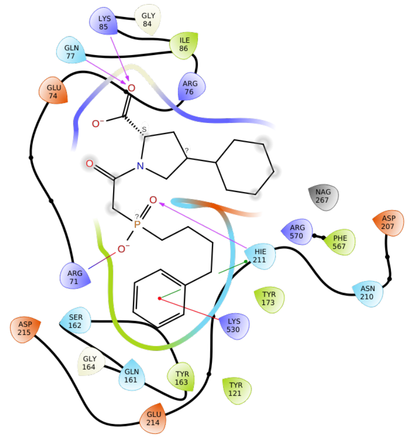 |
| Lisinopril (0) | 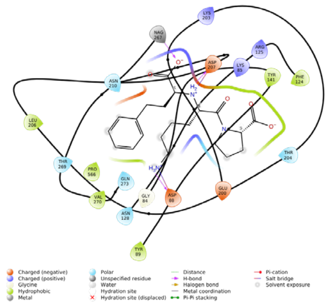 | 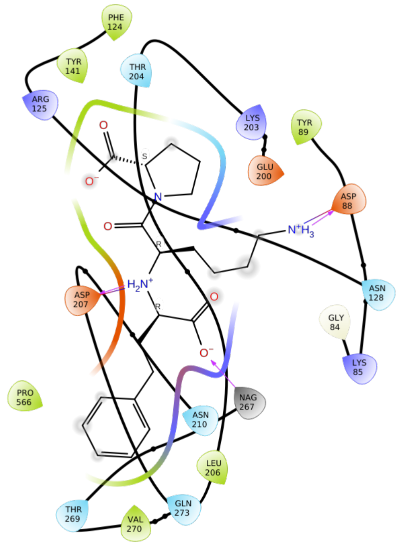 | 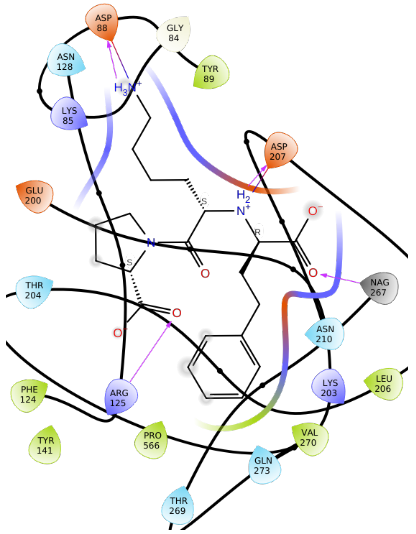 |
| Lisinopril (-1) | 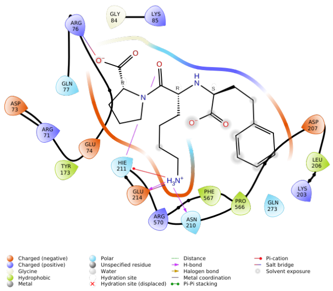 | 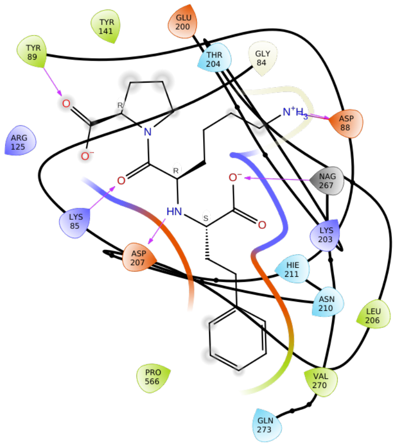 | 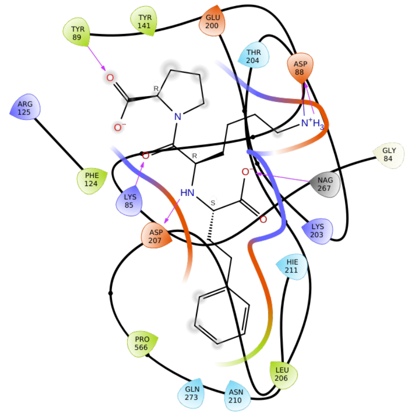 |

| | | | |
|---|---|---|---|
| Physcion | 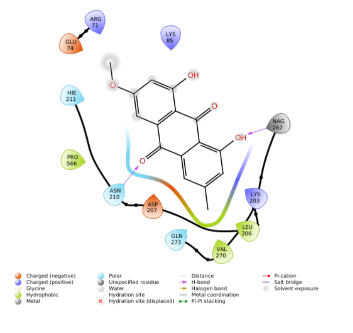 | 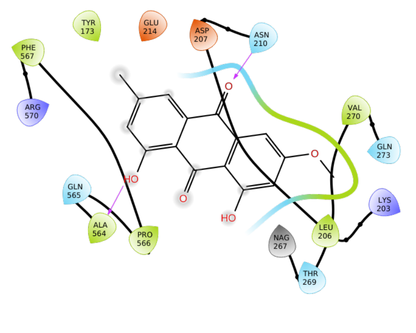 | 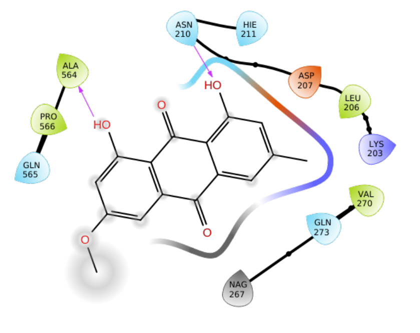 |

## ADME and PAINS

**Table S9.** ADME properties for seven selected ligands. ADME properties for diquafosol, emodin, fosinopril, fosinoprilat, lisinopril with -$NH_2^+$- backbone nitrogen, lisinopril with -NH- backbone nitrogen, and physcion. H-bond donor and acceptor refer to the average number of hydrogen atoms on the molecule that is estimated to be capable of donating or accepting a hydrogen bond, respectively. logP Octanol/Water and logS Aqueous Solubility refers to the solubility of the ligand. logBB Brain/Blood refers to how likely the drug is able to cross the blood-brain barrier. Lastly, $logIC_{50}$ HERG $K^+$ refers to the amount (in log scale) that would need to be taken to block the HERG $K^+$ channel. Data that exceeds the range of 95% of known drugs used in the ADME program are underlined, and data that was flagged due to the molecular weight of the ligand exceeding the trained set of compounds is bolded.

| Ligand | Molecular Weight (AMU) | H-Bond Donor | H-Bond Acceptor | logP Octanol/Water | logS Aqueous Solubility | logBB Brain/Blood | $logIC_{50}$ HERG $K^+$ |
|---|---|---|---|---|---|---|---|
| **Diquafosol** | 790.311 | 6.000 | 33.200 | -5.404 | **0.031** | 6.000 | **1.064** |
| **Emodin** | 270.241 | 1.000 | 4.250 | 1.252 | -3.051 | -1.535 | -4.330 |
| **Fosinopril** | 563.670 | 1.000 | 12.000 | 4.677 | -5.917 | -1.605 | -3.374 |
| **Fosinoprilat** | 435.499 | 1.000 | 9.000 | 3.540 | -4.264 | -1.880 | -0.732 |
| **Lisinopril ($NH_2^+$)** | 405.493 | 5.000 | 9.500 | -1.209 | -1.142 | -1.944 | -1.828 |
| **Lisinopril (NH)** | 405.493 | 5.000 | 9.500 | -1.199 | -1.080 | -1.494 | -1.889 |
| **Physcion** | 284.268 | 0.00 | 4.250 | 1.849 | -3.978 | -1.045 | -4.366 |

As mentioned in Table S9, diquafosol exceeds the indicated range for the properties: molecular weight, H-bond acceptor, and logP octanol/water. This analysis indicated that diquafosol is water soluble, which is further supported given that it is a common compound in eye drops. Additionally, the molecular structure of diquafosol is notably uncommon with four chained phosphate groups, so it is possible that the ADME program is not trained on molecules of similar structures. The ADME program noted that diquafosol exceeds the molecular weight of the drugs trained for the properties of aqueous solubility and $logIC_{50}$. Again, this could be due to the distinct structure of the molecule.

## Ligand-Bound MD Simulations

**Table S10.** RMSD for all drug-bound complexes selected for MD simulations.

| Ligands | Pose 1 | Pose 2 | Pose 3 |
|---|---|---|---|
| Diquafosol | 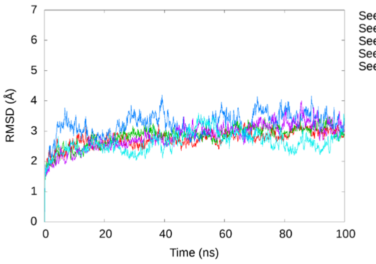 | - | 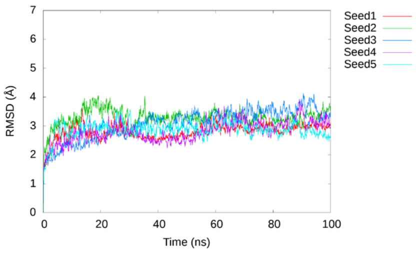 |
| Emodin_H1 1074 | 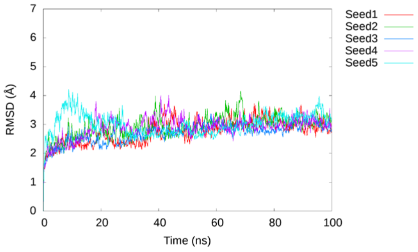 | 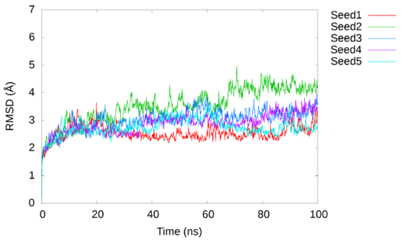 | - |
| Fosinopril | 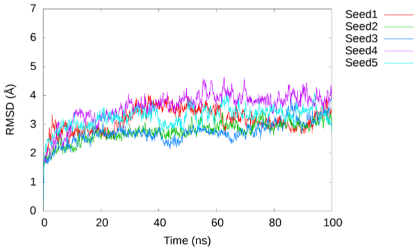 | - | - |

| | | | |
|---|---|---|---|
| Fosinoprilat | 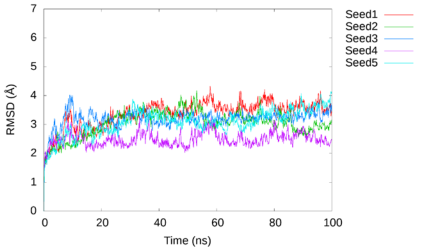 | 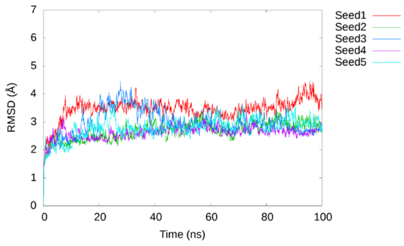 | 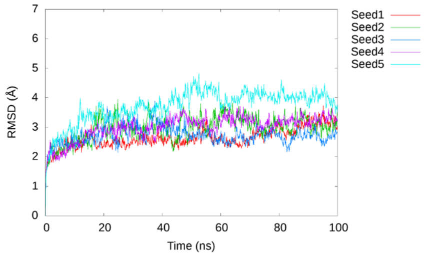 |
| Lisinopril ($NH_2^+$) | 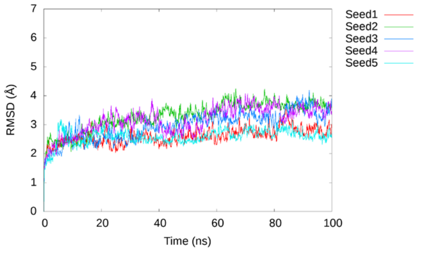 | - | - |
| Lisinopril (NH) | 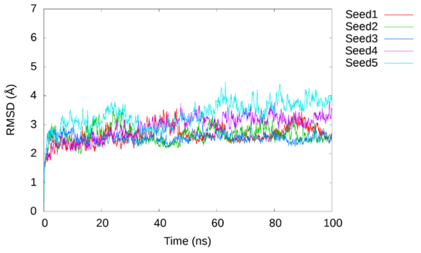 | - | - |
| Physcion | 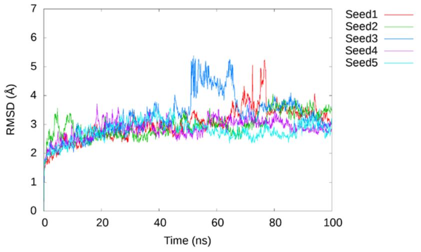 | 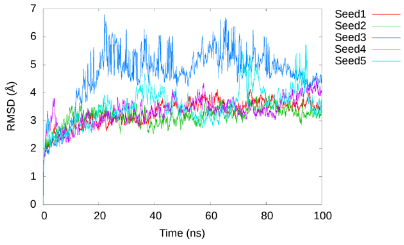 | 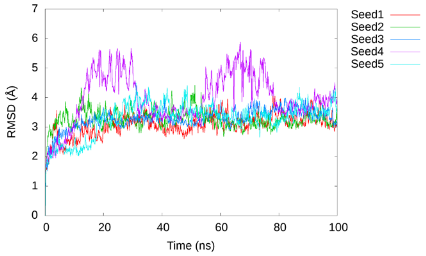 |

# Analysis of Drugs - Amino Acid Interactions:

**Table S11.** Hydrogen bonding percent occurrences and pairwise decomposition energies for apo and ligand-bound complexes. Listed below are hydrogen bonding interactions between RBD and hACE2 residues. These interactions have average percent occurrences greater than 5% for at least one of the complexes. Then for each hydrogen bond, the corresponding average pairwise decomposition energy is listed in kcal/mol with the standard deviation. A dash is listed for hydrogen bonds that were not greater than 5% or not present for the specific complex. **A.** For the interaction, RBD Ala475 - hACE2 Ser19 (HX-N), HX-N refers to a hydrogen bond taking place

between residue Ala475 and hydrogens (where X = 1, 2, or 3) on a nitrogen present on the amino acid Ser. This previously mentioned interaction differs from the first RBD Ala475 - hACE2 Ser19 interaction in that the first interaction is between residue Ala475 and oxygen on Ser.

| | | Apo | | Fosinopril Pose 1 | | Fosinoprilat Pose 2 | | Fosinoprilat Pose 3 | | Lisinopril Pose 1 | |
|---|---|---|---|---|---|---|---|---|---|---|---|
| RBD Residue | hACE2 Residue | Hydrogen Bonding Percent Occurrence (avg.) | Pairwise Decomposition (avg. ± std. dev.) (kcal/mol) | Hydrogen Bonding Percent Occurrence (avg.) | Pairwise Decomposition (avg. ± std. dev.) (kcal/mol) | Hydrogen Bonding Percent Occurrence (avg.) | Pairwise Decomposition (avg. ± std. dev.) (kcal/mol) | Hydrogen Bonding Percent Occurrence (avg.) | Pairwise Decomposition (avg. ± std. dev.) (kcal/mol) | Hydrogen Bonding Percent Occurrence (avg.) | Pairwise Decomposition (avg. ± std. dev.) (kcal/mol) |
| Asn487 | Tyr83 | 69.56 | -2.89 ± 0.96 | 61.42 | -3.11 ± 0.84 | 46.94 | -2.97 ± 0.89 | 65.66 | -2.98 ± 0.88 | 66.34 | -3.13 ± 0.80 |
| | Gln24 | 19.62 | -2.87 ± 0.93 | 16.20 | -2.93 ± 0.87 | 15.08 | -2.79 ± 0.98 | 20.84 | -2.88 ± 0.89 | 19.08 | -2.95 ± 0.89 |
| Gly502 | Lys353 | 66.80 | -1.63 ± 0.32 | 49.88 | -1.48 ± 0.34 | 48.04 | -1.57 ± 0.31 | 60.62 | -1.61 ± 0.33 | 52.70 | -1.47 ± 0.31 |
| Tyr449 | Asp38 | 59.72 | -3.59 ± 2.01 | 46.48 | -3.33 ± 2.07 | 56.04 | -4.53 ± 1.24 | 53.06 | -3.63 ± 1.95 | 60.98 | -4.06 ± 1.75 |
| | Gln42 | - | -0.76 ± 0.92 | - | -0.93 ± 1.00 | - | -0.81 ± 0.88 | 7.74 | -0.78 ± 1.02 | 7.70 | -0.99 ± 1.02 |
| Lys417 | Asp30 | 57.81 | -6.52 ± 2.94 | 35.32 | -4.86 ± 3.76 | 41.52 | -5.62 ± 3.51 | 53.18 | -6.79 ± 3.06 | 61.14 | -9.38 ± 3.50 |
| Gln493 | Glu35 | 52.44 | -4.50 ± 1.80 | 50.08 | -4.69 ± 1.55 | 39.28 | -4.54 ± 1.73 | 55.10 | -4.87 ± 1.57 | 51.26 | -4.77 ± 1.60 |
| | Lys31 | 36.74 | -4.13 ± 1.95 | 28.48 | -4.31 ± 1.80 | 22.80 | -3.87 ± 2.06 | 34.48 | -3.80 ± 2.02 | 30.24 | -4.09 ± 2.06 |
| | His34 | - | -2.12 ± 1.23 | - | -2.32 ± 1.45 | 8.04 | -2.57 ± 1.29 | 8.80 | -2.57 ± 1.28 | 5.82 | -2.10 ± 1.47 |
| Tyr505 | Glu37 | 44.40 | -2.86 ± 2.38 | 33.56 | -2.43 ± 2.21 | 25.54 | -2.46 ± 2.28 | 54.64 | -3.55 ± 2.19 | 61.88 | -4.24 ± 1.97 |
| | Arg393 | - | -1.42 ± 1.23 | - | -0.56 ± 0.63 | - | -0.73 ± 0.75 | - | -0.72 ± 0.73 | 7.00 | -1.03 ± 1.08 |

| | | | | | | | | | | | |
|---|---|---|---|---|---|---|---|---|---|---|---|
| | Ala386 | 10.08 | -0.50 ± 0.82 | - | -0.27 ± 0.27 | - | -0.33 ± 0.48 | - | -0.18 ± 0.15 | - | -0.18 ± 0.13 |
| Thr500 | Asp355 | 42.19 | -4.70 ± 2.44 | 47.24 | -5.68 ± 2.34 | 32.80 | -5.29 ± 2.32 | 47.10 | -5.30 ± 2.33 | 54.10 | -5.75 ± 2.07 |
| | Tyr41 | 29.68 | -1.26 ± 1.46 | 17.90 | -0.71 ± 1.32 | 21.38 | -1.02 ± 1.38 | 20.76 | -0.95 ± 1.38 | 14.28 | -0.48 ± 1.13 |
| Gln498 | Lys353 | 34.90 | -3.86 ± 3.30 | - | -0.80 ± 2.19 | 24.18 | -4.14 ± 3.03 | 45.18 | -4.42 ± 3.23 | 19.08 | -1.81 ± 3.13 |
| | Asp38 | 19.52 | -1.68 ± 2.27 | - | -0.69 ± 1.44 | 12.28 | -1.95 ± 2.37 | 27.72 | -2.13 ± 2.27 | - | -0.68 ± 1.45 |
| | Gln42 | - | -0.41 ± 1.12 | 11.18 | -1.18 ± 1.49 | - | -0.34 ± 1.20 | - | -0.17 ± 0.68 | - | -0.47 ± 1.15 |
| Gly496 | Lys353 | 26.47 | -3.25 ± 1.77 | 6.80 | -2.23 ± 1.50 | 18.56 | -3.46 ± 1.62 | 18.56 | -3.38 ± 1.47 | 7.12 | -2.40 ± 1.57 |
| Tyr453 | His34 | 24.98 | -2.07 ± 1.05 | 16.92 | -1.76 ± 1.16 | 35.28 | -2.38 ± 1.16 | 33.30 | -2.48 ± 1.21 | 10.32 | -1.33 ± 0.86 |
| Ala475 | Ser19 | 17.37 | -0.73 ± 1.29 | 28.22 | -1.35 ± 1.35 | 17.48 | -1.53 ± 1.51 | 26.48 | -1.54 ± 1.39 | 15.64 | -1.49 ± 1.61 |
| | Gln24 | 11.76 | -1.93 ± 1.18 | - | -1.52 ± 0.88 | 7.60 | -1.71 ± 1.03 | - | -1.52 ± 1.00 | 9.80 | -1.71 ± 1.05 |
| | Ser19 (HX-N) | - | 0.73 ± 1.29 | - | -1.35 ± 1.35 | 6.60 | -1.53 ± 1.51 | - | -1.54 ± 1.39 | 5.38 | -1.49 ± 1.61 |
| Ser494 | His34 | 9.93 | -1.05 ± 1.07 | - | -0.23 ± 0.28 | - | -0.40 ± 0.64 | - | -0.43 ± 0.71 | - | -0.32 ± 0.45 |
| Gly446 | Gln42 | 9.02 | -0.71 ± 0.97 | 7.06 | -0.96 ± 1.01 | 8.42 | -0.92 ± 1.11 | 13.20 | -0.74 ± 1.10 | 13.60 | -0.98 ± 1.14 |
| Tyr495 | Lys353 | 7.75 | -0.31 ± 2.00 | 16.00 | -1.44 ± 2.39 | 5.54 | -0.96 ± 2.34 | 13.44 | -0.81 ± 2.33 | 17.78 | -2.04 ± 2.27 |
| Tyr489 | Tyr83 | - | -0.11 ± 0.38 | 7.10 | -0.24 ± 0.63 | - | -0.10 ± 0.33 | - | -0.40 ± 0.20 | 10.46 | -0.33 ± 0.69 |

**Table S12.** Average pairwise decomposition energies for interactions involving fosinopril. Additionally, the average van der Waals and electrostatic energies are included, as well as the standard deviation and standard error for all energies. All values are reported in units of kcal/mol. Interactions were selected if pairwise decomposition was less than -1 kcal/mol.

| Residue | van der Waals Avg. ± Std. Dev. (Std. Error) | Electrostatic Avg. ± Std. Dev. (Std. Error) | Pairwise Decomposition Avg. ± Std. Dev. (Std. Error) |
|---|---|---|---|
| RBD Arg408 | -0.36 ± 0.94 (0.04) | -38.32 ± 16.85 (0.75) | -8.27 ± 5.82 (0.26) |
| RBD Arg403 | -0.95 ± 1.10 (0.05) | -24.51 ± 11.76 (0.53) | -7.17 ± 6.01 (0.27) |
| hACE2 His34 | -1.60 ± 1.34 (0.06) | -0.67 ± 0.79 (0.04) | -2.95 ± 2.38 (0.11) |
| hACE2 Ala387 | -1.39 ± 0.68 (0.03) | -0.14 ± 0.82 (0.04) | -2.66 ± 1.25 (0.06) |
| hACE2 Pro389 | -0.97 ± 0.69 (0.03) | -0.19 ± 0.34 (0.02) | -1.87 ± 1.36 (0.06) |
| hACE2 Arg559 | -0.53 ± 0.90 (0.04) | -14.58 ± 8.97 (0.40) | -1.73 ± 3.33 (0.15) |
| hACE2 Gln388 | -0.93 ± 0.79 (0.04) | -0.09 ± 0.95 (0.04) | -1.52 ± 1.41 (0.06) |
| RBD Tyr505 | -0.89 ± 0.75 (0.03) | 0.25 ± 0.54 (0.02) | -1.50 ± 1.38 (0.06) |
| RBD Lys417 | -0.72 ± 0.73 (0.03) | -13.96 ± 5.05 (0.23) | -1.42 ± 1.41 (0.06) |
| hACE2 Asn33 | -0.64 ± 0.52 (0.02) | -0.14 ± 0.44 (0.02) | -1.19 ± 0.98 (0.04) |

**Table S13**. Average pairwise decomposition energies for interactions involving fosinoprilat pose 2. Additionally, the average van der Waals and electrostatic energies are included, as well as the standard deviation and standard error for all energies. All values are reported in units of kcal/mol. Interactions were selected if pairwise decomposition was less than -1 kcal/mol.

| Residue | van der Waals Avg. ± Std. Dev. (Std. Error) | Electrostatic Avg. ± Std. Dev. (Std. Error) | Pairwise Decomposition Avg. ± Std. Dev. (Std. Error) |
|---|---|---|---|
| RBD Arg403 | -1.03 ± 0.90 (0.04) | -59.07 ± 13.69 (0.61) | -14.92 ± 7.99 (0.36) |
| RBD Arg408 | -0.66 ± 1.24 (0.06) | -55.46 ± 14.84 (0.66) | -8.20 ± 6.84 (0.31) |
| RBD Lys417 | -0.25 ± 0.48 (0.02) | -52.90 ± 17.06 (0.76) | -5.37 ± 6.67 (0.30) |
| hACE2 His34 | -2.24 ± 0.66 (0.03) | -3.80 ± 4.26 (0.19) | -4.42 ± 2.31 (0.10) |
| hACE2 Lys353 | -0.63 ± 0.40 (0.02) | -26.31 ± 2.97 (0.13) | -2.52 ± 1.66 (0.07) |
| RBD Tyr505 | -1.12 ± 0.49 (0.02) | 0.24 ± 1.76 (0.08) | -1.89 ± 1.06 (0.05) |
| hACE2 Asn33 | -0.76 ± 0.47 (0.02) | -2.15 ± 1.98 (0.09) | -1.79 ± 1.56 (0.07) |

| | | | |
|---|---|---|---|
| hACE2 Gln388 | -0.89 ± 0.74 (0.03) | 2.11 ± 1.51 (0.07) | -1.72 ± 1.41 (0.06) |
| hACE2 Pro389 | -1.01 ± 0.71 (0.03) | -1.91 ± 1.13 (0.05) | -1.71 ± 1.49 (0.07) |
| hACE2 Ala387 | -0.90 ± 0.62 (0.03) | 2.95 ± 1.15 (0.05) | -1.65 ± 1.19 (0.05) |
| hACE2 Glu37 | -1.07 ± 0.37 (0.02) | 31.06 ± 3.44 (0.15) | -1.65 ± 0.69 (0.03) |
| hACE2 Arg393 | -0.36 ± 0.23 (0.01) | -35.61 ± 5.64 (0.25) | -1.56 ± 1.87 (0.08) |
| Glycan | -0.65 ± 0.67 (0.03) | -6.74 ± 2.96 (0.13) | -1.41 ± 1.71 (0.08) |
| RBD Tyr495 | -0.58 ± 0.30 (0.01) | -1.49 ± 0.76 (0.03) | -1.01 ± 0.46 (0.02) |

**Table S14.** Average pairwise decomposition energies for interactions involving fosinoprilat pose 3. Additionally, the average van der Waals and electrostatic energies are included, as well as the standard deviation and standard error for all energies. All values are reported in units of kcal/mol. Interactions were selected if pairwise decomposition was less than -1 kcal/mol.

| Residue | van der Waals Avg. ± Std. Dev. (Std. Error) | Electrostatic Avg. ± Std. Dev. (Std. Error)) | Pairwise Decomposition Avg. ± Std. Dev. (Std. Error) |
|---|---|---|---|
| RBD Arg408 | -0.14 ± 1.05 (0.05) | -64.27 ± 17.25 (0.77) | -11.11 ± 7.99 (0.36) |
| RBD Arg403 | -1.03 ± 0.83 (0.04) | -45.75 ± 15.27 (0.68) | -7.28 ± 7.42 (0.33) |
| RBD Lys417 | -0.35 ± 0.47 (0.02) | -46.87 ± 15.89 (0.71) | -4.43 ± 6.42 (0.29) |
| hACE2 His34 | -1.34 ± 1.04 (0.05) | -4.93 ± 4.71 (0.21) | -3.86 ± 3.16 (0.14) |
| Glycan | -0.76 ± 0.74 (0.03) | -8.61 ± 7.00 (0.31) | -3.25 ± 4.83 (0.22) |
| hACE2 Pro389 | -1.20 ± 0.85 (0.04) | -1.35 ± 0.60 (0.03) | -2.11 ± 1.54 (0.07) |
| hACE2 Ala387 | -0.93 ± 0.67 (0.03) | 1.78 ± 1.10 (0.05) | -1.74 ± 1.26 (0.06) |
| hACE2 Asn33 | -0.74 ± 0.42 (0.02) | -1.58 ± 1.65 (0.07) | -1.66 ± 0.91 (0.04) |
| hACE2 Gln388 | -0.91 ± 0.69 (0.03) | 1.33 ± 1.28 (0.06) | -1.66 ± 1.32 (0.06) |
| hACE2 Lys26 | -0.07 ± 0.26 (0.01) | -29.26 ± 12.81 (0.57) | -1.50 ± 4.23 (0.19) |
| hACE2 Arg393 | -0.41 ± 0.29 (0.01) | -30.21 ± 4.10 (0.18) | -1.43 ± 1.11 (0.05) |
| RBD Gln409 | -0.33 ± 0.40 (0.02) | -4.01 ± 2.53 (0.11) | -1.21 ± 1.47 (0.07) |

| | | | |
|---|---|---|---|
| RBD Tyr505 | -0.75 ± 0.64 (0.03) | 0.61 ± 0.71 (0.03) | -1.11 ± 0.97 (0.04) |

**Table S15.** Average pairwise decomposition energies for interactions involving lisinopril. Additionally, the average van der Waals and electrostatic energies are included, as well as the standard deviation and standard error for all energies. All values are reported in units of kcal/mol. Interactions were selected if pairwise decomposition was less than -1 kcal/mol.

| Residue | van der Waals Avg. ± Std. Dev. (Std. Error) | Electrostatic Avg. ± Std. Dev. (Std. Error) | Pairwise Decomposition Avg. ± Std. Dev. (Std. Error) |
|---|---|---|---|
| RBD Arg408 | -0.13 ± 0.78 (0.03) | -35.15 ± 13.91 (0.62) | -6.96 ± 5.97 (0.27) |
| RBD Lys417 | -0.44 ± 0.58 (0.03) | -27.15 ± 12.77 (0.57) | -5.29 ± 5.98 (0.27) |
| hACE2 Asp30 | -1.84 ± 1.30 (0.06) | 15.91 ± 7.43 (0.33) | -4.46 ± 4.63 (021) |
| hACE2 His34 | -2.03 ± 1.38 (0.06) | -2.71 ± 2.46 (0.11) | -4.12 ± 3.07 (0.14) |
| hACE2 Asn33 | -1.96 ± 1.08 (0.05) | -0.97 ± 1.97 (0.09) | -3.50 ± 2.69 (0.12) |
| hACE2 Glu37 | -0.74 ± 0.91 (0.04) | 10.43 ± 3.15 (0.14) | -2.78 ± 3.57 (0.16) |
| hACE2 Pro389 | -1.52 ± 0.74 (0.03) | -0.48 ± 0.51 (0.02) | -2.50 ± 1.10 (0.05) |
| Glycan | -1.02 ± 0.61 (0.03) | -3.69 ± 1.46 (0.07) | -2.16 ± 1.35 (0.06) |
| RBD Arg403 | -0.79 ± 0.50 (0.02) | -18.09 ± 4.42 (0.20) | -2.00 ± 1.59 (0.07) |
| hACE2 Leu29 | -0.81 ± 0.55 (0.02) | -0.24 ± 0.44 (0.02) | -1.62 ± 1.12 (0.05) |
| hACE2 Lys26 | -0.73 ± 0.59 (0.03) | -13.19 ± 2.69 (0.12) | -1.62 ± 1.35 (0.06) |
| hACE2 Gln388 | -0.82 ± 0.85 (0.04) | 0.46 ± 1.09 (0.05) | -1.52 ± 1.63 (0.07) |
| hACE2 Ala387 | -0.80 ± 0.82 (0.04) | 1.34 ± 1.02 (0.05) | -1.47 ± 1.62 (0.07) |
| hACE2 Gln96 | -0.45 ± 0.38 (0.02) | -1.03 ± 1.36 (0.06) | -1.45 ± 1.45 (0.06) |
| RBD Asp405 | -0.70 ± 0.44 (0.02) | 19.04 ± 4.23 (0.19) | -1.05 ± 1.01 (0.05) |

**Fosinopril**

**Table S16.** SARS-CoV-2 RBD - hACE2 fosinopril pairwise decomposition energies. Listed in the table are the average pairwise decomposition energies for interactions between RBD and

hACE2 residues when fosinopril is present in the interface. Additionally, the average van der Waals and electrostatic energies are included, as well as the standard deviation and standard error for all energies. All values are reported in units of kcal/mol. Interactions were selected if pairwise decomposition was less than -2 kcal/mol.

| RBD Residue | hACE2 Residue | van der Waals Avg. ± Std. Dev. (Std. Error) | Electrostatic Avg. ± Std. Dev. (Std. Error) | Pairwise Decomposition Avg. ± Std. Dev. (Std. Error) |
|---|---|---|---|---|
| Thr500 | Asp355 | -0.54 ± 0.60 (0.03) | -4.48 ± 3.03 (0.14) | -5.68 ± 2.34 (0.10) |
| Asn501 | Lys353 | -1.52 ± 0.31 (0.01) | -3.72 ± 1.79 (0.08) | -5.06 ± 1.20 (0.05) |
| Tyr505 | Lys353 | -2.16 ± 0.39 (0.02) | -1.39 ± 0.76 (0.03) | -4.94 ± 0.55 (0.02) |
| Lys417 | Asp30 | 0.12 ± 0.61 (0.03) | -38.31 ± 10.10 (0.45) | -4.86 ± 3.76 (0.17) |
| Gln493 | Glu35 | -0.44 ± 0.57 (0.03) | -5.03 ± 2.36 (0.11) | -4.69 ± 1.55 (0.07) |
| Gln493 | Lys31 | -0.21 ± 0.52 (0.02) | -7.48 ± 2.53 (0.11) | -4.31 ± 1.80 (0.08) |
| Tyr449 | Asp38 | 0.10 ± 0.77 (0.03) | -5.72 ± 4.19 (0.19) | -3.33 ± 2.07 (0.09) |
| Asn487 | Tyr83 | 0.02 ± 0.62 (0.03) | -3.72 ± 1.21 (0.05) | -3.11 ± 0.84 (0.04) |
| Asn487 | Gln24 | -0.91 ± 0.40 (0.02) | -1.33 ± 1.26 (0.06) | -2.93 ± 0.87 (0.04) |
| Phe486 | Met82 | -1.27 ± 0.41 (0.02) | -0.04 ± 0.27 (0.01) | -2.59 ± 0.77 (0.03) |
| Tyr505 | Glu37 | -0.19 ± 0.54 (0.02) | -4.30 ± 3.80 (0.17) | -2.43 ± 2.21 (0.10) |
| Gln493 | His34 | -0.91 ± 0.31 (0.01) | -1.82 ± 1.24 (0.06) | -2.32 ± 1.45 (0.06) |
| Gly496 | Lys353 | -0.21 ± 0.43 (0.02) | -3.81 ± 3.03 (0.14) | -2.23 ± 1.50 (0.07) |
| Gln498 | Tyr41 | -1.02 ± 0.37 (0.02) | -0.05 ± 0.59 (0.03) | -2.21 ± 0.70 (0.03) |
| Asn501 | Tyr41 | -0.66 ± 0.30 (0.01) | -0.42 ± 0.63 (0.03) | -2.01 ± 1.03 (0.05) |

**Table S17.** Hydrogen bonds and percent occurrence for fosinopril. Hydrogen bonds involving fosinopril with a percent occurrence greater than 5% are listed.

| Acceptor | Donor | Hydrogen Bonding Percent Occurrence (avg.) |
|---|---|---|
| Fosinopril @ O2 | RBD - Arg403 @ HH12-NH1, HH22-NH2 | 54.94 |
| Fosinopril @ O6 | RBD - Arg408 @ HH11-NH1, HH12-NH1, HH21-NH2, HH22-NH2 | 22.84 |
| Fosinopril @ O7 | RBD - Arg408 @ HH11-NH1, HH12-NH1, HH21-NH2, HH22-NH2 | 21.84 |
| Fosinopril @ O5 | RBD - Gln409 @ HE22-NE2 | 8.64 |
| Fosinopril @ O7 | RBD - Arg408 @ HE-NE | 8.42 |
| Fosinopril @ O6 | RBD - Arg408 @ HE-NE | 8.06 |
| Fosinopril @ O1 | RBD - Arg408 @ HH11-NH1, HH12-NH1, HH22-NH2 | 8.06 |

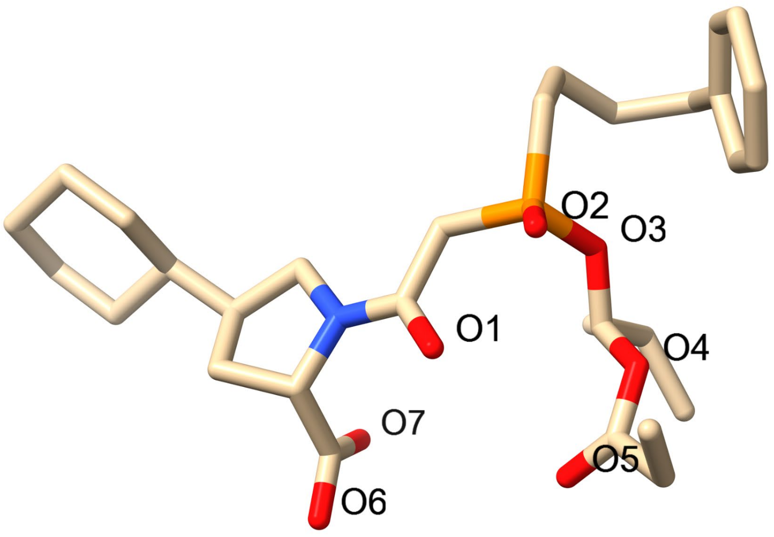


**Figure S6.** Fosinopril with oxygen labels.

Based on hydrogen bonding analysis, fosinopril disrupts the interactions RBD Ala475 - hACE2 Gln24, RBD Gln498 - hACE2 Lys353, and RBD Gln498 - hACE2 Asp38. The disruption of the interactions RBD Gln498 - hACE2 Lys353 and RBD Gln498 - hACE2 Asp38 are further supported by the increase in pairwise decomposition energy of these interactions. Also, a notable electrostatic interaction occurs between hACE2 Lys353 and fosinopril with an average electrostatic energy of -10.01 (0.09) kcal/mol and an average pairwise decomposition energy of -0.64 (0.05) kcal/mol, further supporting that the presence of fosinopril in the interface disrupts the interaction between RBD Gln498 and hACE2 Lys353. There is a prominent decrease (difference greater than 15%) in the hydrogen bond occurrence for the following interactions when fosinopril is present in the interface: RBD Gly502 - hACE2 Lys353, RBD Lys417 - hACE2 Asp30, and RBD Gly496 - hACE2 Lys353. Notably, Lys417 and fosinopril form a significant electrostatic interaction with an average electrostatic energy of -13.96 (0.23) kcal/mol and an average pairwise decomposition energy of -1.42 (0.06) kcal/mol.

Table S16 contains the pairwise decomposition energies for interactions between the RBD and hACE2 with energies less than -2 kcal/mol when fosinopril is bound in the interface. There is a notable decrease in the average pairwise decomposition energy of the interactions RBD Gln498 - hACE2 Gln42 and RBD Thr500 - hACE2 Asp355 when fosinopril is present. There is an increase in the hydrogen bond occurrence of the interaction RBD Gln498 - hACE2 Gln42. This information suggests that the presence of fosinopril in the interface enhances these two interactions, demonstrating that the drug not only inhibits but, to a lesser degree, strengthens the interaction between the RBD and hACE2.

Table S17 contains the hydrogen bonding interactions involving fosinopril. It is important to note that three significant hydrogen bonds between oxygen atoms on fosinopril and RBD residues, such as Arg403 and Arg408, have a percent occurrence above 20%. Additionally, the average pairwise decomposition energies of the interaction between the RBD residues Arg403 and Arg408 with fosinopril are -7.17 (0.27) and -8.27 (0.26) kcal/mol, respectively. Interestingly, the significant hydrogen bonding interactions that form between fosinopril and the RBD do not involve RBD residues that are present in the previously mentioned disrupted, diminished, or enhanced interactions. The formation of these interactions, along with other electrostatic or van der Waals interactions, cause the previous interactions to be unfavorable or more favorable.

**Fosinoprilat**

**Table S18.** Hydrogen bonds and percent occurrence for fosinoprilat pose 2. Hydrogen bonds involving fosinoprilat in pose 2 with a percent occurrence greater than 5% are listed.

| Acceptor | Donor | Hydrogen Bonding Percent Occurrence (avg.) |
|---|---|---|
| Fosinoprilat @ O5 | RBD - Arg408 @ HH11-NH1, HH12-NH1, HH22-NH2 | 22.36 |
| Fosinoprilat @ O3 | RBD - Arg403 @ HH12-NH1, HH22-NH2 | 22.00 |
| Fosinoprilat @ O2 | RBD - Arg403 @ HH12-NH1, HH22-NH2 | 20.88 |
| Fosinoprilat @ O4 | RBD - Arg408 @ HH11-NH1, HH12-NH1, HH22-NH2 | 20.60 |
| Fosinoprilat @ O2 | RBD - Lys417 @ HZ1, HZ2, HZ3-NZ | 11.44 |

**Table S19.** SARS-CoV-2 RBD - hACE2 fosinoprilat pose 2 pairwise decomposition energies. Listed in the table are the average pairwise decomposition energies for interactions between RBD and hACE2 residues when fosinoprilat in pose 2 is present in the interface. Additionally, the average van der Waals and electrostatic energies are included, as well as the standard deviation and standard error for all energies. All values are reported in units of kcal/mol Interactions were selected if pairwise decomposition was less than -2 kcal/mol.

| RBD Residue | hACE2 Residue | van der Waals Avg. ± Std. Dev. (Std. Error) | Electrostatic Avg. ± Std. Dev. (Std. Error) | Pairwise Decomposition Avg. ± Std. Dev. (Std. Error) |
|---|---|---|---|---|
| Lys417 | Asp30 | 0.11 ± 0.64 (0.03) | -41.11 ± 9.36 (0.42) | -5.62 ± 3.51 (0.16) |
| Thr500 | Asp355 | -0.63 ± 0.64 (0.03) | -3.74 ± 3.03 (0.14) | -5.29 ± 2.32 (0.10) |
| Tyr505 | Lys353 | -2.06 ± 0.38 (0.02) | -1.68 ± 0.77 (0.03) | -4.78 ± 0.60 (0.03) |
| Asn501 | Lys353 | -1.60 ± 0.30 (0.01) | -3.60 ± 1.67 (0.07) | -4.61 ± 1.24 (0.06) |
| Gln493 | Glu35 | -0.43 ± 0.60 (0.03) | -4.75 ± 2.61 (0.12) | -4.54 ± 1.73 (0.08) |
| Tyr449 | Asp38 | 0.31 ± 0.70 (0.03) | -8.25 ± 2.15 (0.10) | -4.53 ± 1.24 (0.06) |
| Gln498 | Lys353 | -0.18 ± 0.46 (0.02) | -5.42 ± 4.37 (0.20) | -4.14 ± 3.03 (0.14) |

| | | | | |
|---|---|---|---|---|
| Gln493 | Lys31 | -0.19 ± 0.45 (0.02) | -6.88 ± 3.06 (0.14) | -3.87 ± 2.06 (0.09) |
| Gly496 | Lys353 | 0.07 ± 0.55 (0.02) | -5.52 ± 2.23 (0.10) | -3.46 ± 1.62 (0.07) |
| Asn487 | Tyr83 | -0.07 ±` 0.53 (0.02) | -3.35 ± 1.47 (0.07) | -2.97 ± 0.89 (0.04) |
| Asn487 | Gln24 | -0.88 ± 0.41 (0.02) | -1.36 ± 1.23 (0.05) | -2.80 ± 0.98 (0.04) |
| Phe486 | Met82 | -1.30 ± 0.38 (0.01) | -0.008 ± 0.270 (0.012) | -2.57 ± 0.68 (0.03) |
| Gln493 | His34 | -0.85 ± 0.44 (0.02) | -1.90 ± 1.45 (0.06) | -2.57 ± 1.29 (0.06) |
| Tyr505 | Glu37 | -0.29 ± 0.51 (0.02) | -3.73 ± 3.98 (0.18) | -2.46 ± 2.28 (0.10) |
| Tyr453 | His34 | -0.30 ± 0.55 (0.02) | -2.19 ± 1.38 (0.06) | -2.38 ± 1.16 (0.05) |
| Asn501 | Tyr41 | -0.74 ± 0.25 (0.01) | -0.59 ± 0.63 (0.03) | -2.37 ± 1.03 (0.05) |
| Phe486 | Tyr83 | -0.99 ± 0.38 (0.02) | -0.39 ± 0.25 (0.01) | -2.09 ± 0.65 (0.03) |

**Table S20.** Hydrogen bonds and percent occurrence for fosinoprilat pose 3. Hydrogen bonds involving fosinoprilat in pose 3 with a percent occurrence greater than 5% are listed.

| Acceptor | Donor | Hydrogen Bonding Percent Occurrence (avg.) |
|---|---|---|
| Fosinoprilat @ O4 | RBD - Arg408 @ HH11-NH1, HH12-NH1, HH21-NH2, HH22-NH2 | 21.08 |
| Fosinoprilat @ O5 | RBD - Arg408 @ HH11-NH1, HH12-NH1, HH21-NH2, HH22-NH2 | 19.90 |
| Fosinoprilat @ O2 | hACE2 His34 @ HE2-NE2 | 18.14 |
| Fosinoprilat @ O3 | RBD - Arg408 @ HH11-NH1, HH12-NH1, HH22-NH2 | 17.84 |
| Fosinoprilat @ O2 | RBD - Arg408 @ HH11-NH1, HH12-NH1, HH22-NH2 | 13.52 |

| | | |
|---|---|---|
| Fosinoprilat @ O3 | RBD - Arg403 @ HH12-NH1, HH21-NH2, HH22-NH2 | 12.84 |
| Fosinoprilat @ O2 | RBD - Lys417 @ HZ1, HZ2, HZ3-NZ | 9.20 |
| Fosinoprilat @ O2 | hACE2 - Glycan @ H4O-O4 | 8.32 |
| Fosinoprilat @ O2 | hACE2 - Glycan @ H3O-O3 | 8.00 |
| Fosinoprilat @ O3 | hACE2 - Lys26 @ HZ1, HZ2, HZ3-NZ | 5.46 |
| Fosinoprilat @ O3 | RBD - Lys417 @ HZ1, HZ2, HZ3-NZ | 5.38 |
| Fosinoprilat @ O1 | RBD - Arg408 @ HH11-NH1, HH12-NH1, HH22-NH2 | 5.20 |

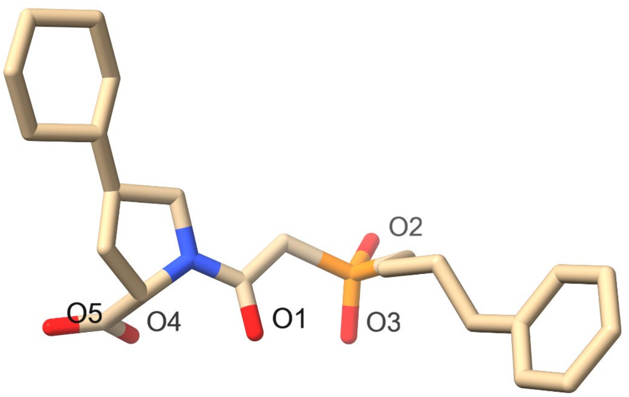


**Figure S7.** Fosinoprilat with oxygen labels.

**Table S21.** SARS-CoV-2 RBD - hACE2 fosinoprilat pose 3 pairwise decomposition Energies. Listed in the table are the average pairwise decomposition energies for interactions between RBD and hACE2 residues when fosinoprilat in pose 3 is present in the interface. Additionally, the average van der Waals and electrostatic energies are included, as well as the standard deviation and standard error for all energies. All values are reported in units of kcal/mol. Interactions were selected if pairwise decomposition was less than -2 kcal/mol.

| RBD Residue | hACE2 Residue | van der Waals Avg. ± Std. Dev. (Std. Error) | Electrostatic Avg. ± Std. Dev. (Std. Error) | Pairwise Decomposition Avg. ± Std. Dev. (Std. Error) |
|---|---|---|---|---|
| Lys417 | Asp30 | 0.12 ± 0.62 (0.03) | -43.65 ± 7.77 (0.35) | -6.79 ± 3.06 (0.14) |
| Thr500 | Asp355 | -0.65 ± 0.60 (0.03) | -3.80 ± 3.05 (0.14) | -5.30 ± 2.33 (0.10) |
| Tyr505 | Lys353 | -2.24 ± 0.33 (0.01) | -1.51 ± 0.79 (0.04) | -5.08 ± 0.52 (0.02) |
| Asn501 | Lys353 | -1.64 ± 0.28 (0.01) | -4.15 ± 1.71 (0.08) | -5.08 ± 1.36 (0.06) |
| Gln493 | Glu35 | -0.43 ± 0.61 (0.03) | -5.16 ± 2.43 (0.11) | -4.87 ± 1.57 (0.07) |
| Gln498 | Lys353 | -0.17 ± 0.48 (0.02) | -5.35 ± 4.80 (0.21) | -4.42 ± 3.23 (0.14) |
| Gln493 | Lys31 | -0.15 ± 0.53 (0.02) | -6.77 ± 3.24 (0.14) | -3.80 ± 2.02 (0.09) |
| Tyr449 | Asp38 | 0.09 ± 0.70 (0.03) | -6.39 ± 3.82 (0.17) | -3.63 ± 1.95 (0.09) |
| Tyr505 | Glu37 | -0.16 ± 0.71 (0.03) | -5.69 ± 3.76 (0.17) | -3.55 ± 2.19 (0.10) |
| Gly496 | Lys353 | 0.09 ± 0.53 (0.02) | -5.37 ± 2.29 (0.10) | -3.38 ± 1.47 (0.07) |
| Asn487 | Tyr83 | -0.05 ± 0.53 (0.02) | -3.53 ± 1.25 (0.06) | -2.97 ± 0.88 (0.04) |
| Asn487 | Gln24 | -0.89 ± 0.37 (0.02) | -1.43 ± 1.13 (0.05) | -2.88 ± 0.89 (0.04) |
| Gln493 | His34 | -0.82 ± 0.43 (0.02) | -1.93 ± 1.52 (0.07) | -2.57 ± 1.28 (0.06) |
| Tyr453 | His34 | -0.32 ± 0.57 (0.03) | -2.10 ± 1.57 (0.07) | -2.48 ± 1.21 (0.05) |
| Phe486 | Met82 | -1.20 ± 0.41 (0.02) | 0.02 ± 0.28 (0.01) | -2.41 ± 0.73 (0.03) |
| Asn501 | Tyr41 | -0.73 ± 0.29 (0.01) | -0.51 ± 0.60 (0.03) | -2.21 ± 1.03 (0.05) |
| Gln498 | Asp38 | -0.09 ± 0.31 (0.01) | -2.95 ± 2.47 (0.11) | -2.13 ± 2.27 (0.10) |

Fosinoprilat pose 2 reduces the occurrence of hydrogen bonds, such as RBD Gly502 - hACE2 Lys353, RBD Lys417 - hACE2 Asp30, RBD Asn487 - hACE2 Tyr83, and RBD Tyr505 - hACE2 Glu37, as shown by Table S11 This decrease in hydrogen bond occurrence could be due to the formation of additional interactions between fosinoprilat (pose 2) and the complex. These interactions are detailed in Tables S13 and S18. Notably, the interaction between fosinoprilat (pose 2) and RBD Lys417 can explain the decrease in the percent occurrence of the hydrogen bond between RBD Lys417 and hACE2 Asp30. Specifically, fosinoprilat (pose 2) competes with this interaction by forming a significant hydrogen bond with RBD Lys417. The average pairwise decomposition energy of the interaction between fosinoprilat (pose 2) and RBD

Lys417 is -5.37 (0.30) kcal/mol. Pairwise decomposition analysis supports the decrease in the hydrogen bond occurrence of RBD Gly502 - hACE2 Lys353 and RBD Tyr505 - hACE2 Glu37. Table S13 demonstrates that fosinoprilat (pose 2) forms a significant electrostatic interaction with hACE2 Lys353 and forms significant van der Waals interactions with RBD Tyr505 and hACE2 Glu37. The average pairwise decomposition energies for the residues hACE2 Lys353, RBD Tyr505, and hACE2 Glu37 interaction with fosinoprilat (pose 2) are -2.52 (0.07), -1.89 (0.05), and -1.65 (0.03) kcal/mol, respectively.

Also, fosinoprilat (pose 2) forms significant hydrogen bonds with the RBD residues Arg408 and Arg403; despite this, these RBD residues are not involved in diminished hydrogen bonding interactions. However, these interactions are significant for fosinoprilat (pose 2) to maintain in the interface, given that the average pairwise decomposition energies of the interaction between the fosinoprilat (pose 2) and the RBD residues Arg408 or Arg403 are -8.20 (0.31) and -14.92 (0.36) kcal/mol. Table S19 contains the average pairwise decomposition energies for interactions between the RBD and hACE2 with energies less than -2 kcal/mol when fosinoprilat (pose2) is bound. The only notable increase in the average pairwise decomposition energy was for the interaction RBD Tyr449 - hACE2 Asp38 due to a notable decrease in the average electrostatic energy.

The interaction RBD Ala475 - hACE2 Gln24 is disrupted by fosinoprilat pose 3. Fosinoprilat pose 3 does not decrease the occurrence of hydrogen bonds significant for the RBD - hACE2 interaction; however, Table S20 contains hydrogen bonding interactions between fosinoprilat (pose 3) and the complex with an occurrence above 5%. Most hydrogen bonds involve RBD residues Arg403, Arg408, and Lys417. RBD Arg408 is involved in 5 hydrogen bonds with fosinoprilat (pose 3), and the average pairwise decomposition energy of these interactions is -11.11 (0.36) kcal/mol. This suggests that these interactions are significant for fosinoprilat (pose 3) to maintain its position in the interface. Notably, the phosphate oxygen atoms (O3 and O2) on fosinoprilat (pose 3) can compete with hACE2 Asp30, which participates in a significant hydrogen bond with RBD Lys417. The average pairwise decomposition energies between fosinoprilat (pose 3) and Lys417 and between RBD Lys417 and hACE2 Asp30 are -4.43 (0.29) and -6.79 (0.14) kcal/mol, respectively (Figure SCC and Figure 4). Table S21 contains the average pairwise decomposition energies for interactions between the RBD and hACE2 with energies less than -2 kcal/mol when fosinoprilat (pose3) is bound. Notably, there is an increase in the hydrogen bond occurrences and the average pairwise decomposition energies of the interactions RBD Tyr505 - hACE2 Glu37 and RBD 498 - hACE2 Lys353 when fosinoprilat (pose 3) is bound in the interface.

**Lisinopril**

**Table S22.** Hydrogen Bonds and Percent Occurrence for Lisinopril. Hydrogen bonds involving lisinopril with a percent occurrence greater than 5% are listed.

| Acceptor | Donor | Hydrogen Bonding Percent Occurrence (avg.) |
|---|---|---|
| Lisinopril @ O4 | RBD - Arg408 @ HH11-NH1, HH12-NH1, HH22-NH2 | 28.16 |
| Lisinopril @ O5 | RBD - Arg408 @ HH11-NH1, HH12-NH1, HH22-NH2 | 25.80 |
| hACE2 Glu37 @ OE1, OE2 | Lisinopril @ H21, H22, H23-N2 | 28.58 |
| hACE2 Asp30 @ OD1, OD2 | Lisinopril @ H21, H22, H23-N2 | 26.72 |
| hACE2 Asn33 @ O | Lisinopril @ H21, H22, H23-N2 | 24.68 |
| Lisinopril @ O5 | Lisinopril @ H4-N1 | 20..24 |
| Lisinopril @ O4 | Lisinopril @ H4-N1 | 16.64 |
| hACE2 Asp30 @ O | Lisinopril @ H21, H22, H23-N2 | 16.34 |
| Lisinopril @ O1 | hACE2 Gln96 @ HE22-NE2 | 5.70 |

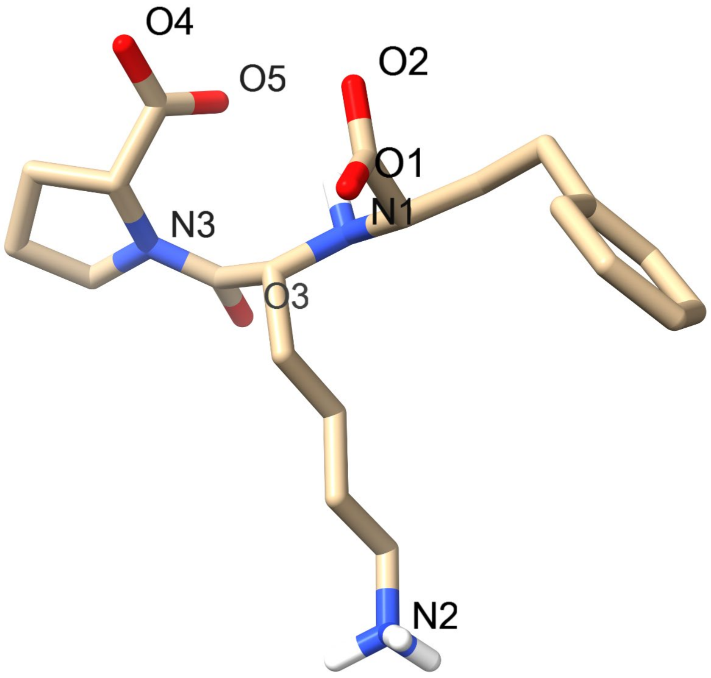


**Figure S8.** Lisinopril with oxygen and nitrogen labels.

**Table S23.** SARS-CoV-2 RBD - hACE2 lisinopril pairwise decomposition energies. Listed in the table are the average pairwise decomposition energies for interactions between RBD and hACE2 residues when lisinopril is present in the interface. Additionally, the average van der Waals and electrostatic energies are included, as well as the standard deviation and standard error for all energies. All values are reported in units of kcal/mol. Interactions were selected if pairwise decomposition was less than -2 kcal/mol.

| RBD Residue | hACE2 Residue | van der Waals Avg. ± Std. Dev. (Std. Error) | Electrostatic Avg. ± Std. Dev. (Std. Error) | Pairwise Decomposition Avg. ± Std. Dev. (Std. Error) |
|---|---|---|---|---|
| Lys417 | Asp30 | 0.39 ± 0.74 (0.03) | -48.71 ± 7.44 (0.33) | -9.38 ± 3.50 (0.16) |
| Thr500 | Asp355 | -0.49 ± 0.65 (0.03) | -4.59 ± 2.65 (0.12) | -5.75 ± 2.07 (0.09) |
| Tyr505 | Lys353 | -2.19 ± 0.37 (0.02) | -1.11 ± 0.75 (0.03) | -5.06 ± 0.46 (0.02) |
| Asn501 | Lys353 | -1.52 ± 0.32 (0.01) | -3.48 ± 1.75 (0.08) | -4.92 ± 1.04 (0.05) |
| Gln493 | Glu35 | -0.50 ± 0.58 (0.03) | -5.15 ± 2.45 (0.11) | -4.77 ± 1.60 (0.07) |
| Tyr505 | Glu37 | 0.03 ± 0.74 (0.03) | -6.93 ± 3.34 (0.15) | -4.24 ± 1.97 (0.09) |
| Gln493 | Lys31 | -0.17 ± 0.52 (0.02) | -7.04 ± 2.95 (0.13) | -4.09 ± 2.06 (0.09) |
| Tyr449 | Asp38 | 0.14 ± 0.73 (0.03) | -7.08 ± 3.55 (0.16) | -4.06 ± 1.75 (0.08) |
| Asn487 | Tyr83 | -0.07 ± 0.55 (0.02) | -3.63 ± 1.28 (0.06) | -3.13 ± 0.80 (0.04) |
| Asn487 | Gln24 | -0.90 ± 0.38 (0.02) | -1.39 ± 1.29 (0.06) | -2.95 ± 0.89 (0.04) |
| Phe486 | Met82 | -1.32 ± 0.43 (0.02) | 0.001 ± 0.289 (0.013) | -2.64 ± 0.76 (0.03) |
| Gly496 | Lys353 | -0.18 ± 0.42 (0.02) | -4.29 ± 2.46 (0.11) | -2.40 ± 1.57 (0.07) |
| Gln498 | Tyr41 | -1.03 ± 0.36 (0.02) | 0.10 ± 0.65 (0.03) | -2.15 ± 0.61 (0.03) |
| Gln493 | His34 | -0.95 ± 0.28 (0.01) | -1.43 ± 1.25 (0.06) | -2.10 ± 1.47 (0.07) |
| Phe486 | Tyr83 | -1.00 ± 0.37 (0.02) | -0.39 ± 0.21 (0.01) | -2.05 ± 0.65 (0.03) |
| Tyr495 | Lys353 | -0.04 ± 0.35 (0.02) | -3.88 ± 4.57 (0.20) | -2.04 ± 2.27 (0.10) |

The interaction RBD Gln498 - hACE2 Asp38 is disrupted by lisinopril, and there is a notable increase in the average pairwise decomposition energy of this interaction. It is possible

that the formation of a van der Waals interaction and a hydrogen bond between lisinopril and hACE2 Glu37, the residue next to hACE2 Asp38, interferes with this interaction. Additionally, lisinopril decreases the percent occurrence of the hydrogen bonding interactions RBD Gln498 - hACE2 Asp38, RBD Gln498 - hACE2 Lys353, and RBD Gly496 - hACE2 Lys353. Similar to the other ligands, the decrease in the hydrogen bond occurrence of these interactions could be due to the formation of new hydrogen bonding interactions involving lisinopril. Table S22 contains the significant hydrogen bonding interactions between lisinopril and the complex; however, these interactions do not involve residues that are present in the previously mentioned disrupted interactions.

Additionally, the percent occurrence of the hydrogen bonds RBD Tyr505 - hACE2 Glu37, RBD Thr500 - hACE2 Asp355, RBD Tyr489 - hACE2 Tyr83, and RBD Tyr495 - hACE2 Lys353 were increased in the lisinopril-bound complex. Table S23 contains the average pairwise decomposition energies for interactions between the RBD and hACE2 with energies less than -2 kcal/mol when lisinopril is bound in the interface. There is a prominent decrease in average pairwise decomposition energies of the following interactions: RBD Tyr505 - hACE2 Glu37, RBD Thr500 - hACE2 Asp355, and RBD Tyr495 - hACE2. This information suggests that lisinopril stabilizes these interactions. Interestingly, a significant interaction between lisinopril and hACE2 Glu37 occurs with an average pairwise decomposition of -2.78 (0.16) kcal/mol. Table S22 details that a hydrogen bond forms between hACE2 Glu37 and the nitrogen of the lysine-like functional group (N2) on lisinopril for 28.58% of the simulation. Also, there is a decrease in the average pairwise decomposition energy of the interaction RBD Lys417 - hACE2 Asp30 when lisinopril is bound in the interface due to the decrease in the average electrostatic energy. Similarly, lisinopril forms significant electrostatic and van der Waals interactions with RBD Lys417 and hACE2 Asp30, respectively. The average pairwise decomposition energies of lisinopril's interaction with RBD Lys417 and hACE2 Asp30 are -5.29 (0.27) and -4.46 (0.21) kcal/mol, respectively. The formation of strong interactions between residues and lisinopril could cause a change in the distance between RBD and hACE2 residues or decrease the RBD and hACE2 interactions' competition.

Further Analysis

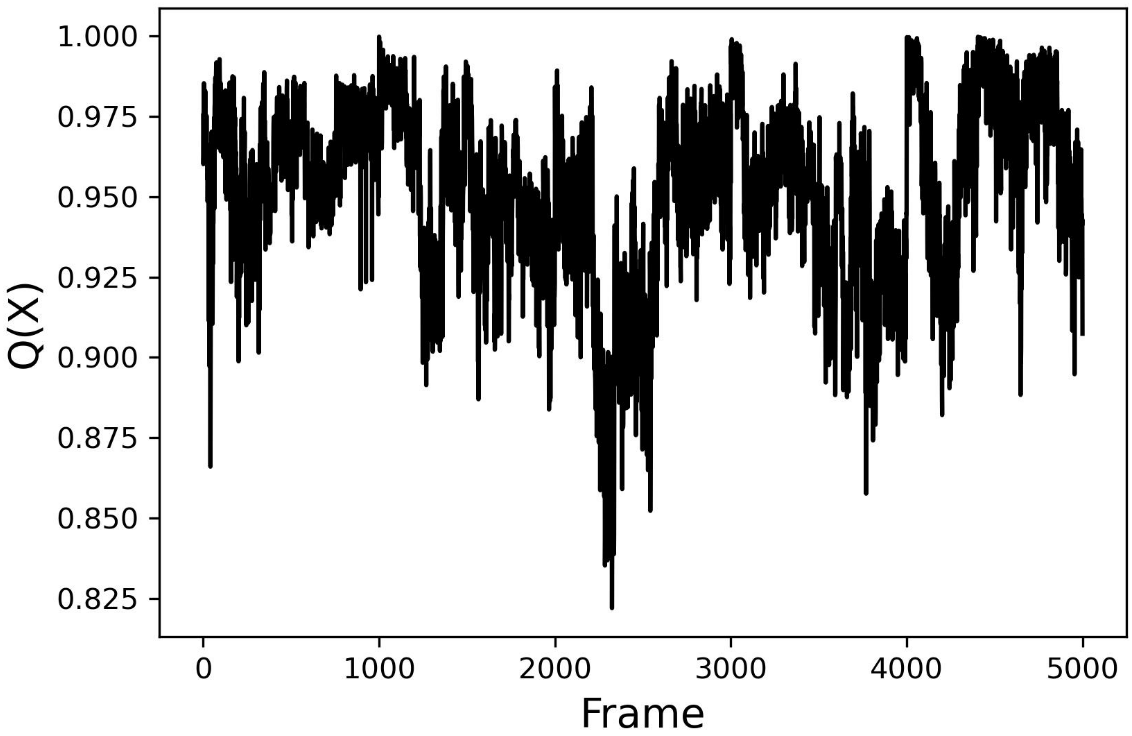


**Figure S9**. Native contact analysis in the interface of fosinopril bound complex 500 ns ensemble. The average fraction of native contacts for the 500 ns ensemble was 0.952 ± 0.027.

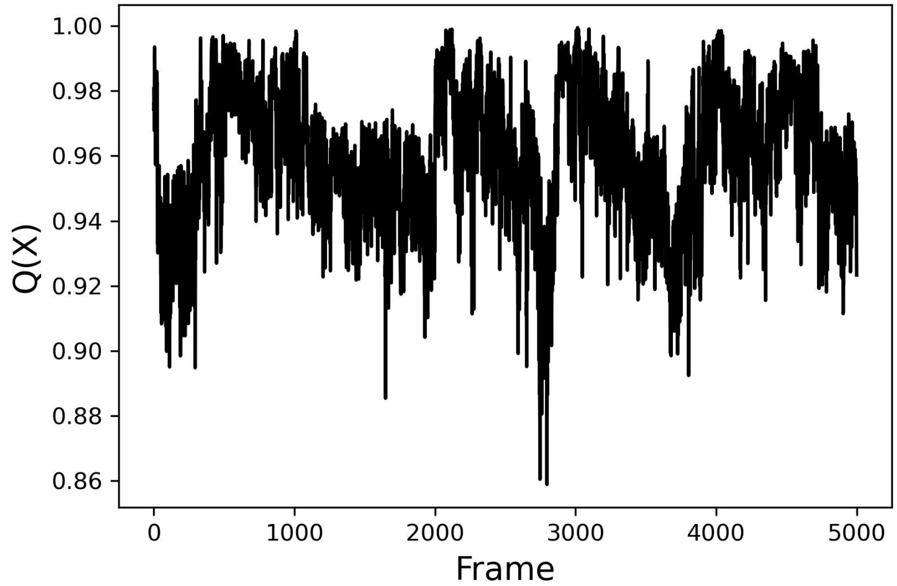

**Figure S10.** Native contact analysis in the interface of fosinoprilat in pose 2 bound complex 500 ns ensemble. The average fraction of native contacts for the 500 ns ensemble was 0.959 ± 0.021.

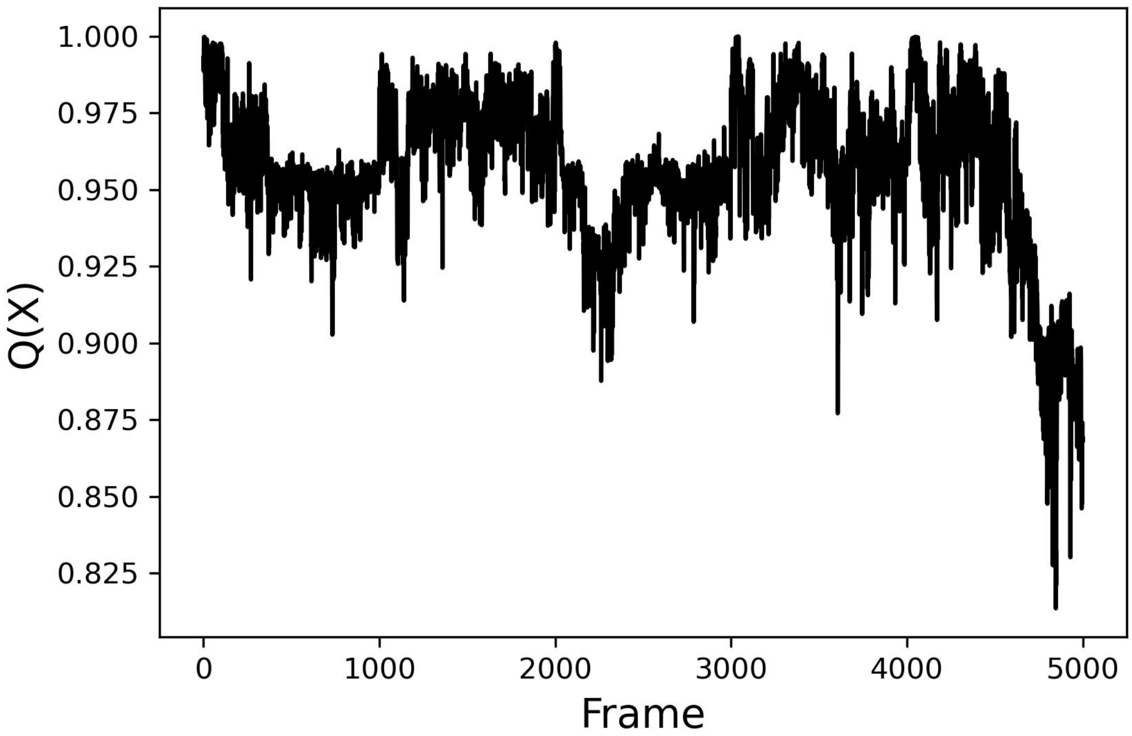


**Figure S11.** Native contact analysis in the interface of fosinoprilat in pose 3 bound complex 500 ns ensemble. The average fraction of native contacts for the 500 ns ensemble was 0.956 ± 0.024.

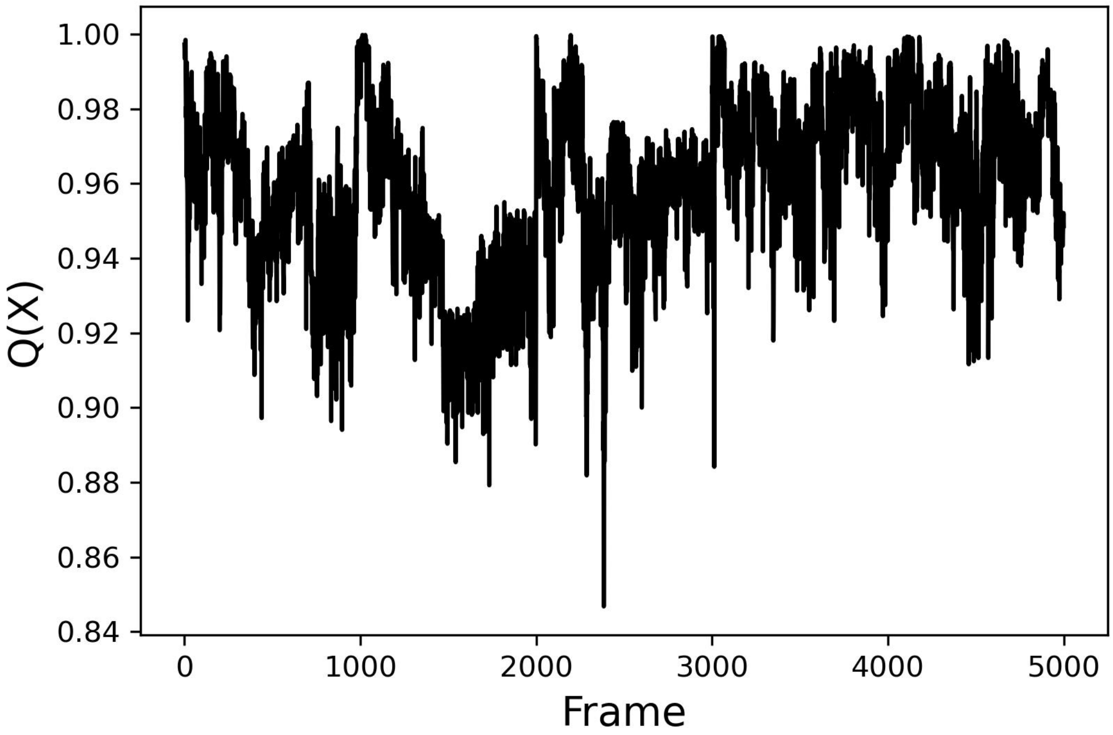


**Figure S12.** Native contact analysis in the interface of lisinopril bound complex 500 ns ensemble. The average fraction of native contacts for the 500 ns ensemble was 0.959 ± 0.022.

**Table S24.** Secondary structure plots of RBM residues for the apo complex.

| Residue Range 438 - 458 | Residue Range 459 - 474 |
|---|---|
| 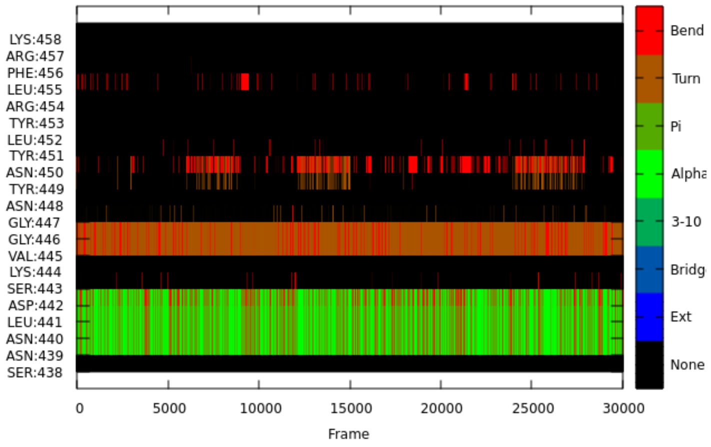 | 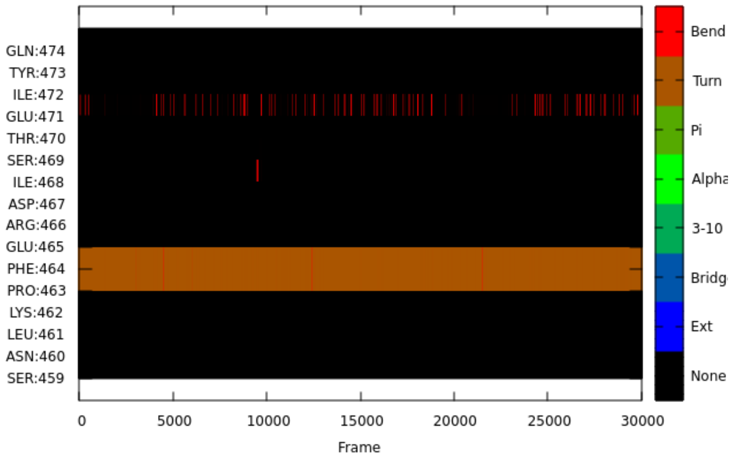 |

| Residue Range 475 - 490 | Residue Range 491 - 506 |
|---|---|
| 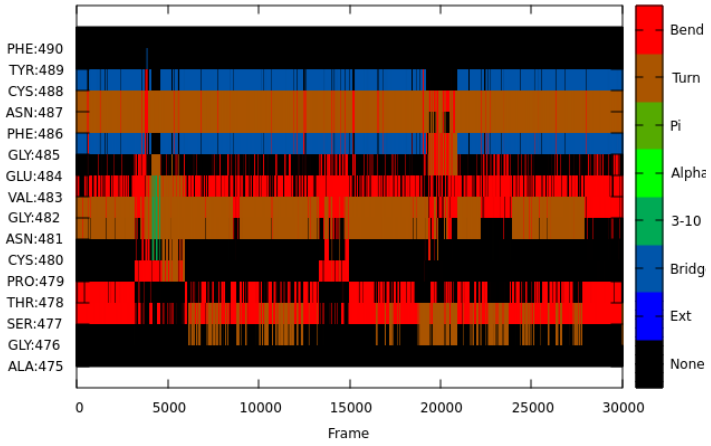 | 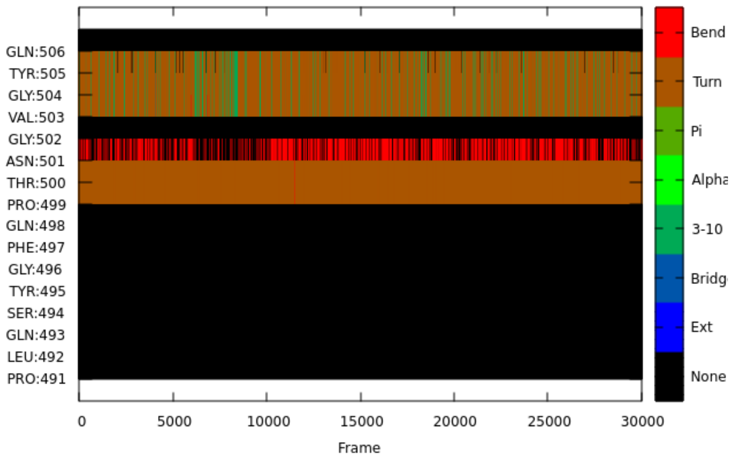 |





**Table S25.** Secondary structure plots of RBM residues for the fosinopril bound complex.

| Residue Range 438 - 458 | Residue Range 459 - 474 |
|---|---|
| 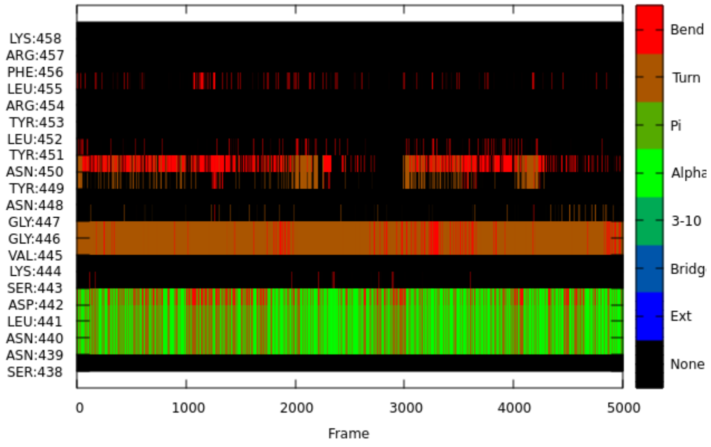 | 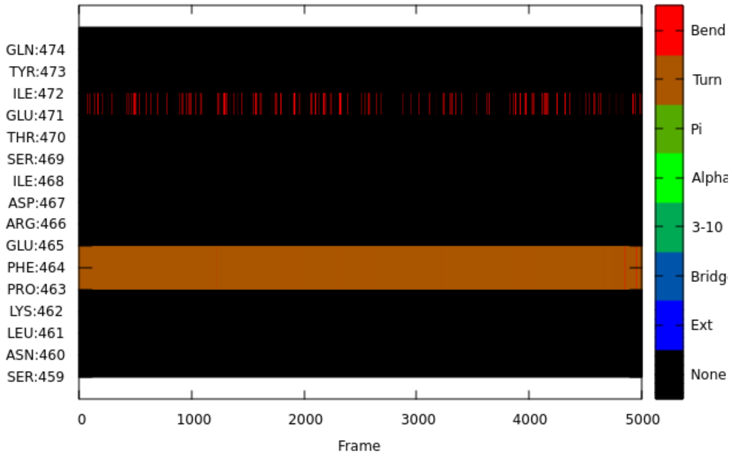 |
| Residue Range 475 - 490 | Residue Range 491 - 506 |

<table>
<tr>
<td>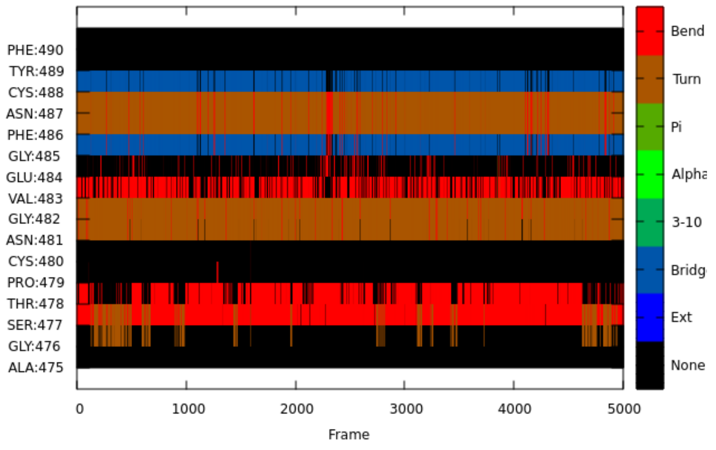
</td>
<td>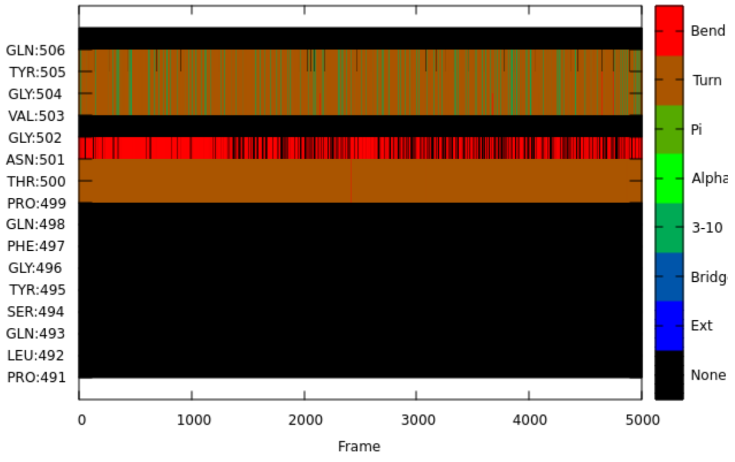
</td>
</tr>
</table>

**Table S26.** Secondary structure plots of RBM residues for the fosinoprilat pose 2 bound complex.

<table>
<tr>
<th>Residue Range 438 - 458</th>
<th>Residue Range 459 - 474</th>
</tr>
<tr>
<td>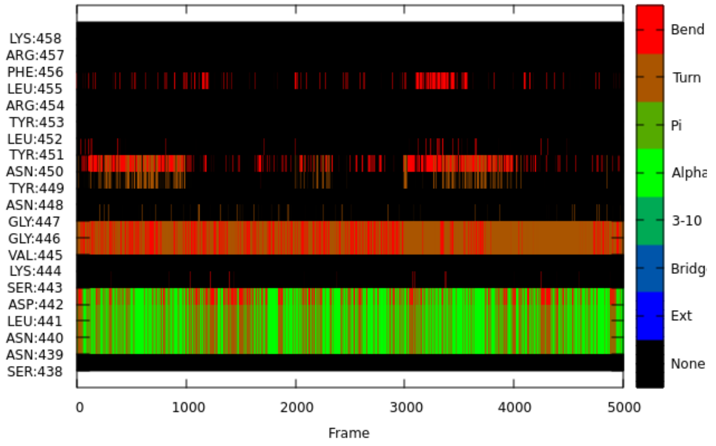
</td>
<td>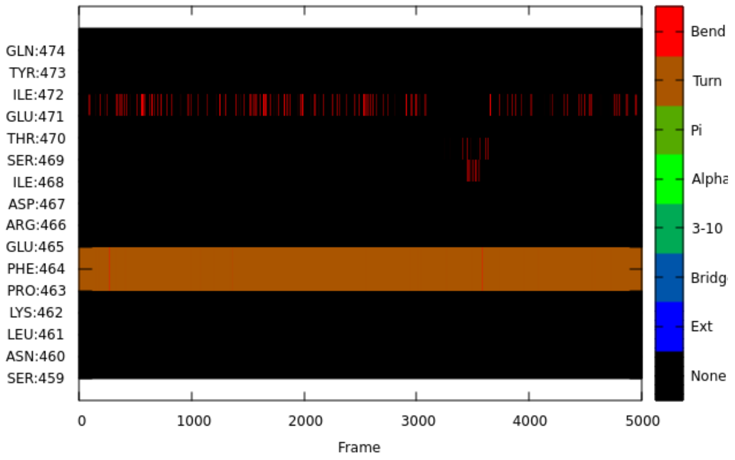
</td>
</tr>
<tr>
<th>Residue Range 475 - 490</th>
<th>Residue Range 491 - 506</th>
</tr>
</table>

| | |
|---|---|
| 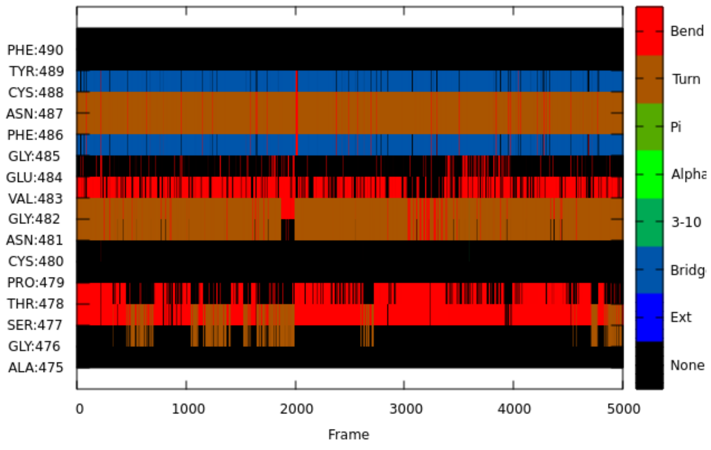 | 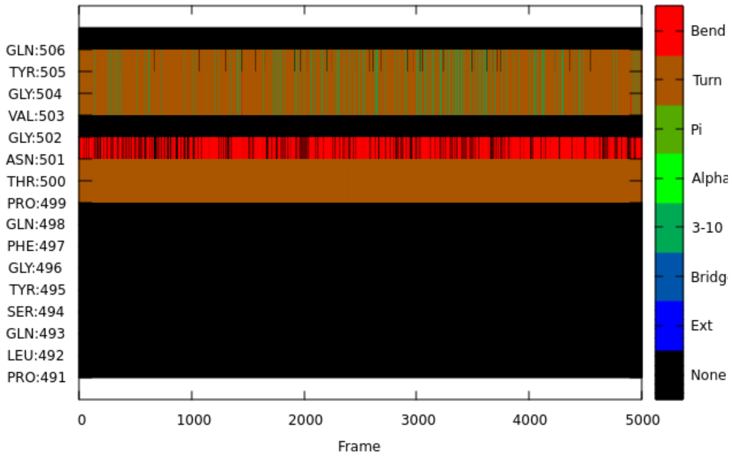 |

**Table S27.** Secondary structure plots of RBM residues for the fosinoprilat pose 3 complex.

| **Residue Range 438 - 458** | **Residue Range 459 - 474** |
|---|---|
| 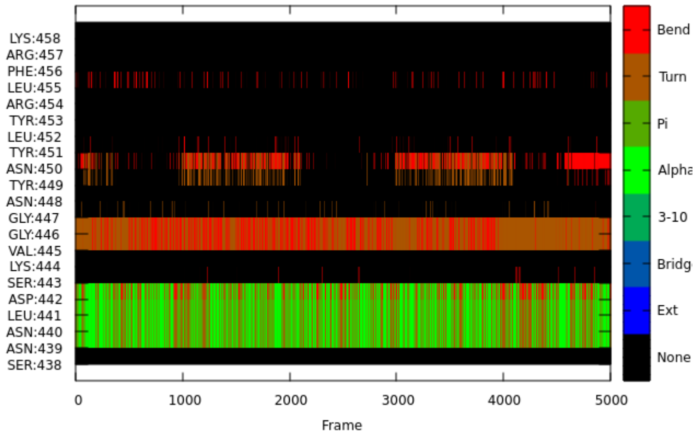 | 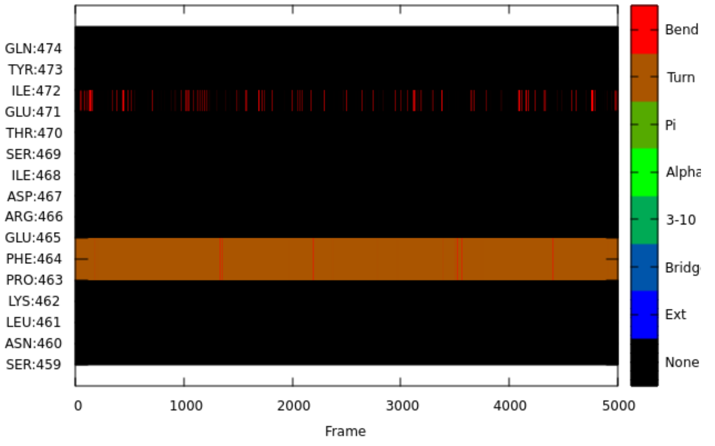 |
| **Residue Range 475 - 490** | **Residue Range 491 - 506** |

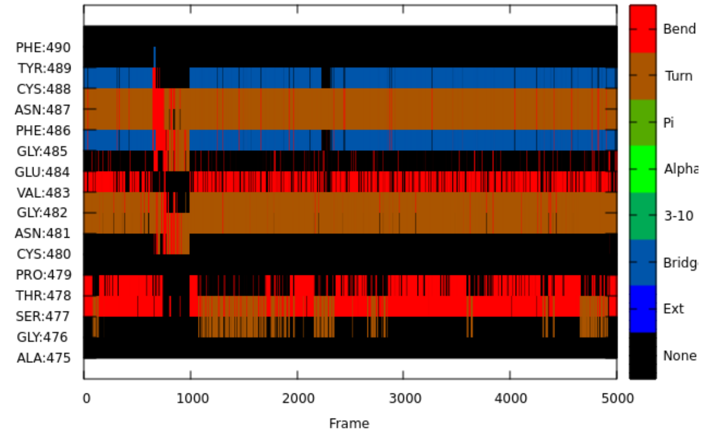


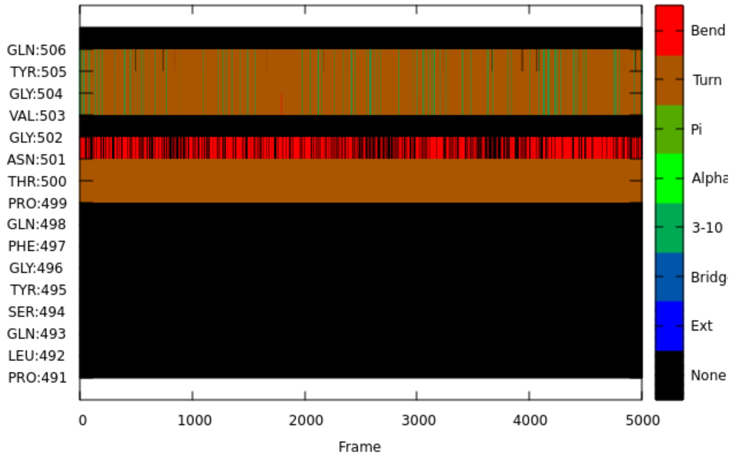


**Table S28.** Secondary structure plots of RBM residues for the lisinopril bound complex.

| Residue Range 438 - 458 | Residue Range 459 - 474 |
|---|---|

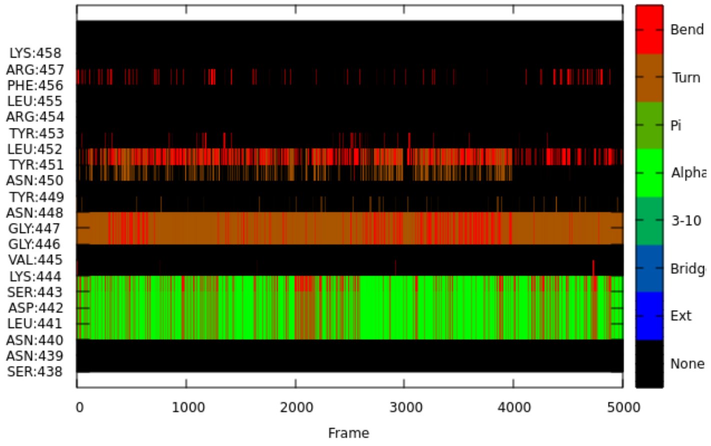


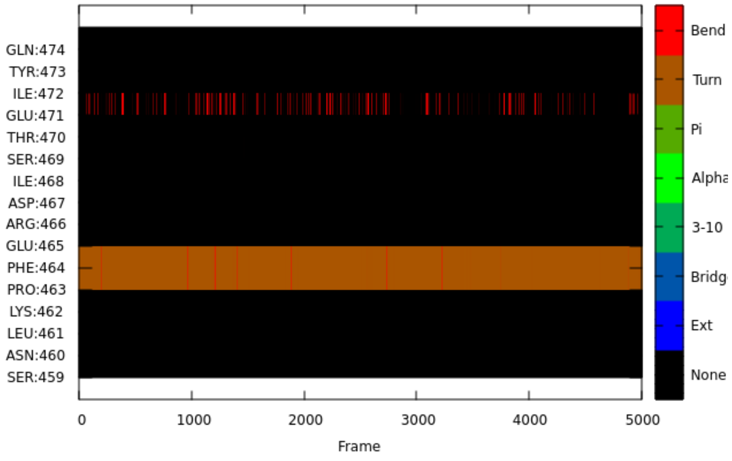


| Residue Range 475 - 490 | Residue Range 491 - 506 |
|---|---|

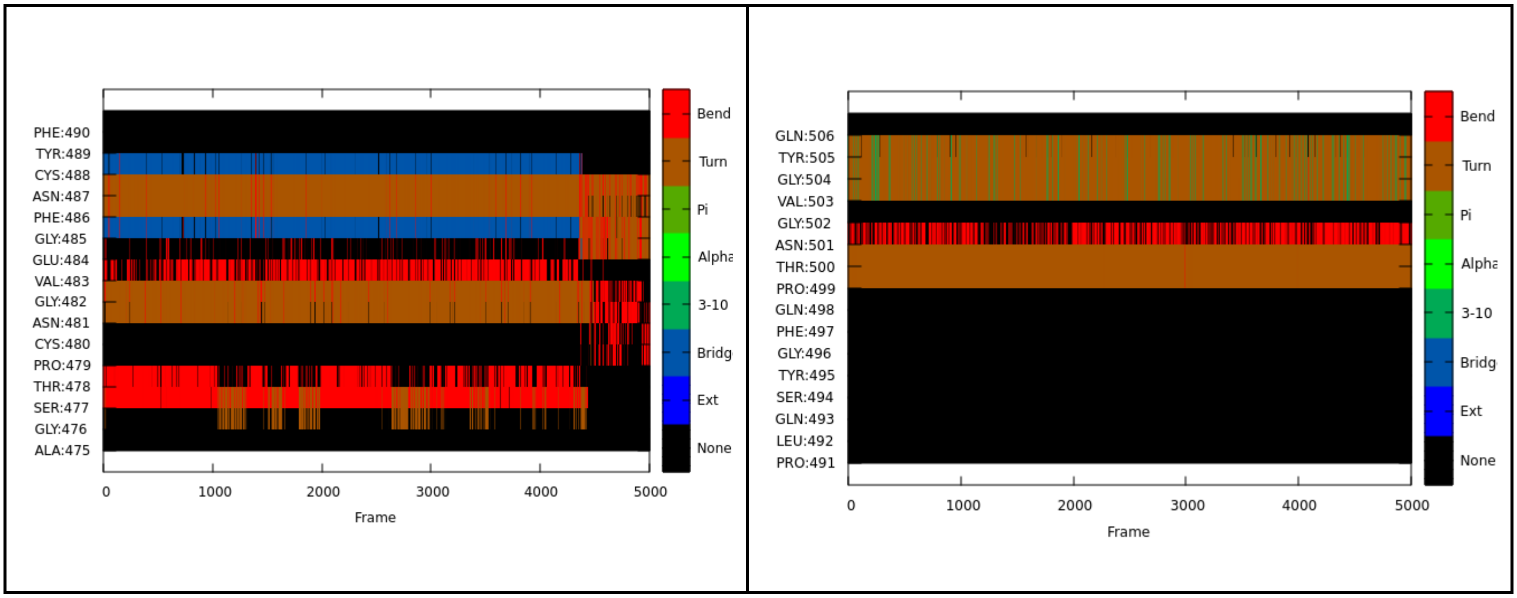


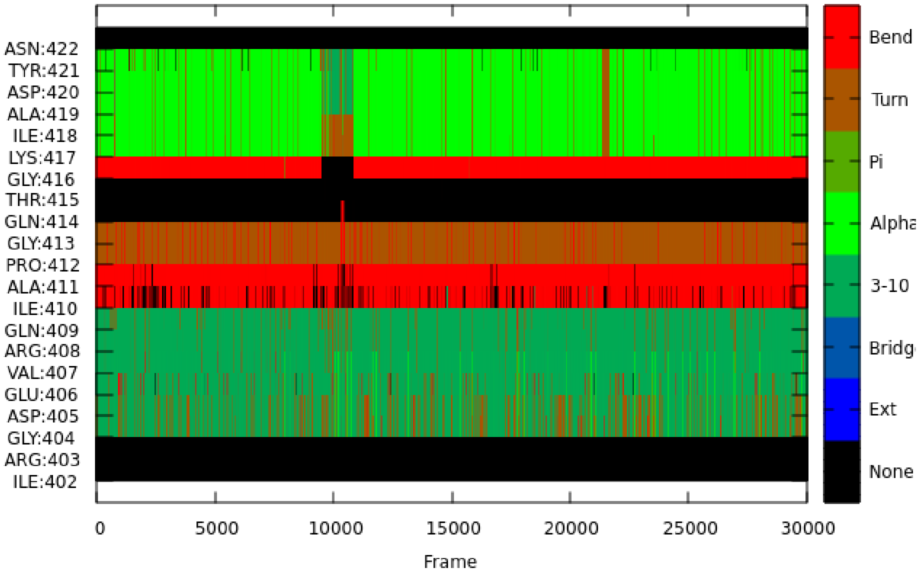


**Figure S13.** Secondary structure plot of residues 402 - 422 for the apo complex.

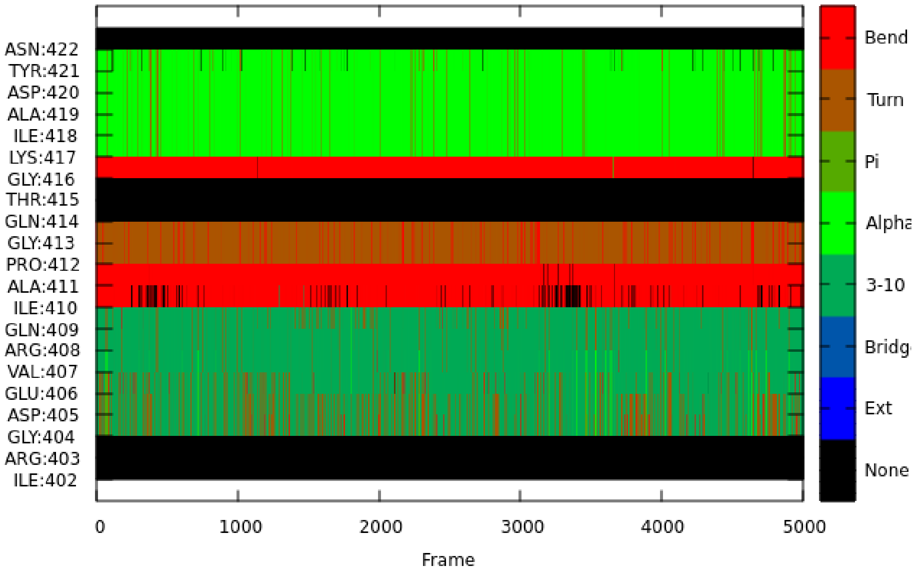


**Figure S14.** Secondary structure plot of residues 402 - 422 for the fosinopril bound complex.

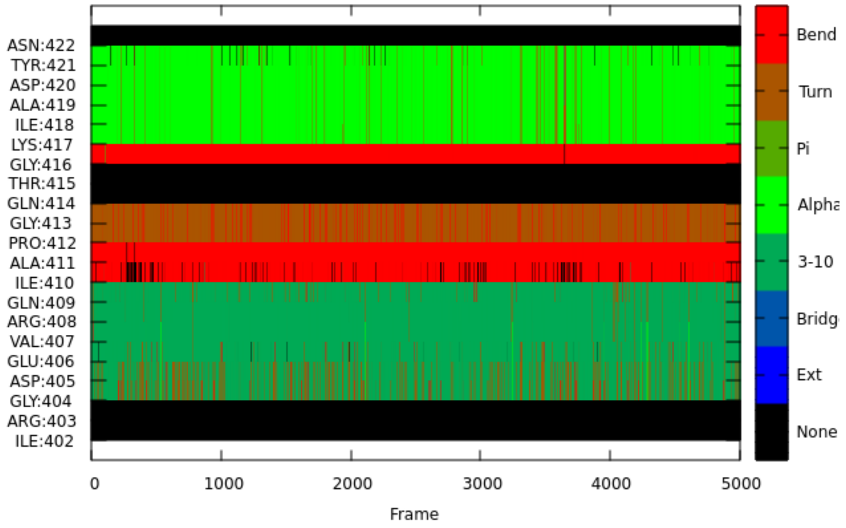


**Figure S15.** Secondary structure plot of residues 402 - 422 for the fosinoprilat pose 2 bound complex.

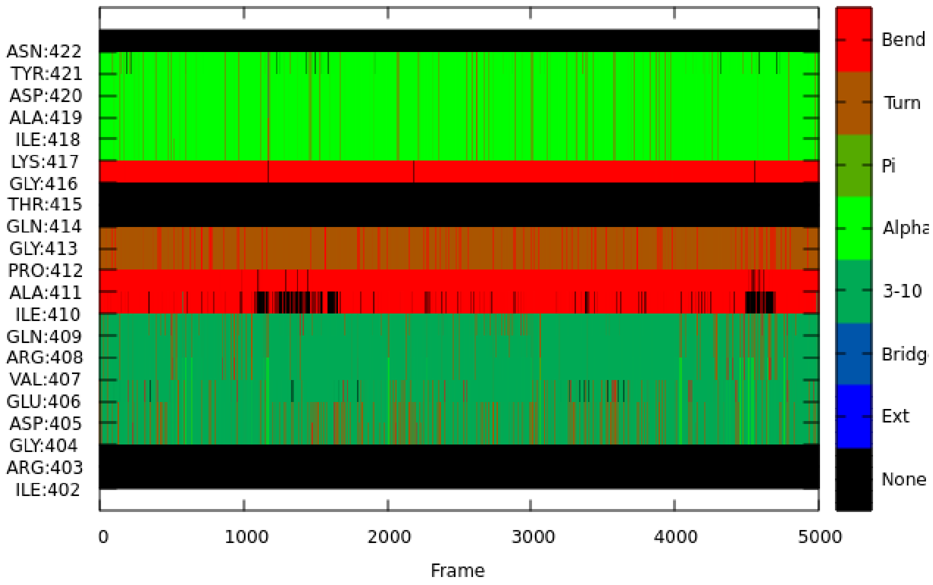


**Figure S16.** Secondary structure plot of residues 402 - 422 for the fosinoprilat pose 3 bound complex.

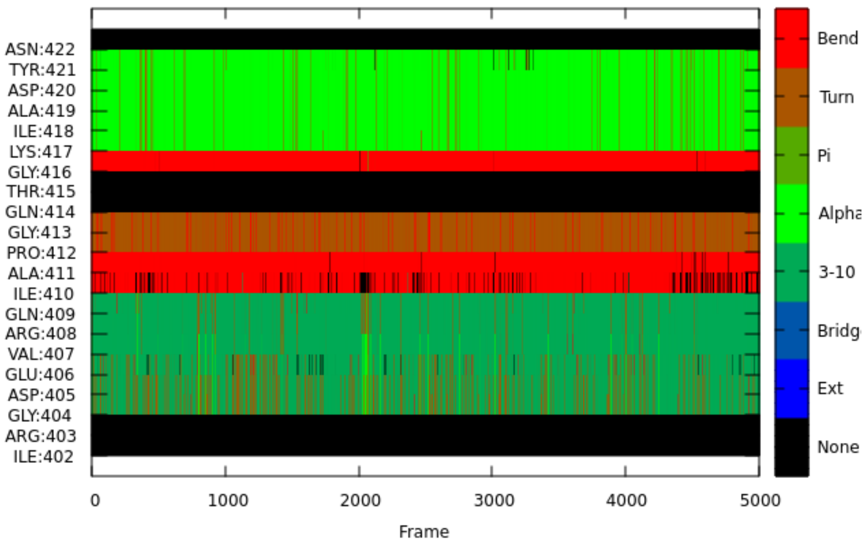


**Figure S17.** Secondary structure plot of residues 402 - 422 for the lisinopril bound complex.

Tables S24 - S28 contain the secondary structure plots for the apo and ligand-bound complexes for the residue range Ser438 - Gln506. For all ligand-bound complexes, there is a decrease in 3-10 helical occurrence for the residues Pro479 - Val483; however, notably, the helical structure of the apo complex was not present in a significant number of frames

throughout the 3 μs ensemble. There is a notable decrease in bend occurrence for the residue Gly482 for all ligand-bound complexes, with 23.93% as the apo ensemble occurrence and 3.82, 7.68, 4.86, and 8.24% as the fosinopril, fosinoprilat pose 2, fosinoprilat pose 3, and lisinopril ligand-bound ensemble occurrences, respectively. Additionally, there is an increase in turn occurrence for the residues Cys481 and Gly482 for the ligand-bound ensembles. The turn occurrences for the residues Cys481 in the apo ensemble is 74.22%, while the turn occurrence of this residue for fosinopril, fosinoprilat pose 2, fosinoprilat pose 3, and lisinopril ligand-bound ensemble are 96.18, 92.24, 94.80, and 86.86%, respectively. Similarly, turn occurrences for the residues Gly482 in the apo ensemble is 73.93%; while the turn occurrence of this residue for fosinopril, fosinoprilat pose 2, fosinoprilat pose 3, and lisinopril ligand-bound ensemble are 96.18, 92.24, 91.72, and 86.84%. Also, for the fosinopril ligand-bound ensemble, we noticed an increase in the bend occurrence for Asn450, where the occurrence increased from 28.99% in the apo ensemble to 43.34%. These differences in secondary structure occurrence could be caused by the ligand in the interface. The additional interactions between the ligand and the RBD could decrease the stability of the RBD structure by causing a decrease in helical and bend occurrence; however, it may have stabilizing effects by increasing the turn occurrence in all ligand-bound ensembles for the previously mentioned residues. To confirm the effect of the ligand on the RBD structure, it would be beneficial to extend the MD simulations in future studies.

Interestingly, the average position of each ligand is not near the previously mentioned amino acids where we notice a change in secondary structure, so we also decided to run a secondary structure analysis on the residue range Ile402 to Asn422. This range includes RBD amino acids that were found to have significant hydrogen bonding interactions with each ligand. Figures SD - SH are the secondary structure plots of the residue range Ile402 to Asn422 for the apo and ligand-bound complexes. From this analysis, we did not identify a significant change in secondary structure and believe that the extension of the ligand-bound MD simulations would be necessary to conclude the ligands' effect on the structure of the residues 402 to 422.

**Table S29**. Fosinopril water-mediated interactions. This table includes the percentage of water-mediated interactions between fosinopril and RBD residues or fosinopril and hACE2 residues. In a few cases, the water bridges an interaction between two residues and fosinopril; in this case, two residues are listed.

| Residues involved in a water-mediated hydrogen bond | Hydrogen Bond Percent Occurrence |
|---|---|
| RBD Asp405 | 29.44 |
| RBD Glu406 | 15.44 |
| RBD Asp405 and RBD Gln409 | 9.98 |
| RBD Arg403 | 9.88 |

| | |
|---|---|
| hACE2 Ala387 | 8.98 |
| RBD Gln409 | 7.66 |
| RBD Arg408 | 7.52 |
| hACE2 Glycan | 5.62 |

**Table S30**. Fosinoprilat Pose 2 water-mediated interactions. This table includes the percentage of water-mediated interactions between fosinoprilat pose 2 and RBD residues or fosinoprilat pose 2 and hACE2 residues. In a few cases, the water bridges an interaction between two residues and fosinoprilat pose 2; in this case, two residues are listed.

| Residues involved in the water-mediated hydrogen bond | Hydrogen Bond Percent Occurrence |
|---|---|
| RBD Glu406 | 72.24 |
| hACE2 Ala387 | 44.80 |
| hACE2 Asp30 | 36.14 |
| RBD Arg403 | 11.92 |
| hACE2 Arg393 | 11.12 |
| RBD Lys417 | 10.50 |
| RBD Arg408 | 9.44 |
| hACE2 Glycan | 9.06 |
| hACE2 His34 | 7.40 |
| RBD Glu406 and RBD Arg408 | 7.24 |
| hACE2 Asp30 and hACE2 Asn33 | 6.54 |
| RBD Tyr505 | 5.80 |
| hACE2 Gln388 and hACE2 Arg393 | 5.40 |

**Table S31**. Fosinoprilat Pose 3 water-mediated interactions. This table includes the percentage of water-mediated interactions between fosinoprilat pose 3 and RBD residues or fosinoprilat pose 3 and hACE2 residues. In a few cases, the water bridges an interaction between two residues and fosinoprilat pose 3; in this case, two residues are listed.

| Residues involved in the water-mediated | Hydrogen Bond Percent Occurrence |
|---|---|

| hydrogen bond | |
|---|---|
| hACE2 Asp30 | 29.08 |
| RBD Glu406 | 22.30 |
| RBD Asp405 | 17.20 |
| RBD Arg408 | 16.58 |
| RBD Lys417 | 14.40 |
| hACE2 Ala387 | 10.96 |
| hACE2 Glycan | 9.86 |
| hACE2 His34 | 7.38 |
| RBD Arg403 | 7.28 |
| hACE2 Asp30 and hACE2 Asn33 | 5.98 |
| hACE2 Asp30 and hACE2 His34 | 5.56 |

**Table S32.** Lisinopril water-mediated interactions. This table includes the percentage of water-mediated interactions between lisinopril and RBD residues or lisinopril and hACE2 residues. In a few cases, the water bridges an interaction between two residues and lisinopril; in this case, two residues are listed.

| Residues involved in the water-mediated hydrogen bond | Hydrogen Bond Percent Occurrence |
|---|---|
| RBD Glu406 | 33.72 |
| hACE2 Ala387 | 20.42 |
| hACE2 Asp30 | 14.06 |
| RBD Arg408 | 10.74 |
| RBD Arg403 | 10.12 |
| RBD Lys417 | 9.14 |
| hACE2 Glycan | 8.54 |
| hACE2 His34 | 8.24 |

| | |
|---|---|
| RBD Asp405 | 5.82 |
| RBD Asp405 and RBD Gln409 | 5.50 |
